# The DNMT1 CXXC domain senses CpG islands for DNA methylation inhibition

**DOI:** 10.1038/s41467-026-77872-8

**Published:** 2026-09-18

**Authors:** Jian Fang, Bo Pan, Wendan Ren, Jiuwei Lu, Ting Zhao, Yiran Guo, Ling Cai, Yinsheng Wang, Gang Greg Wang, Jikui Song

**Affiliations:** 1 Department of Biochemistry, University of California, Riverside, CA USA; 2 Department of Pharmacology and Cancer Biology, Duke University School of Medicine, Durham, NC USA; 3 Department of Pathology, Duke University School of Medicine, Durham, NC USA; 4 Environmental Toxicology Graduate Program, University of California, Riverside, CA USA; 5 Duke Cancer Institute, Duke University School of Medicine, Durham, NC USA; 6 Department of Chemistry, University of California, Riverside, CA USA

**Keywords:** DNA methylation, Cryoelectron microscopy, Nucleosomes

## Abstract

DNA methyltransferase DNMT1 plays an essential role in maintaining DNA methylation homeostasis. While DNMT1-mediated DNA methylation mainly occurs outside the active chromatin domains, such as CpG islands, how DNMT1 interplays with hypomethylated nucleosome environment to restrict CpG island methylation remains unclear. Here we report the structure of DNMT1 with a nucleosome containing unmodified CpG sites. The interaction of the DNMT1 CXXC domain with the unmodified CpG sites within the nucleosome core or the linker strengthens the DNMT1-nucleosome association, which contributes to the enrichment of DNMT1 in CpG islands. On the other hand, the CXXC domain is positioned in a manner that blocks the target recognition domain of DNMT1 from accessing potential DNA substrates. Consistently, disrupting the CXXC-CpG interaction leads to DNMT1 hyperactivity on nucleosomes with unmodified CpG sites, or those with spatially proximate unmodified and hemimethylated CpG sites. Furthermore, our whole-genome bisulfite-sequencing analysis reveals that introducing the CXXC-CpG interaction-defective DNMT1 mutation leads to substantial increase in CpG island methylation but not so much in overall genomic methylation, causing severe defects in transcriptional programming and stem cell differentiation. Together, this study establishes a role for the CXXC domain in safeguarding the CpG island hypomethylation, vital to DNA methylation homeostasis during development.

## Abstract (web summary)

DNA methylation patterns must be maintained while protecting CpG islands from inappropriate methylation. Here, the authors show that the DNMT1 CXXC domain binds unmethylated CpG sites on nucleosomes to block DNMT1 activity, thereby safeguarding CpG island hypomethylation and normal stem cell differentiation.

## Introduction

DNA methylation is an evolutionarily conserved mechanism that profoundly influences gene expression, genome stability and cell lineage commitment^1–4^. In mammals, DNA methylation mainly occurs at the C5 position of cytosines within the CpG dinucleotides, critically maintained by DNA methyltransferase 1 (DNMT1)^5,6^ and regulatory factor ubiquitin-like with PHD and RING finger domains 1 (UHRF1)^7,8^. Increasing evidence has indicated a role for the chromatin environment in regulating DNA methylation homeostasis across the genome^9–11^. For instance, DNMT1 shows the highest maintenance DNA methylation activity, even de novo DNA methylation activity, in transposable elements enriched with histone H3 lysine 9 trimethylation (H3K9me3), but the least activity in hypomethylated environments, such as CpG islands (CGIs)^10,11^. To date, how DNMT1 interplays with chromatin environment to orchestrate dynamic DNA methylation remains poorly understood.

**Fig. 1:**
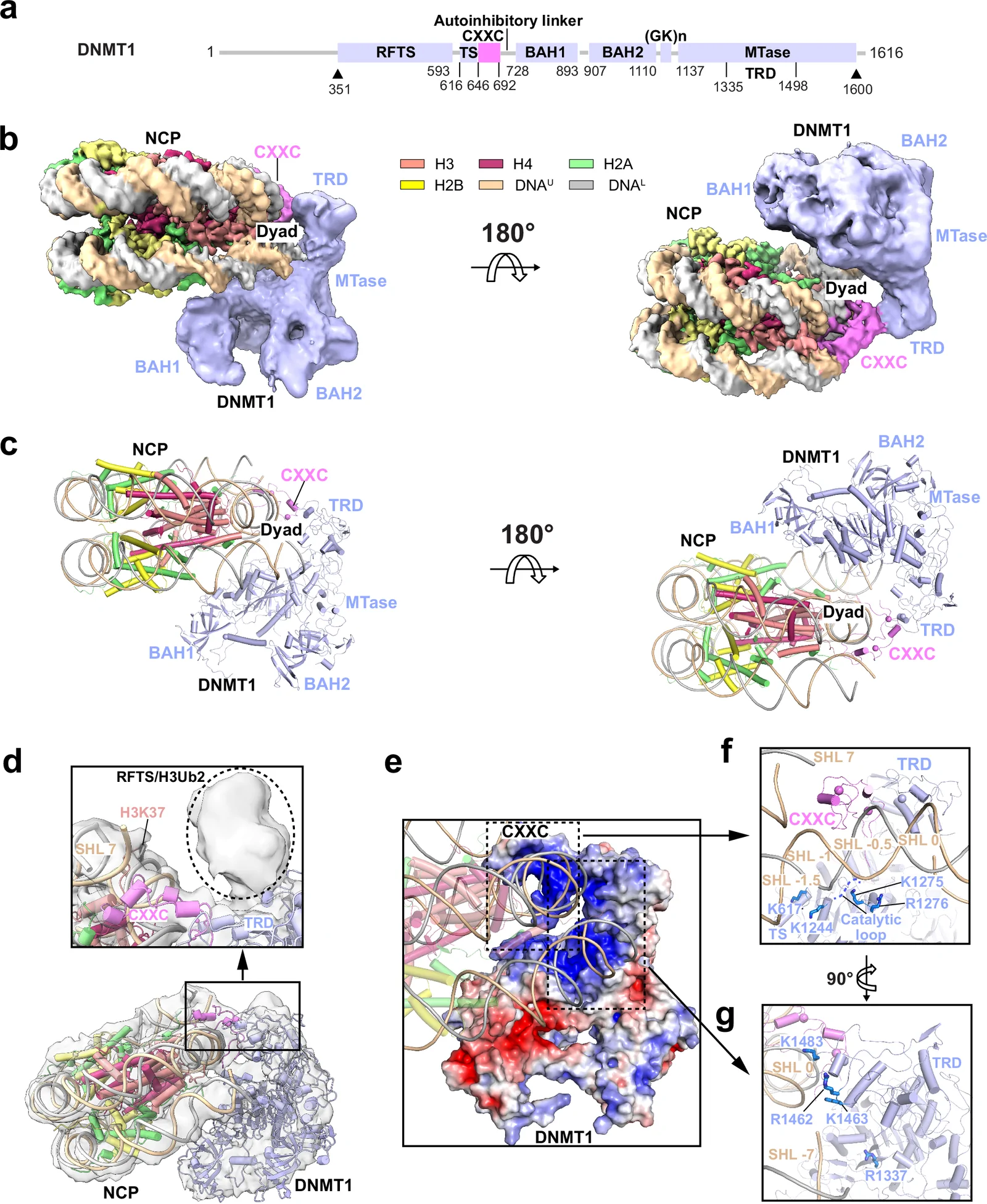
Structure of the DNMT1-^um^NCP complex. **a** Domain architecture of DNMT1, with individual domains delimited by residue numbers. TS, toggle switch. (GK)n, glycine-lysine repeat. The DNMT1_351–1600_ fragment used for structural study is indicated by arrows. **b** Two opposite views of the composite map (State I) for the DNMT1-^um^NCP complex, with individual domains of DNMT1 and the nucleosome dyad labeled. Histone H3, H4, H2A, H2B, and the individual strands of nucleosomal DNA (denoted as DNA^U^ and DNA^L^, respectively) are color coded. The sample color scheme is applied to the rest of the figures of this study, unless indicated otherwise. **c** Two opposite views of the atomic model of the DNMT1-^um^NCP complex (State I). **d** Overlaid density map and atomic model of the State-III DNMT1-^um^NCP complex, with the density corresponding to the RFTS/H3Ub2 components circled and its surrounding region annotated in the expanded view. Electrostatic surface view of DNMT1_351–1600_ bound to the NCP (**e**), with the NCP contact by the MTase core (**f**) and TRD (**g**) shown in the expanded views on right. The DNMT1 residues involved in interacting with nucleosomal SHL −1.5 to 0 and SHL -7 are shown in stick representation. The zinc ions within the CXXC domain and the TRD are shown in sphere representation.

DNMT1 is a multi-domain protein (Fig. 1a), containing a C-terminal methyltransferase (MTase) domain preceded by a large regulatory region, which includes an intrinsically disordered N-terminal tail, a replication-foci-targeting sequence (RFTS) domain, a regulatory element controlling conformational switch of the catalytic site between active and inactive states (denoted as toggle switch herein), a CXXC zinc-finger domain, and a pair of Bromo-Adjacent Homology (BAH) domains, and glycine-lysine (GK) dipeptide repeat^12–15^. It has been established that the DNMT1 RFTS domain inhibits the MTase domain by restricting DNA binding^16,17^, whereas its interaction with the UHRF1-catalyzed dual mono-ubiquitylation of PCNA-associated factor 15 or histone H3 (H3Ub2) helps relieve the autoinhibition and recruit DNMT1 to replication foci during the early and late S phase, respectively^14,18–21^. Furthermore, the respective recognition of H3K9me3 and histone H4 lysine 20 trimethylation (H4K20me3) by the RFTS domain and the first BAH (BAH1) domain of DNMT1 contributes substantially to heterochromatin-specific DNA methylation on chromatin^22,23^.

The DNMT1 CXXC domain belongs to a family of zinc-finger domains that specifically recognize unmodified CpG-containing DNA^24,25^. Our previous study demonstrated that its interaction with unmodified CpG-containing DNA leads to an autoinhibitory conformation of DNMT1, in which the CXXC domain and its downstream linker (a.k.a. autoinhibitory linker) are positioned between DNA and the target recognition domain (TRD) of DNMT1, thereby blocking the unmodified CpG DNA from entering the active site of DNMT1^26^. A more recent study further indicated that such a CXXC domain-mediated DNMT1 autoinhibition contributes to substrate specificity of DNMT1 upon enzyme-substrate association^27^. Whereas these observations support a model that the CXXC-CpG interaction inhibits DNMT1-mediated de novo DNA methylation^26^, such notion was challenged by an observation that *Dnmt1*-knockout mouse embryonic stem cells (1KO-mESCs), reconstituted with wild-type (WT) DNMT1 or its CXXC-lacking mutant, led to a similar level of genomic DNA methylation^28^. Therefore, the precise role of the CXXC domain in DNMT1-mediated DNA methylation remains unknown.

To assess impact of the hypomethylated nucleosome environment on DNMT1-mediated DNA methylation, we determined the structure of DNMT1 in complex with an H3Ub2-modified nucleosome core particle (NCP) harboring unmodified CpG sites, representing the product of UHRF1-catalyzed substrate. Our integrated structural, biochemical and genomic analyses demonstrate that recognition of unmodified CpG within the NCP by the DNMT1 CXXC domain leads to an autoinhibitory conformation, in which CXXC is positioned to block substrate access of the TRD, resembling what was observed for the DNMT1-CpG DNA complex^26^. Disrupting the CXXC-CpG interaction not only increases de novo DNA methylation activity of DNMT1 but also elevates DNMT1’s maintenance methylation activity on the hemimethylated CpG (hemiCG) site proximal to the unmodified CpG sites in *cis* or *trans*. Furthermore, using the 1KO-mESCs reconstituted with the CXXC-CpG interaction-defective mutant of DNMT1, we observe substantial DNA hypermethylation at genomic sites enriched for CGIs, as well as severe defects in transcriptional programming and stem cell differentiation. Together, this study establishes a role for the DNMT1 CXXC domain, through environment sensing and DNMT1 autoinhibition, in safeguarding the hypomethylated environment at CGIs, thereby ensuring proper cell differentiation and lineage specification.

## Results

### Structural determination of DNMT1 bound to the NCP with unmodified CpG sites

To illustrate how the DNMT1 CXXC domain interplays with the RFTS-H3Ub2 regulatory axis in a hypomethylated chromatin environment, we set out to solve the cryo-EM structure of DNMT1 with unmethylated NCP (^um^NCP), focusing on DNMT1’s C-terminal fragment spanning from the RFTS domain to the MTase domain (residues 351–1600; DNMT1_351–1600_) (Fig. 1a). The NCP was modified with dual mono-ubiquitylation at the H3 lysines 18 and 23 to mimic the product of UHRF1-mediated ubiquitylation^18^, and H4K20me3 to strengthen the DNMT1 interaction (Supplementary Fig. 1a, b)^23^. After 3D classification and refinement, we solved the structure of the DNMT1_351–1600_-^um^NCP complex at 3.12-Å overall resolution, 3.02-Å local resolution for the DNMT1 CXXC domain-^um^NCP component, and 4.86-Å local resolution for the rest of DNMT1_351–1600_ (State I) (Supplementary Fig. 1c–g and Supplementary Table 1). To clarify conformation of the DNMT1 molecule, following the initial 3D classification, we also performed DNMT1_351–1600_-masked 3D classification, leading to a density map of the DNMT1_351–1600_-^um^NCP complex (State II) at resolution of 3.84 Å (Supplementary Fig. 1c, h). Structural alignment of the State-I and State-II DNMT1-^um^NCP complexes reveals nearly identical conformation for the CXXC-^um^NCP moiety, yet a subtle conformational swing for the rest of DNMT1, in line with the high conformational dynamics of the latter (Supplementary Fig. 1i). For high-resolution characterization of the CXXC-NCP interaction, we focus on State I for structural analysis in this study.

The density map of the CXXC-NCP components permits us to trace both the CXXC domain and the NCP reliably (Fig. 1c and Supplementary Fig. 2a–f). In addition, we were able to model the BAH1, BAH2 and MTase domains of DNMT1 (Fig. 1c and Supplementary Fig. 2g). On the other hand, the RFTS domain and its C-terminal linker (residues 351–615), the toggle switch (except for residues 616–622), the autoinhibitory linker, and the loop harboring the catalytic cysteine C1226 (a.k.a. catalytic loop: residues 1221–1243), were disordered. Nevertheless, further classification of the DNMT1_351–1600_-^um^NCP particles led to identification of the density corresponding to the DNMT1 RFTS domain and H3Ub2 components (State III), positioned between the DNMT1 TRD and superhelical location (SHL) 7, proximal to the H3 N-terminal tail (Fig. 1d and Supplementary Fig. 1c, d). Such spatial proximity between the DNMT1 RFTS domain and the H3 tail supports the notion that strong interaction between the DNMT1 RFTS domain and H3Ub2 helps tether the DNMT1 molecule to the NCP^18^.

The structure of the DNMT1-^um^NCP complex reveals that the DNMT1 TRD and CXXC domains are positioned at the nucleosome dyad, while the BAH1 domain, BAH2 domain and the catalytic core of the MTase domain (MTase core) of DNMT1 arch over the histone surface (Fig. 1b, c). The NCP interaction of DNMT1 is mediated by multiple structural elements of DNMT1, including the CXXC domain, the MTase core, and the TRD (Fig. 1e–g). Notably, the CXXC domain adopts a bent-hairpin conformation to encircle nucleosomal DNA at the dyad (Fig. 1e), the MTase core provides several positively-charged residues (e.g., K1244, K1275 and R1276) located downstream of the disordered catalytic loop to engage the backbone of SHL −0.5 DNA (Fig. 1f), and the TRD also provides some positively-charged residues (e.g., R1337, R1462, K1463 and K1483) to interact with the backbone of SHL 0 or SHL -7 DNA (Fig. 1g). Additional DNMT1-NCP interaction involves residue K617 at the N-terminus of the toggle switch, which makes electrostatic contact with the SHL −1.5 DNA (Fig. 1f).

### Structural details of the DNMT1 CXXC domain-CpG interaction at the dyad

**Fig. 2:**
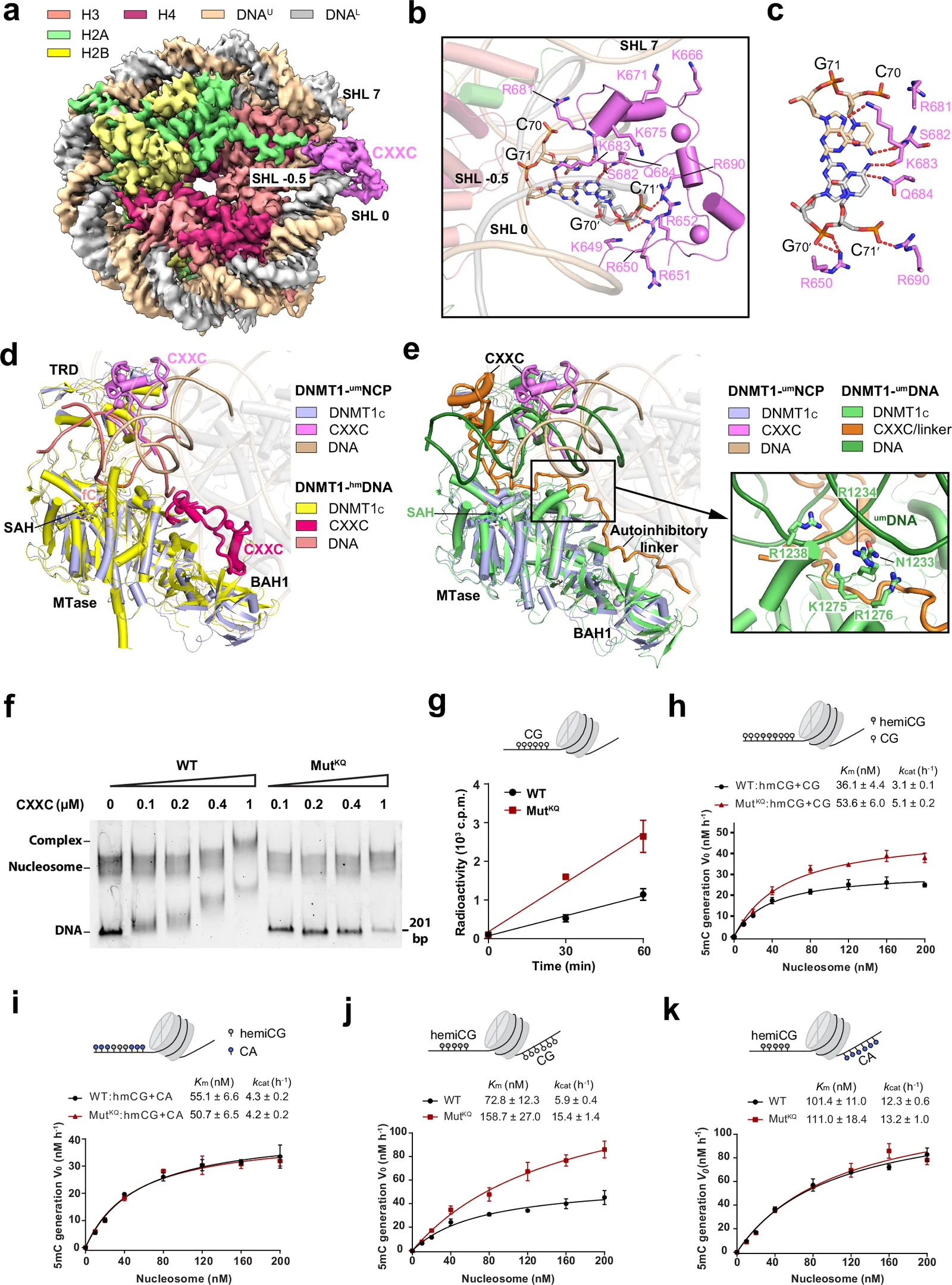
Structural and biochemical analysis of the DNMT1 CXXC domain-NCP interaction. **a** Local density map for the DNMT1 CXXC-^um^NCP component, with the CXXC domain and interacting SHL −0.5, 0 and 7 labeled. **b** Close-up view of the interaction between the DNMT1 CXXC domain and the nucleosomal DNA at SHL −0.5, 0 and 7. The DNA-interacting residues are labeled and shown in stick representation. The CpG dinucleotides at SHL −0.5 are labeled as C_70_ and G_71_ for the DNA^U^ strand and C_71_′ and G_70_′ for the DNA^L^ strand. The hydrogen bonds are shown as dashed lines. **c** Close-up view of the CpG-specific interaction by the DNMT1 CXXC domain. **d** Structural overlay of the DNMT1-^um^NCP complex and the DNMT1-^hm^DNA complex (PDB 7XI9), highlighting distinct positionings of the CXXC domain and DNA. DNMT1c denotes the DNMT1 fragment (except for the CXXC domain) downstream of the RFTS domain. The SAH molecule and the flipped fC nucleotide in the DNMT1-^hm^DNA complex are shown in stick representation. **e** Structural overlay between the DNMT1-^um^NCP complex and the DNMT1-^um^DNA complex (PDB 3PTA), with the close-up view of the MTase-DNA interaction in the DNMT1-CG DNA complex shown in the expanded view. **f** EMSA analysis of the binding of the DNMT1 CXXC domain, WT or Mut^KQ^, to the 601 DNA and the NCP. The experiment was repeated twice with consistent results. **g** DNA methylation kinetics of DNMT1_351–1600_, WT or Mut^KQ^, on the CpG-containing nucleosome substrate. Data are mean ± s.d. (*n* = 3 biological replicates). **h,i** Steady-state kinetics of DNMT1_351–1600_, WT or Mut^KQ^, on the nucleosome linker containing three hemiCG sites flanked by unmethylated CpG (hemiCG+CG) (**h**) or CpA (hemiCG+CA) (**i**) sites. Data are mean ± s.d. (*n* = 3 biological replicates). **j,k** Steady-state DNA methylation kinetics of DNMT1_351–1600_, WT or Mut^KQ^, on one nucleosome linker containing multiple hemiCG sites and the other linker containing multiple unmethylated CpG (**j**) or CpA (**k**) sites. Data are mean ± s.d. (*n* = 3 biological replicates). Source data are provided with this paper.

The Widom 601 DNA used for the NCP assembly contains 13 symmetric CpG sites (Supplementary Fig. 2h)^29^. Detailed structural analysis of the DNMT1-^um^NCP complex reveals that the DNMT1 CXXC domain spans from SHL 0 to SHL −0.5, engaging base-specific interactions with the unmodified CpG site at SHL −0.5 (Fig. 2a–c). The fact that the CXXC domain failed to interact with the rest of the CpG sites is presumably due to the reduced accessibility of those sites (Supplementary Fig. 2h). Of note, the sidechain amino group of CXXC residue K683 donates a hydrogen bond to the N7 atom of the CpG guanine (G_71_) on upper strand, while its backbone carbonyl group receives a hydrogen bond from the N4 atom of the CpG cytosine (C_71_′) from the lower strand (Fig. 2b, c). The sidechain amino group of CXXC residue Q684 forms a hydrogen bond with the O6 atom of CpG guanine (G_70_′) on the lower strand, and the backbone carbonyl group of CXXC residue S682 receives a hydrogen bond from the N4 atom of CpG cytosine (C_70_) on the upper strand (Fig. 2b, c). Furthermore, the side chains of CXXC residues R650, R681, and R690 interact with the backbone of C_71_′, G_70_′ and C_70_ through hydrogen-bonding and/or electrostatic contacts (Fig. 2b, c). Aside from the CpG site, CXXC residues K649, R650, R651 and R652 engage in electrostatic contact with the backbone of SHL 0 DNA, while CXXC residues K666 and K671 engage in electrostatic contact with the backbone of SHL 7 DNA (Fig. 2b). Together, these interactions lead to a buried surface area of ~1862 A^2^.

Structural comparison of the DNMT1-^um^NCP complex and the previously reported complex of a DNMT1 fragment (residues 646–1600) bound to an unmethylated CpG-containing DNA (PDB 3PTA) (DNMT1-^um^DNA)^26^ reveals well-aligned CXXC-CpG interaction (Supplementary Fig. 2j), indicative of an identical CpG-recognition mechanism. The major difference lies in two highly conserved residues, K666 and K671, which engage SHL 7 in the DNMT1-^um^NCP complex but are kept away from the DNA in the DNMT1-^um^DNA complex (Supplementary Fig. 2j, k), likely reflecting an evolutionary trait of the CXXC domain adapting to the DNA-dense environment of the chromatin.

Consistent with the structural observation, our electrophoretic mobility shift assay (EMSA) reveals that whereas increasing concentration of the DNMT1 CXXC domain gradually shifted the native 601 nucleosomes, replacement of the 5 dyad-adjacent CpG sites each with a non-CpG site led to notable decrease in the binding (Supplementary Fig. 2m).

### The CpG-bound CXXC domain blocks the DNMT1 TRD in a manner independent of the RFTS domain-mediated autoinhibition

To decipher the functional state of the DNMT1-^um^NCP complex, we compared the structure of the DNMT1-^um^NCP complex with the productive complex of a DNMT1 fragment (residues 351–1616) with a hemimethylated DNA (^hm^DNA) (PDB 7XI9), in which the central CpG site contains a 5-methylcytosine on the template strand and a 5-flurocytosine (fC) on the target strand^15^. In the DNMT1-^hm^DNA complex, the CXXC domain is docked on the BAH1 domain and the DNA molecule is deeply embraced by the MTase core and the TRD, with the fC nucleotide flipped into the active site^15,30^ (Fig. 2d). In stark contrast in the DNMT1-^um^NCP complex, the DNA molecule’s access to the TRD and MTase core is blocked by the CXXC domain, representing an autoinhibitory state of DNMT1 (Fig. 2d). Indeed, structural overlay of the DNMT1-^um^NCP complex with the autoinhibitory DNMT1-^um^DNA complex (PDB 3PTA)^26^ reveals that the CXXC domain is similarly positioned in front of the TRD (Fig. 2e). Aside from the CXXC-CpG interaction, the DNMT1-^um^NCP and DNMT1-^um^DNA complexes involve a similar set of positively charged residues in the MTase domain to interact with the CpG-flanking site (Fig. 2e), which helps stabilize the autoinhibitory state of DNMT1. On the other hand, the two complexes differ markedly in the positioning of the CXXC domain and bound DNA (Fig. 2e), highlighting the conformational flexibility of the CXXC domain in accommodating various positionings of the CpG sites. An additional difference lies in the conformation of the autoinhibitory linker, which traverses the MTase and BAH1 domains to block DNA access in the DNMT1-^um^DNA complex but becomes disordered in the DNMT1-^um^NCP complex (Fig. 2e).

Furthermore, we compared the structure of the DNMT1-^um^NCP complex with that of apo-form DNMT1_351–1600_ (apo-DNMT1) (PDB 4WXX). In apo-DNMT1, the RFTS domain packs against the TRD to block the DNA binding (Supplementary Fig. 2l)^17,31^. In contrast, the RFTS domain in the DNMT1-^um^NCP complex is repositioned near to the H3 tail, as described above, leading to releasing of the RFTS-mediated blockage of the TRD (Supplementary Fig. 2l). Despite the RFTS repositioning, DNMT1 in the DNMT1-^um^NCP complex remains in an autoinhibitory conformation owing to the CXXC-CpG interaction (Supplementary Fig. 2l), suggesting that DNMT1 is regulated by a multi-layered autoinhibitory mechanism that at least involves the independent blockades of TRD by the RFTS and CXXC domains.

Previously, the autoinhibitory linker of DNMT1 has been shown to stabilize an autoinhibitory conformation of the enzyme through its direct interaction with the MTase and BAH1 domains (Fig. 2e)^26,31^. To clarify the regulatory role of DNMT1’s autoinhibitory linker, we also solved the cryo-EM structure of a hyperactive mutant (Mut^MHKKKQ:^ M718A/H719A/K723A/K724A/K725A/Q726A) of DNMT1_351–1600_, in which the interaction between the autoinhibitory linker and the BAH1 domain was disrupted (Supplementary Fig. 3a, b)^23^, in complex with the ^um^NCP (Supplementary Fig. 3c–i and Supplementary Table 1). In comparison with WT DNMT1-^um^NCP, the Mut^MHKKKQ^ DNMT1-^um^NCP complex shows similar CXXC-NCP interaction and accordingly, an autoinhibitory conformation that contrasts with that of the DNMT1-^hm^DNA complex (PDB 7XI9) (Supplementary Fig. 4a–e). Also, the autoinhibitory linker remains disordered. However, much improved density was observed for the BAH domains, the catalytic loop and toggle switch (Supplementary Fig. 3h, i). Aside from the CXXC-CpG interaction, DNMT1’s MTase (MTase core and TRD) and toggle switch modules interact with the nucleosomal DNA in the Mut^MHKKKQ^ DNMT1-^um^NCP complex (Supplementary Fig. 4f–h), similar to what was seen with the WT DNMT1-^um^NCP complex (Fig. 1e). In addition, weak density for the RFTS-H3Ub2 was identified around the NCP-embedded H3 and the TRD (Supplementary Fig. 4i), again resembling what was observed for the WT DNMT1-^um^NCP complex (Fig. 1d). The major difference between the WT and Mut^MHKKKQ^ DNMT1-^um^NCP complex lies in that the NCP interaction by residues K1275 and R1276 in the MTase core, which was observed for the WT complex (Fig. 1e), is lacking in the Mut^MHKKKQ^ complex. As a result, the DNMT1 molecule in the Mut^MHKKKQ^ complex is positioned further away from the NCP surface than the WT complex (Supplementary Fig. 4d). These observations suggest that the CXXC-CpG interaction, supported by multifaceted DNA contact by the toggle switch, the TRD, and/or MTase core, critically underpins an autoinhibitory conformation of DNMT1, whereas the amino acid composition of the autoinhibitory linker likely fine-tunes the conformational dynamics of DNMT1.

### The CXXC-CpG interaction inhibits DNMT1-mediated linker methylation

To assess the impact of the CXXC-CpG interaction on DNMT1-mediated nucleosome methylation, we mutated the CpG-interacting residues K683 and Q684 each into alanine (Mut^KQ^). Our EMSA analysis indicated that introducing the Mut^KQ^ mutation substantially impaired the nucleosome or DNA binding of the CXXC domain (Fig. 2f). And our Bio-layer Interferometry (BLI) binding assay revealed that introducing the Mut^KQ^ mutation increased the dissociation constant (*K*_d_) of DNMT1_351–1600_ from 140 nM to 330 nM for the unmodified nucleosome, and from 1.3 nM to 2.3 nM for the H3Ub2-modified nucleosome (Supplementary Fig. 5a–e). These data support the roles of these two CXXC residues in CpG recognition at both DNA and nucleosome levels, particularly for unmodified nucleosomes.

Next, we measured in vitro DNA methylation kinetics of DNMT1_351–1600_, WT or the Mut^KQ^, on nucleosome substrates. Relative to WT DNMT1, the Mut^KQ^ mutant shows increased DNA methylation efficiency on nucleosomes with unmethylated linker CpG sites (Fig. 2g and Supplementary Fig. 5f), supporting a notion that the CXXC-DNA interaction inhibits DNMT1-mediated de novo DNA methylation^26,27^. Furthermore, we assessed the impact of spatially proximal CpG sites on maintenance DNA methylation using WT or Mut^KQ^ DNMT1 (Fig. 2h–k and Supplementary Fig. 5g–l). Introducing the Mut^KQ^ led to an increase in both *k*_cat_ (3.1–5.1 h^–1^) and *K*_m_ (36.1–53.6 nM) of DNMT1_351–1600_ on the substrates with the hemiCG sites flanked by unmodified CpG sites (Fig. 2h). In addition, we measured the DNA methylation kinetics of DNMT1_351–1600_ on the nucleosomes in which the linker hemiCG sites were flanked by unmodified CpA sites, revealing equivalent *k*_cat_ and *K*_m_ values, within the experimental error, for WT and Mut^KQ^ DNMT1 (Fig. 2i). These data suggest that a *cis* DNMT1 CXXC-CpG interaction, while promoting the DNMT1-nucleosome association, significantly impairs the DNMT1-mediated maintenance methylation of CpG dinucleotide located in the linker DNA. Likewise, on the nucleosome substrates in which the hemiCG and CpG sites are positioned on different linkers, the *k*_cat_ of WT DNMT1_351–1600_ is 2.6-fold lower than that of Mut^KQ^, but reached to a similar level to that of Mut^KQ^ when the CpG sites were replaced by the CpA sites (Fig. 2j, k), suggesting that a *trans* CXXC-CpG interaction also contributes to inhibition of DNMT1-mediated methylation.

These data suggest that the CXXC-CpG interaction inhibits DNMT1-mediated DNA methylation in both *cis* and *trans*, potentially restricting DNMT1’s activity in a hypomethylated environment.

### Unbiased genome-wide profiling unveils an uneven chromatin-targeting pattern of DNMT1, with a preferential binding to sites exhibiting high CpG density

**Fig. 3:**
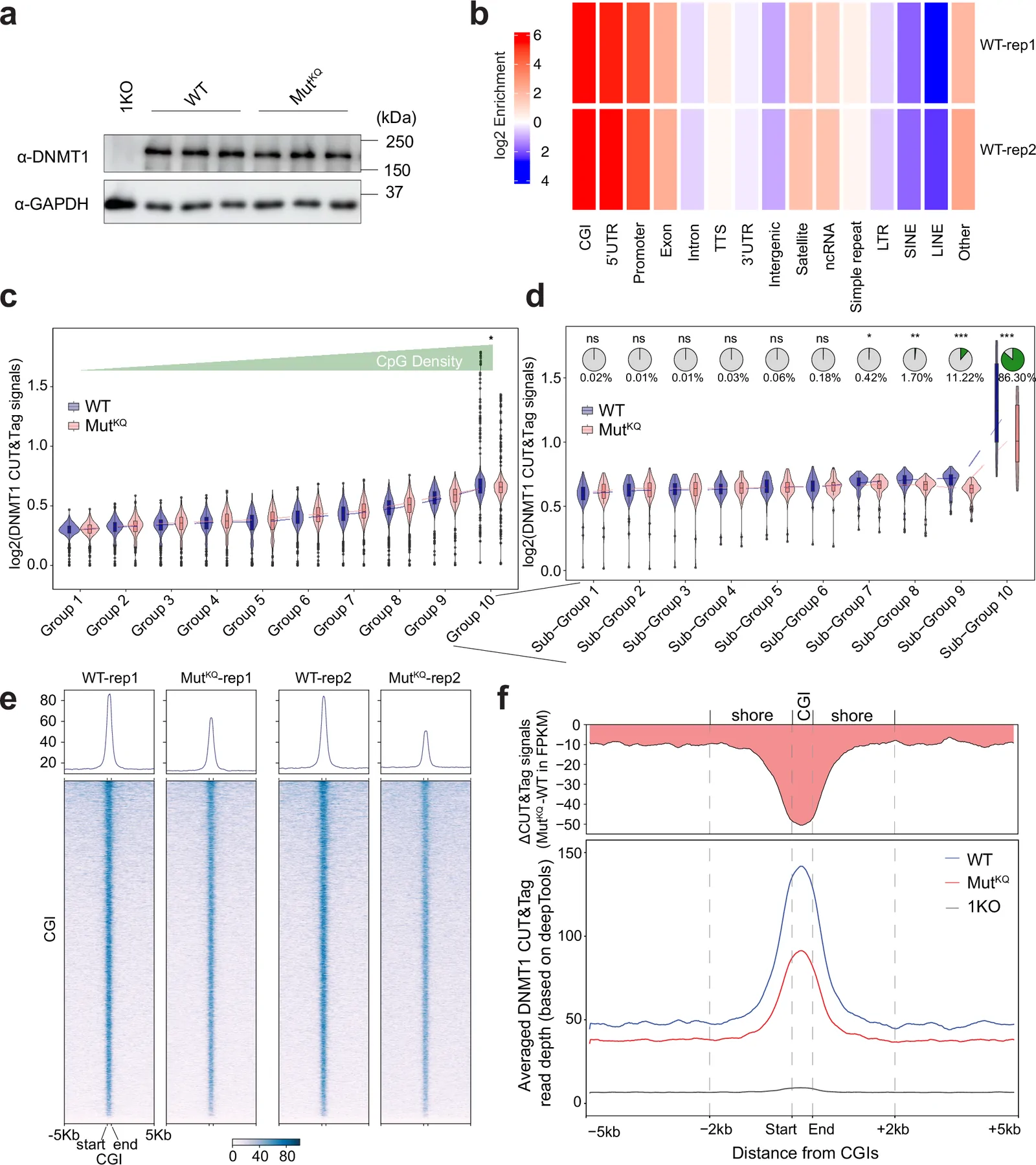
Unbiased genome-wide mapping unveils a preferential targeting of WT DNMT1 to genomic regions with high GC contents, notably CGIs, whereas the CXXC-CpG-interaction-defective mutant exhibits decreased binding to the corresponding regions. **a** Immunoblot for DNMT1 in the *Dnmt1*-knockout (1KO) mESCs transduced with 3×Flag-tagged exogenous DNMT1, either WT (lanes 2–4) or Mut^KQ^ (lanes 5−7). Parental 1KO-mESCs (lane 1) served as a negative control and anti-GAPDH blot (bottom) as a loading control. Western blot experiments were independently repeated 3 times with consistent results, and representative blots are shown. **b** Heatmap depicting the degree of enrichment of binding by WT DNMT1 to regions with the indicated genomic feature, as determined by HOMER. **c**, **d** Correlation between CpG density and binding of DNMT1, either WT or Mut^KQ^. Plotted at y-axis is the log2-converted value of DNMT1 CUT&Tag signals (read depth based on Reads Per Kilobase per Million mapped reads [RPKM] and after normalization to internal spike-in controls), across the non-overlapping 1-kb genomic windows (*n* = 2,647,190 total windows genome-wide, excluding those with ≥10% undetermined nucleotides). Windows were ranked by CpG density (**c**, x-axis) and divided into ten equally-sized decile groups (*n* = 500 bins per group, representing averaged signals from consecutive 530-kb bins; see “Methods” for details). The green triangle indicates the gradient from low to high CpG density. Notably, 99.9% of CGIs fall within Group 10 in (**c**). Group 10 bins were further subdivided into ten equally-sized subgroups to provide the closer views (**d**; *n* = 50 bins per subgroup), with the pie chart on the top displaying the proportion of all CGIs within each subgroup. Statistical significance was assessed using the two-sided Wilcoxon rank-sum test. ns, not significant; *, *P* < 0.05; **, *P* < 0.01; ***, *P* < 0.001. The boundaries of boxplots indicate the 25th and 75th percentiles, the center line indicates the median, and the whiskers indicate 1.5× the interquartile range. **e** Averaged plot and heatmap of CUT&Tag signals for DNMT1, either WT or Mut^KQ^. DNMT1 CUT&Tag signals (probed with anti-FLAG antibody) were normalized using the spike-in controls, spanning ±5 kb from CGIs. **f** Metagene plot comparing the WT versus Mut^KQ^ DNMT1 binding over a window of ±5 kb from CGIs, with bin size of 10 bp. The top panel plots the difference in binding (Δ%; Mut^KQ^-WT), while the bottom panel plots the binding levels of the 1KO, Mut^KQ^ and WT DNMT1 (y-axis shows read depth based on deepTools and after normalization to internal spike-in controls). Vertical dashed lines denote key genomic landmarks, namely, CGIs and CGI shores (defined to be ±2 kb from CGIs). CGIs coordinates were obtained from the mm10 genome assembly and extended by ±5 kb to include flanking regions. Each CGI, along with its flanking regions, was scaled to a uniform length (500 bp) to facilitate comparative analysis. Source data are provided with this paper.

DNMT1 is localized to the replication foci during the S phase but displays a diffuse nucleoplasmic pattern in non-S cell phases^14^. To delineate how the CXXC-CpG interaction affects DNMT1’s chromatin targeting and functionality in cells, we turned to the 1KO-mESCs and conducted rescue with either WT or the Mut^KQ^ version of 3×Flag-tagged DNMT1 (Fig. 3a), followed by Cleavage Under Targets and Tagmentation (CUT&Tag)^32^ for DNMT1 using Flag antibodies in independent cell samples. As a quality control, robust Flag-DNMT1 CUT&Tag signals were detected using 1KO-mESCs rescued with WT DNMT1, whereas little signals were observed in parental 1KO-mESCs (Supplementary Fig. 6). Notably, genomic binding of both WT DNMT1 and its Mut^KQ^ mutant were found to be most enriched in regions known to carry low levels of DNA methylation, notably, the CGIs and gene *cis*-regulatory elements (Fig. 3b, Supplementary Fig. 7a–c and Supplementary Data 1). For example, around 40% of the called peaks of WT DNMT1 are overlapped with CGIs (Supplementary Fig. 7a) and consistently, CGIs and the genomic regions known to be CGI-rich—such as gene promoters and adjacent 5’-UTRs—exhibit the most enrichment for DNMT1 binding at a genome-wide scale (Supplementary Fig. 7b, left panels and Supplementary Data 1). The Mut^KQ^ mutation did not apparently alter general patterns of DNMT1’s chromatin targeting or interaction with UHRF1 (Supplementary Fig. 7a–e and Supplementary Data 1). However, critical changes do exist following introduction of the Mut^KQ^ mutation versus WT controls. Despite the fact that both WT and Mut^KQ^ of DNMT1 are most enriched at the same GC-rich regions, the Mut^KQ^ mutant shows a significantly reduced binding to regions exhibiting the highest CpG density (Fig. 3c, d, see group 10 and sub-groups 7–10), accompanied by slight redistribution to regions with low CpG density and heterochromatic regions such as repetitive and transposable elements (Supplementary Fig. 7f). Furthermore, we specifically focused on the annotated CGIs across the genome and verified a significant reduction in the overall binding by Mut^KQ^ to CGIs when compared with WT DNMT1 (Fig. 3e, f).

Together, our unbiased profiling uncovers a positive correlation between DNMT1 binding and CpG density genome-wide, with its binding notably enriched at CGIs and classic CGI-rich regions (such as gene promoters and 5’-UTRs), reminiscent of what was previously reported for DNMT3A^33^. Additionally, these genomics results agree with the in vitro observations and lend strong support for the notion that DNMT1’s CXXC domain contacts CpG to potentiate DNMT1’s enrichment at CGIs.

### Genome-wide profiling reveals a role of CXXC-CpG interaction in tuning down DNMT1’s activity at genomic sites with high-GC content

**Fig. 4:**
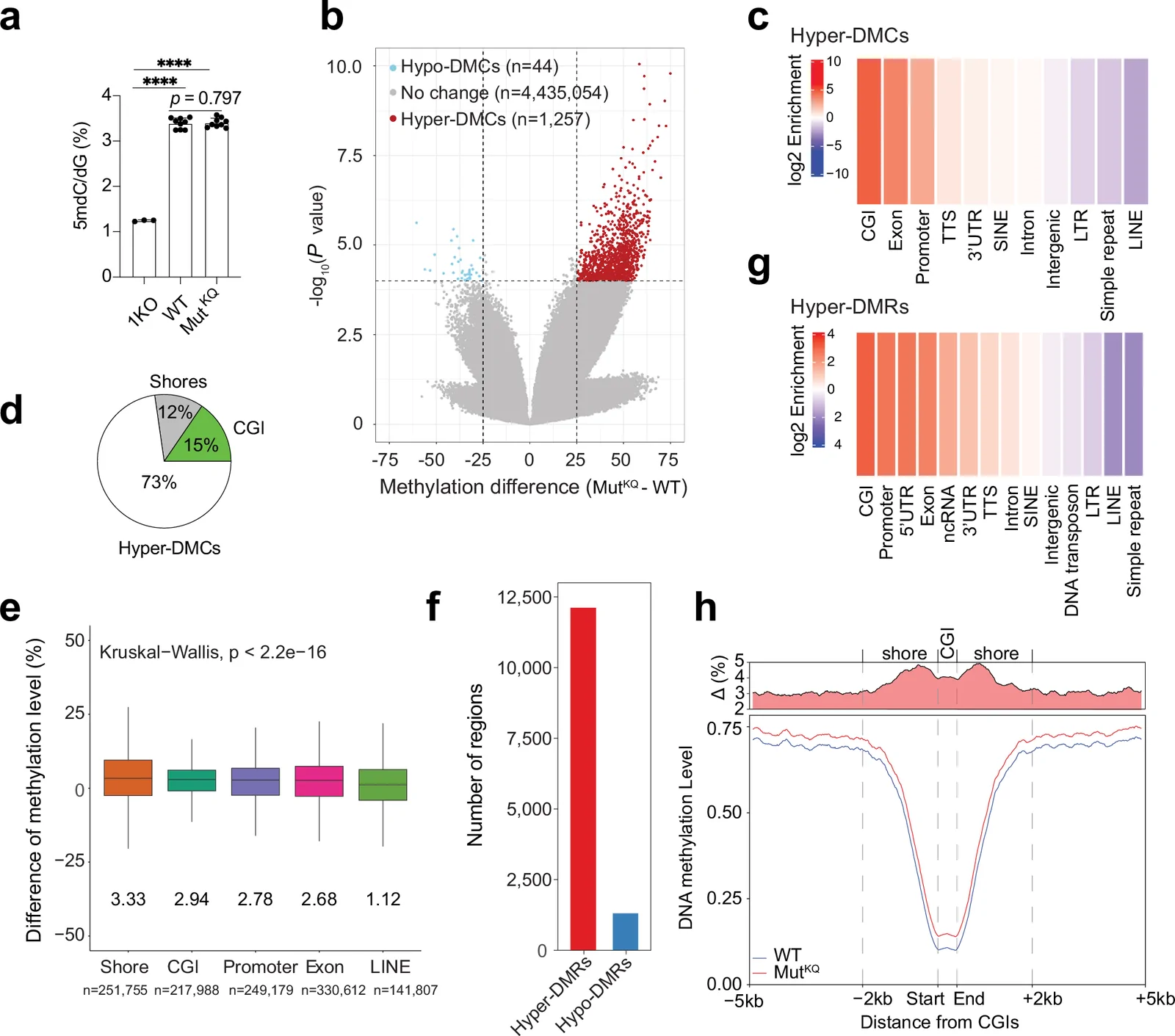
The CXXC-CpG interaction inhibits DNMT1 to safeguard the CGI hypomethylation. **a** LC-MS/MS analysis of global 5mC levels in parental *Dnmt1* knockout mESCs (1KO-ESCs; *n* = 3 technical replicates) and those reconstituted with wild-type (WT) *Dnmt1* or the Mut^KQ^ mutant of *Dnmt1* (*n* = 9 total measurements per group, derived from *n* = 3 independent biological replicates with 3 technical replicates each). A two-sided unpaired *t*-test was used for statistical analysis. ****, *P* < 0.0001. Data shown on y-axis are mean ± s.d. **b** Volcano plot illustrates the differentially methylated CpG sites (DMCs) in 1KO-ESCs with the Mut^KQ^ mutant verus WT DNMT1. A calling of DMCs was based on a cutoff of *P* less than 0.0001 and methylation difference greater than ≥25%, with hypermethylated and hypomethylated DMCs shown in red and cyan, respectively. **c** Heatmaps show the enrichment of hyper-DMCs across different indicated genomic annotation features. The enrichment score is calculated as log_2_ converted value of the observed versus expected frequency. Features with DMCs over 10 are displayed. **d** Pie chart showing the proportion of hyper-DMCs within CGIs, CGI shores, and other genomic regions. **e** Boxplots showing the pairwise, site-by-site difference in DNA methylation beta values (Δβ = β_MutKQ_ − β_WT_) for individual CpG sites across the indicated genomic annotation features (x-axis; e.g., CGIs, CGI shores). Statistical significance was confirmed by Kruskal-Wallis test (*P* < 2.2 × 10^−16^) and post-hoc pairwise comparisons (*P* < 0.0001). Sample sizes (number of CpG sites) are indicated below the x-axis. The boundaries of boxplots indicate the 25th and 75th percentiles, the center line indicates the median, and the whiskers indicate 1.5× the interquartile range. **f** The number of hyper-DMRs and hypo-DMRs in mESCs with the Mut^KQ^ versus WT DNMT1. The calling of DMR use the DSS script and a cutoff of q less than 0.01 and methylation difference greater than 15% for CpG sites with 10 or more coverages. **g** Heatmaps show the enrichment of differentially methylated regions (DMRs) exhibiting increased methylation (termed hyper-DMRs) across different indicated genomic annotation features. The enrichment score is calculated as log_2_ converted value of the observed versus expected frequency. Features with DMRs over 50 are displayed. **h** Metagene plot comparing the overall DNA methylation levels over CGIs-containing regions (±5 kb from the start and end of each CGI) between mESCs with the Mut^KQ^ versus WT DNMT1. The y-axis of bottom panel shows the averaged methylation level of the indicated sample, with the bin size at x-axis set to 10 bp for calculation, whereas the top panel shows the DNA methylation difference (Δ: Mut^KQ^ minus WT). CGIs coordinates were obtained from the mm10 genome assembly and extended by ±5 kb to include flanking regions. Each CGI, along with its flanking regions, was scaled to a uniform length (500 bp), shown in the center of plot, to facilitate comparative analysis. Vertical dashed lines denote CGIs and CGIs-shore (defined to be ±2 kb from CGIs). Source data are provided with this paper.

Next, we aimed to assess impact of the DNMT1 CXXC-CpG interaction on DNA methylation in cells. Towards this end, we first conducted the liquid chromatography-tandem mass spectrometry (LC-MS/MS) analysis using genomic DNA from the 1KO-mESCs rescued with DNMT1. Here, we detected a similar increase in global 5mC levels following the introduction of WT or the Mut^KQ^ mutant of DNMT1 into 1KO-mESCs (Fig. 4a). To further dissect putative focal changes of 5mC in the genome, we carried out whole-genome bisulfite sequencing (WGBS) using the same genomic samples. WGBS was successful—it achieved a nearly complete bisulfite conversion (with a rate of 99.5%–99.8% across all samples) and an average 17-fold coverage of the genome across all samples (approximately 400 million of total reads each sample) and also exhibited a low level of read tag duplication, allowing a high rate of read mapping to the reference genome (Supplementary Fig. 8a, b and Supplementary Data 2). First, pairwise comparison of WGBS data of duplicated samples for each group (such as replicates of WT or Mut^KQ^) revealed a high correlation and, additionally, WGBS profiles of cells with WT DNMT1 and the Mut^KQ^ mutant are largely similar across the genome (Supplementary Fig. 8c). Indeed, comparison of the global 5mC levels based on WGBS datasets detected no significant changes in 1KO-mESCs reconstituted with the Mut^KQ^ mutant versus WT DNMT1 (Supplementary Fig. 8d), consistent with the LC-MS/MS analysis (Fig. 4a). Next, we used the WGBS data to define the differentially methylated CpG sites (DMCs) in cells with the Mut^KQ^ mutant when compared to WT controls. Using either a stringent calling standard (Fig. 4b, with adjusted *P* value less than 1 × 10^–4^ and difference in methylation levels greater than 25%) or a less stringent one (Supplementary Fig. 8e, with adjusted *P* value less than 1 ×10^–3^ and difference in methylation levels greater than 15%), we defined DMCs and consistently observed hypermethylation to be associated with Mut^KQ^ relative to WT DNMT1 controls. Take DMCs based on the stringent calling for example—a vast majority (96.6%) of DMCs exhibited the increased cytosine methylation (termed as hyper-DMCs) whereas merely 44 sites (3.4%) showed the decreased methylation (termed as hypo-DMCs) (Fig. 4b). Genomic annotation showed these hyper-DMCs to be most enriched at CGIs and other regions exhibiting high-GC content (Fig. 4c and Supplementary Data 3), with approximately 27% of hyper-DMCs located at CGIs and CGI-adjacent regions (Fig. 4d). Additionally, calculation for methylation level differences in cells with the Mut^KQ^ versus WT DNMT1 revealed rather modest increases of 5mC across the genome, with the most obvious changes occurring at CGI shores (with CpG site-by-site methylation difference—or delta value—of 3.33%) and CGIs (with a delta value of 2.94%) (Fig. 4e, Supplementary Fig. 9a and Supplementary Data 4). Furthermore, we defined differentially methylated regions (DMRs) and observed that Mut^KQ^-associated hypermethylated DMRs (termed as hyper-DMRs) not only account for a vast majority of all DMRs (Fig. 4f) but are also most enriched for CGIs and CGI-rich regions such as promoters and 5’-UTRs (Fig. 4g and Supplementary Data 5). Focusing on CGIs, we observed that cells expressing the Mut^KQ^ mutant clearly exhibited significantly higher methylation levels in these regions when compared with WT controls (Fig. 4h). Taken together, our genome-wide profiling demonstrated that DNMT1’s CXXC domain interacts with CpG to selectively restrict DNMT1’s activity at genomic sites exhibiting high-GC contents, notably, CGIs and genomic regions known to be CGI-rich such as promoters.

### The DNMT1 CXXC domain operates to ensure CGI hypomethylation at developmentally crucial genes, potentiating appropriate cell lineage specification

**Fig. 5:**
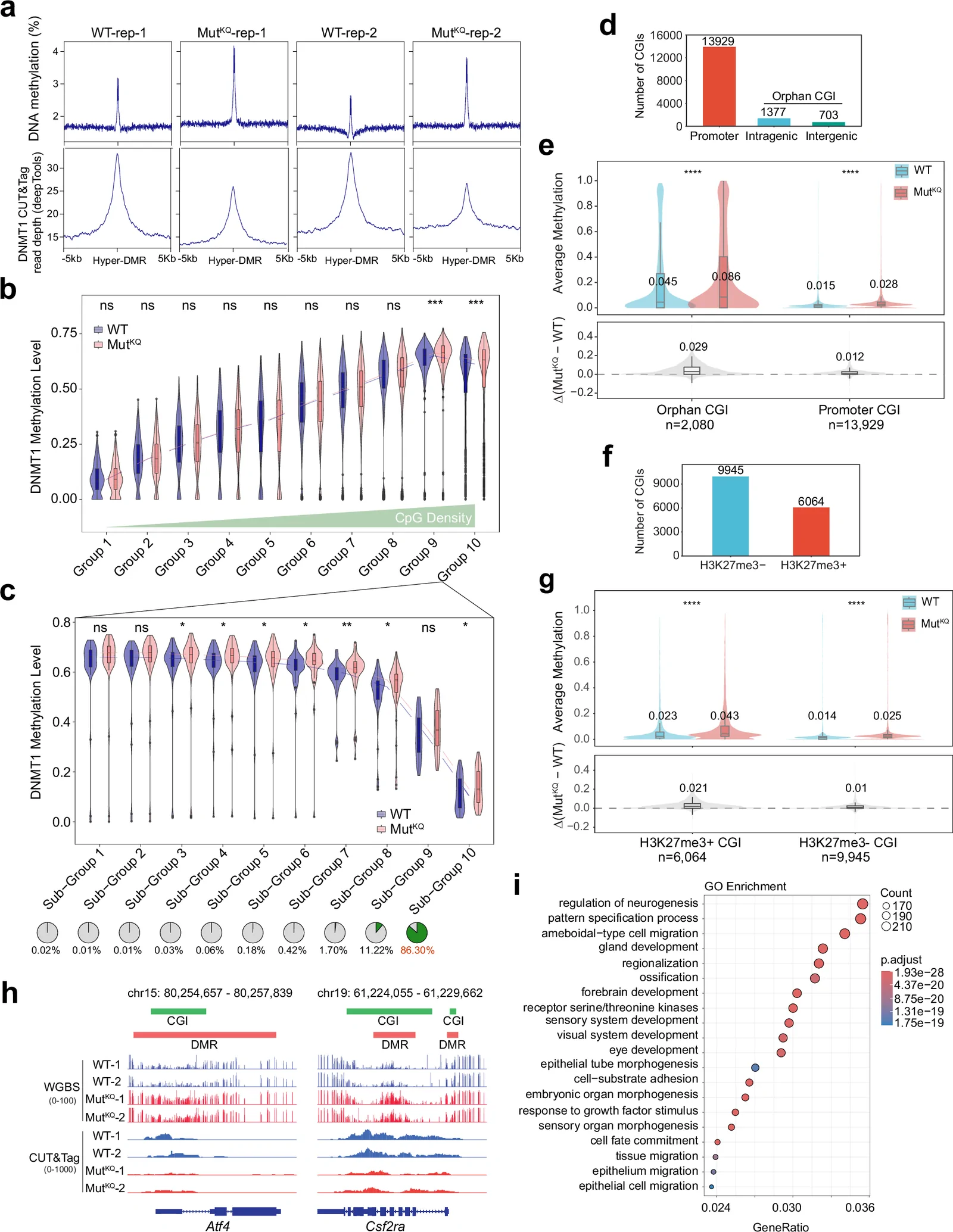
Genomic mapping of the DNMT1 Mut^KQ^-induced DNA hypermethylation. **a** Averaged DNA methylation levels (top; based on WGBS) and DNMT1 binding (bottom; based on CUT&Tag read depth, calculated based on deepTools and after spike-in control normalization) at hyper-DMRs detected in mESCs with the Mut^KQ^ versus WT DNMT1, visualized using deepTools. **b**, **c** Correlation between CpG density and methylation levels in cells with DNMT1, either WT or Mut^KQ^. Methylation level in (**b**) is plotted in non-overlapping 1-kb genomic windows (*n* = 2,647,190 total windows genome-wide, excluding those with ≥10% undetermined nucleotides). Windows were ranked by CpG density and divided into ten equally-sized decile groups (*n* = 500 bins per group, representing averaged methylation levels from consecutive 530-kb bins; see “Methods” for details). Notably, 99.9% of CGIs fall within Group 10 in (**b**). Group 10 windows in (**b**) were further subdivided into ten equal subgroups (*n* = 50 bins per subgroup) in (**c**) to provide finer resolution, with pie chart showing the proportion of all CGIs within each subgroup. Statistical significance for groups 1–10 and subgroups 1–9 was assessed using the two-sided Wilcoxon rank-sum test, while that for subgroup 10 was assessed using a permutation test due to its mixture properties. ns, not significant; *, *P* < 0.05; **, *P* < 0.01; ***, *P* < 0.001. The boundaries of boxplots indicate the 25th and 75th percentiles, the center line indicates the median, and the whiskers indicate 1.5× the interquartile range. **d** Bar chart displaying the distribution and absolute counts of annotated CpG islands (CGIs) categorized as either Promoter CGIs (TSS ± 2 kb) or Orphan CGIs (subdivided into intragenic and intergenic elements). **e** Top panel: Violin plots illustrating average DNA methylation levels in WT (blue) and CXXC-deficient Mut^KQ^ (red) cells across Orphan vs. Promoter CGIs. Numbers represent population medians. Bottom panel: Expanded distribution of the direct methylation shift (Delta = Mut^KQ^ - WT) per individual CGI locus, overlaid with central boxplots. Total island counts (*n*) are indicated beneath all functional groups. Statistical significance was evaluated using a two-tailed paired Wilcoxon signed-rank test (****P < 0.0001). **f** Distribution and absolute counts of total CGIs stratified by Polycomb chromatin status into H3K27me3- (active/unmarked) and H3K27me3+ (PcG-repressed) cohorts. **g** Top panel: Violin plots comparing baseline and mutant fractional methylation distributions between H3K27me3+ and H3K27me3- CGIs. Bottom panel: Direct locus-by-locus methylation variance (Delta = Mut^KQ^ − WT). Total island counts (*n*) are indicated beneath all functional groups. Statistical significance was evaluated using a two-tailed paired Wilcoxon signed-rank test (****P < 0.0001). **h** Integrative Genomic Viewer (IGV) tracks showing DNA methylation (WGBS) and DNMT1 binding (CUT&Tag) at the *Atf4* and *Csf2ra* gene locus in 1KO-mESCs with WT DNMT1 or the Mut^KQ^ mutant (*n* = 2 replicated samples). **i** Bubble plot illustrating the gene ontology (GO) term enrichment for genes associated with hyper-DMRs in mESCs with the Mut^KQ^ versus WT DNMT1. The size of each dot indicates the count of genes enriched in the pathway, and the color gradient represents the Benjamini-Hochberg adjusted *P* value (*p*.adjust, derived from a one-sided hypergeometric test with multiple testing correction). Source data are provided with this paper.

Next, we integrated the WGBS and DNMT1 CUT&Tag profiles to further dissect the role of DNMT1’s CXXC interaction with CpG in cells. First and at the Mut^KQ^-associated hypermethylated DMRs, we consistently observed a decrease in binding of the Mut^KQ^ mutant, relative to WT controls, in independent samples (Fig. 5a). Next and as shown in Fig. 3c, d, we binned the whole genome into groups based on the degree of CpG density. Here, only in those regions showing the highest CpG density did we detect significantly increased methylation in cells with the Mut^KQ^ versus WT DNMT1 (Fig. 5b, c, groups 9 and 10 and sub-groups of the group 10), concomitant with the decreased DNMT1 binding by the Mut^KQ^ mutation (Fig. 3c, d). Such changes are obvious at orphan and H3K27me3-positive CGIs that are associated with developmentally crucial genes, such as *Atf4* (a transcription factor known to regulate osteoblast differentiation^34^), *Gata6* (a crucial transcription factor regulating endodermal lineage specification^35^), *Neurog1* (a gene that specifies neuronal lineage), and *Csf2ra* (a cytokine receptor involved in myeloid cell differentiation^36^) (Fig. 5d–h and Supplementary Fig. 9b). In fact, gene annotation of those Mut^KQ^-associated hyper-DMRs revealed significant enrichment for gene sets involved in early development, such as neurogenesis, pattern specification, and cell fate commitment (Fig. 5i and Supplementary Fig. 9c). Along the line, we profiled the H3K27me3 signal in WT and Mut^KQ^ cells by CUT&Tag and observed genome-wide decrease of H3K27me3 at CGIs in the latter cells, with the significant change occurring at the promotor-overlapping CGIs (TSS ± 2 kb) of the developmental genes (Supplementary Fig. 10a–d). These data suggest that disruption of the DNMT1 CXXC-CpG interaction leads to a shift from H3K27me3 mark to DNA methylation at the polycomb targets, reflecting a change in local chromatin regulation. The detailed mechanism underlying this shift merits investigation in future.

**Fig. 6:**
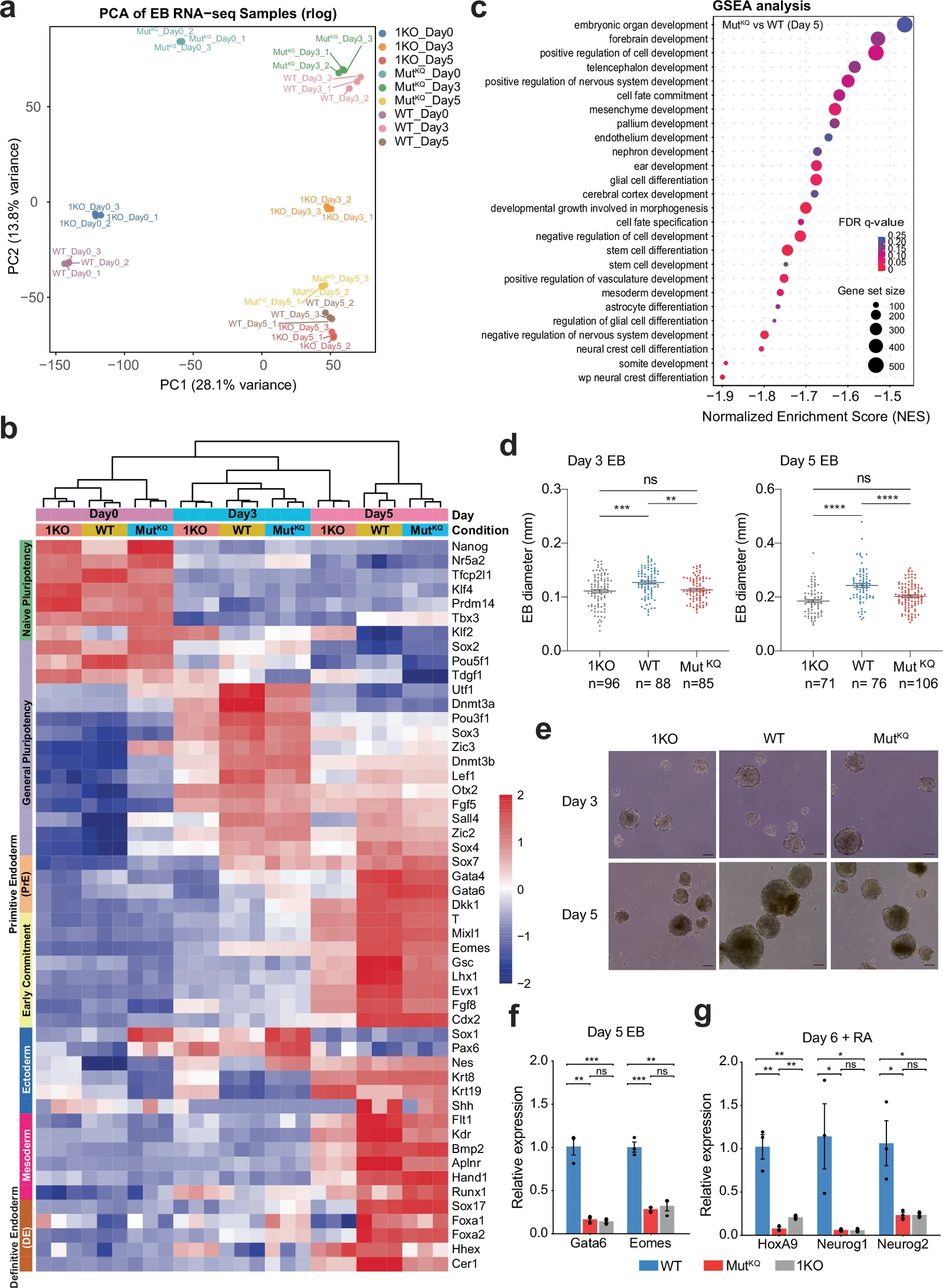
The DNMT1 CXXC domain is essential for appropriate differentiation of mESCs. **a** Principal Component Analysis (PCA) of RNA-seq samples (rlog-transformed counts) spanning a differentiation time course at day 0, day 3, and day 5 following embryoid body (EB) formation using 1KO-mESCs or 1KO-mESCs reconstituted with WT DNMT1 or the Mut^KQ^ mutant. Replicates (*n* = 3) cluster tightly by time point and genotype. **b** Heatmap comparing lineage specification and pluripotency gene marker expression across the WT-DNMT1, Mut^KQ^ and 1KO samples across all time points. Left annotation color bars delineate functional developmental categories: Naive Pluripotency, General Pluripotency, Primitive Endoderm (PrE), Early Commitment, Ectoderm, Mesoderm, and Definitive Endoderm (DE). Selected markers were compiled and referenced based on established differentiation frameworks^94–97^. Color scales denote relative expression levels (z-score of normalized counts). **c** Gene Set Enrichment Analysis (GSEA) profile. Pre-ranked gene lists were generated by scoring total transcripts via signed significance ranking (sign(log_2_FoldChange) × −log_10_(*p*-value)), capped at a maximum value of 300 to break ties. Normalized Enrichment Scores (NES) are plotted against Molecular Signatures Database (MSigDB) annotated pathways, filtered for development- and differentiation-associated terms under an FDR *q*-value cutoff of <0.25. **d**, **e** Quantification of EB size (**d**; *n*, the number of EBs) and representative images of EBs (**e**) using parental 1KO-mESCs or those reconstituted with WT DNMT1 or the Mut^KQ^ mutant, at day 3 and 5 following withdrawal of LIF and feeder cells. Statistical significance was determined using one-way ANOVA in GraphPad Prism. Data in (**d**) are presented as mean ± s.e.m. Scale bar in (**e**), 100 µm. **f** RT–qPCR of the indicated cell lineage marker gene in EBs formed by mESCs with WT DNMT1, the Mut^KQ^ mutant or 1KO, at day 5 post-differentiation. Data represent mean ± s.e.m. from three independent experiments. *P* values were determined by two-tailed Student’s *t*-test. **g** RT–qPCR of the indicated neuronal lineage marker gene, at day 6 following retinoic acid (RA)-induced differentiation of mESCs with WT DNMT1, the Mut^KQ^ mutant or 1KO. Data represent mean ± s.e.m. from three independent experiments. *P* values were determined by two-tailed Student’s *t*-test. ns, not significant; *, *P* < 0.05; **, *P* < 0.01; ***, *P* < 0.001. Source data are provided with this paper.

Next, we investigated the requirement of the DNMT1 CXXC-CpG interaction for stem cell differentiation. To capture the dynamic gene expression changes during early lineage commitment, we performed a time-course RNA-seq analysis comparing WT and Mut^KQ^ cells at different stages of embryoid body (EB) formation (day 0, day 3, and day 5). Principal Component Analysis (PCA) confirmed high reproducibility and tight clustering among all biological replicates, providing a robust foundation for our differential expression analysis (Fig. 6a). Our analysis revealed that while WT cells follow a standard, highly coordinated and expected differentiation cascade (Supplementary Fig. 11), this program was significantly impaired in both Mut^KQ^ and 1KO lines (Fig. 6b). We observed a marked failure to activate key lineage-specific marker genes. Of note, in the Mut^KQ^ and 1KO cells relative to WT controls, several pluripotency and early specification genes expected to increase during the early transition (e.g., *Oct4/Pou5f1*, *Utf1*, *Dnmt3a*, *Pou3f1*, *Sox3*, *Lef1*, *Otx2* and *Sall4*) failed to reach expected expression levels by day 3 in the EB formation process (Fig. 6b); additionally, a broader suite of differentiation markers for later stages (e.g., *Gata4*, *Gata6*, *Dkk1*, *T*, *Mixl1*, *Eomes*, *Gsc*, *Lhx1*, *Evx1*, *Fgf8*, *Shh*, *Flt1*, *Kdr*, *Aplnr*, *Foxa1*, *Foxa2*, *Hhex* and *Cer1*) showed significantly lower expression in the Mut^KQ^ and 1KO cells compared to WT cells at day 5 (Fig. 6b).

Consistently, Gene Set Enrichment Analysis (GSEA) using MSigDB demonstrated that pathways fundamentally required for embryogenesis, axis specification, and lineage commitment were systematically downregulated or disrupted in the Mut^KQ^ cells at day 5 (e.g., mesoderm formation, mesoderm morphogenesis, mesoderm development, mesenchyme development, stem cell differentiation, embryonic organ morphogenesis, cell fate commitment, somite development, etc.) (Fig. 6c and Supplementary Fig. 12a). These pathways were similarly identified by Gene Ontology (GO) analysis (Supplementary Fig. 12b).

Prompted by these broad transcriptomic deficits, we next evaluated whether the disrupted gene expression program manifested in overt cellular and differentiation phenotypes. In the three-dimensional (3D) EB formation assay^37^, defects were observed using 1KO-mESCs re-expressing Mut^KQ^ when compared with the WT DNMT1-expressing controls—upon induction of differentiation, EBs generated by the former were markedly smaller than their WT counterparts (Fig. 6d, e). Quantitative polymerase chain reaction following reverse transcription (RT–qPCR) further demonstrated that, compared with EBs derived from mESCs with WT DNMT1, the counterparts expressing the Mut^KQ^ mutant exhibited perturbed induction of lineage-specific marker genes such as *Gata6* and *Eomes* (Fig. 6f), which are master regulators specifying the mesoderm and/or endoderm fates. Meanwhile, EBs generated by mESCs with Mut^KQ^ relative to WT controls showed persistent expression of pluripotency genes such as *Sox2* and *Oct4/Pou5f1* (Supplementary Fig. 12c), consistent with the observed defect and delay in EB formation. Lastly, we employed a separate monolayer-based neuronal differentiation protocol and observed similar differentiation-related defects—upon the retinoic acid (RA)-mediated induction of neuronal cell differentiation, cells derived from mESCs expressing Mut^KQ^ showed the reduced expression of neuronal lineage markers (such as *Neurog1*, *Neurog2*, and *Hoxa9*), when compared with their counterparts expressing WT DNMT1 (Fig. 6g and Supplementary Fig. 12d).

Altogether, we show that DNMT1’s CXXC domain directly engages CpG to suppress DNA methylation at those CGIs associated with developmental genes, a process critical for proper stem cell differentiation.

## Discussion

**Fig. 7:**
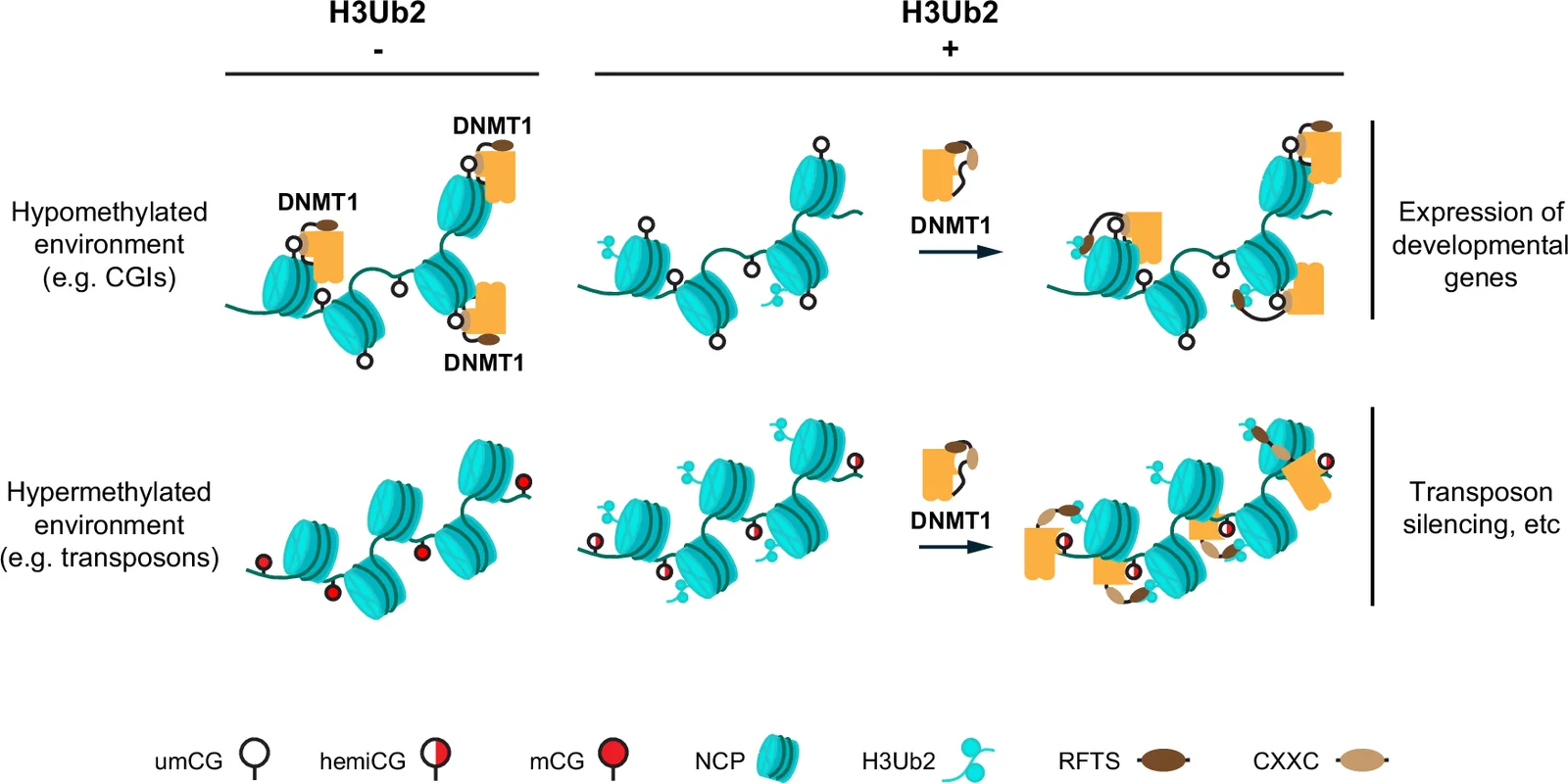
A regulatory model for DNMT1-mediated DNA methylation in different chromatin environments. Under the regulation of H3 ubiquitylation (H3Ub2 + ), the recruitment and DNA methylation activity of DNMT1 is boosted at hypermethylated environment, but not as much as at hypomethylated environment due to limited UHRF1 activity as well as the CXXC domain-mediated autoinhibition. In the absence of H3Ub2 regulation (H3Ub2-), the autoinhibitory DNMT1 is relatively enriched in CGIs, which is in part attributed to the CXXC-CpG interaction. umCG, unmethylated CpG sites; hemiCG, hemimethylated CpG sites; mCG, fully methylated CpG sites.

The interplays between the chromatin environment and epigenetic factors play crucial roles in regulating diverse gene activities across the eukaryotic genome^38^. In mammals, dynamic DNA methylation and nucleosome landscapes help partition the chromatin into functionally distinct domains, such as transposons, exons and CGIs^39^. Yet, how DNMTs interact with nucleosomes under diverse cellular contexts to orchestrate DNA methylation homeostasis remains poorly understood. This study addresses this question through comprehensive structure-function characterization of the interaction between DNMT1 and the CpG site in a hypomethylated nucleosome environment. On one hand, our unbiased genome-wide profiling of DNMT1 by CUT&Tag demonstrated that, in mESCs, DNMT1 is preferentially targeted to those genomic regions typically known to be unmethylated or hypo-methylated, notably CGIs and CGI-rich elements. On the other hand, the interaction of the DNMT1 CXXC domain with the CpG site serves to restrict DNMT1’s activity in the corresponding regions, disruption of which led to DNA hypermethylation but H3K27me3 decrease at the promoter-overlapping CGIs of the developmental genes, reflecting a shift in local chromatin regulation between the two competing epigenetic mechanisms^40–43^. In line with such epigenetic dysregulation, genes involved in embryogenesis, axis specification, and lineage commitment were systematically downregulated or disrupted in EB formation, leading to severe defects in cell differentiation. Together, these observations support a mechanism in which the DNMT1 CXXC domain senses the hypomethylated environment of CGIs to safeguard the proper DNA methylation landscape critical for cell lineage commitment (Fig. 7). Note that this notion is supported by a recent study (published while this paper is under revision), which showed that DNMT1 is enriched in CGIs under steady-state conditions in MV-4-11 cells^44^. The exact mechanism behind the enrichment of DNMT1 in CGIs remains unclear. Conceivably, stationing the autoinhibitory DNMT1 molecules in a non-target chromatin domain may help protect local CpG sites from being targeted by de novo DNA methyltransferases DNMT3A and DNMT3B, or those active-state DNMT1 molecules, while permitting rapid recruitment of DNMT1 molecules to endogenous target regions during the phase of maintenance DNA methylation.

The 3–40-fold substrate preference of DNMT1 for hemimethylated DNA substrates is knowingly insufficient for precise maintenance of the per-site DNA methylation patterns^6,11,45^. In circumventing this limitation, multiple factors, which at least include UHRF1^7,8^ and repressive histone modifications H3K9me3 and H4K20me3^22,23^, provide critical regulatory mechanisms to ensure proper deposition of methylation by DNMT1 under various chromatin contexts. For instance, it has been shown that the interaction of UHRF1 with H3K9me3 and hemimethylated CpG DNA promotes both the chromatin association and E3 ubiquitin ligase activity of UHRF1, which in turn recruits DNMT1 to heterochromatic regions, such as transposable elements, for DNA methylation maintenance^46–48^ (Fig. 7). On the other hand, the lack of H3K9me3 in the transcriptionally active CGIs limits the chromatin association and ubiquitylation activity of UHRF1, leading to downregulation of DNMT1-mediated maintenance DNA methylation in the corresponding regions^10,11^ (Fig. 7). Through structure-function characterization of DNMT1 bound to a hypomethylated NCP, this study demonstrates that even after the RFTS-mediated autoinhibition is relieved by the RFTS-H3Ub2 (the enzymatic product of UHRF1 made during replication) interaction, DNMT1 remains subject to autoinhibitory regulation by the CXXC-CpG interaction, which is important for restricting DNMT1’s activity in CGIs. All in all, this study provides a mechanism that the hypomethylated chromatin environment directly tunes down the DNA methylation activity of DNMT1, adding a critical layer onto the previously characterized UHRF1-H3Ub2 axis for a multilayered regulation of DNMT1-mediated DNA methylation (Fig. 7).

DNA hypo-methylation is a hallmark of the CGIs and CGI-overlapping promoters in the vertebrate genomes, and epigenetic states of CGIs are critically involved in transcriptional regulation^49^. The CXXC domain, a reader of the unmodified CpG that coevolves with the CGIs^50^, is present in various chromatin modifiers and safeguards the chromatin state of CGIs through diverse mechanisms^51–60^. For instance, the CXXC domains of the mixed-lineage leukemia 1 and lysine demethylase 2B (KDM2B) proteins bind the unmodified CpG to protect the polycomb-bound region from DNA hypermethylation^52,61^, the CXXC domains of CPF1 protein binds to unmodified CpG for recruiting the Set1 methyltransferase to deposit the H3K4me3 marks in the active promoter regions^62^, and the CXXC domain of Tet3 protein binds to unmodified CpG or 5-carboxylcytosine in the CpG context to restrict the activity of Tet3 to transcription start sites^60,63^. Our current study shows that the CXXC domain of DNMT1 binds the unmethylated CpG to orchestrate an autoinhibitory state of DNMT1, which in turn inhibits DNA methylation in a hypomethylated environment to ensure proper stem cell differentiation. Therefore, this work provides an example that the CXXC domain in a repressive chromatin modifier helps maintain the hypomethylated state of the CGIs, which is critical for cell fate determination.

## Methods

### Plasmids

A cDNA of full-length human DNMT1, fused in-frame with an N-terminal 3×Flag tag by PCR, was inserted into the pPyCAGIP vector (a kind gift of I. Chambers) and then used for mammalian expression as described before^23^. For structural and biochemical characterization, the DNA fragment encoding full-length or the residues 351–1600 of human DNMT1 was cloned into a home-made bacterial expression vector containing an N-terminal His_6_-MBP tag. In addition, a C-terminal tryptophan was introduced to the CXXC domain for the purpose of spectroscopic observation of the domain alone. DNMT1 mutation (Mut^KQ^: K683A/Q684A, or Mut^MHKKKQ^: M718A/H719A/K723A/K724A/K725A/Q726A) was generated via site-directed mutagenesis, with the residue numeration based on the isoform 1 of DNMT1 that contains a total of 1616 amino acids. All plasmid sequences were verified by Sanger sequencing before use.

### Protein expression and purification

#### DNMT1 proteins

The plasmid for DNMT1_351–1600_ was transformed into *Escherichia coli* BL21 DE3 (RIL) cells for expression. The cells were initially grown in LB medium at 37 °C, and the temperature was lowered to 16 °C after the cell density reached OD_600_ (optical density at 600 nm) of 0.8. Subsequently, the cells were induced with 100 µM Isopropyl β-D-1 thiogalactopyranoside (IPTG) and continued to grow overnight. The cells were collected, lysed in lysis buffer (50 mM Tris-HCl, pH 8.0, 1 M NaCl, 25 mM imidazole, 1 mM PMSF) by Avestin Emulsiflex C3 homogenizer, and the insoluble cell debris were removed by centrifugation. The supernatant was loaded onto a Ni^2+^-NTA affinity column, washed with lysis buffer, and eluted with elution buffer (20 mM Tris-HCl, pH 8.0, 100 mM NaCl, 300 mM imidazole). Subsequently, the His_6_-MBP-tagged fusion protein was subjected to anion exchange chromatography on a HiTrap Q XL column (Cytiva), tag removal by Tobacco etch virus (TEV) protease, and a second-round nickel affinity chromatography. The DNMT1 proteins were finally purified through size-exclusion chromatography on a HiLoad 16/600 Superdex 200 pg column (GE Healthcare) pre-equilibrated with a buffer containing 20 mM Tris-HCl (pH 7.5), 150 mM NaCl, 5% glycerol and 5 mM DTT. The purified protein was stored in a –80 °C freezer for future use.

The DNMT1 CXXC domain was purified in the same manner as described above, except that after TEV cleavage, the tag-free CXXC domain was purified on a Heparin column (Cytiva), followed by size-exclusion chromatography on a HiLoad 16/600 Superdex 75 pg column (GE Healthcare) equilibrated with a buffer containing 20 mM Tris-HCl (pH 7.5), 150 mM NaCl, 5% glycerol and 5 mM DTT. The DNMT1 mutants were purified in the same approach as that for WT proteins.

#### *Xenopus* histones and H4K20me3 modification

Preparation of *Xenopus* histones follows a previously published protocol^64^ with slight modifications. In essence, wild-type and mutant histones were expressed in an insoluble form in *Escherichia coli* BL21 (DE3) RIL at 37 °C. The cells were harvested 1 h after induction by 0.4 mM IPTG. The cells were lysed in a buffer containing 50 mM Tris-HCl, pH 8.0, 0.2 M NaCl, 1% Triton X-100, and 1 mM PMSF. After centrifugation, the inclusion body was collected and washed with the buffer above and solubilized in solubilization buffer containing 7 M guanidine hydrochloride, 20 mM Tris-HCl (pH 7.5) and 10 mM DTT, followed by sonication. Subsequently, histones were purified through sequential ion-exchange chromatography on Q-XL and SP-HP columns (GE Healthcare), using a denaturation buffer containing 20 mM Tris-HCl, pH 7.5, 7 M urea and 2 mM β-mercaptoethanol, with a KCl gradient of 0 to 1 M. The purified histone fractions were dialyzed against distilled water containing 2 mM β-mercaptoethanol, then concentrated and stored at −80 °C. For preparation of histone H4 carrying a mimic of H4K20me3 modification (H4Kc20me3, denoted as H4K20me3 herein), histone H4 mutant H4K20C was alkylated as previously described^22^.

#### H3K18Ub/H3K23Ub (H3Ub2) modification

The G76C-mutated ubiquitin was expressed and purified as described previously^65^. In brief, the cDNA sequence encoding G76C-mutated ubiquitin was cloned into a modified pRSF-Duet vector preceded by a His_6_-SUMO tag and a ULP1 (Ubl-specific protease 1) cleavage site. The generated plasmid was transformed into *Escherichia coli* BL21 DE3 (RIL) cells for protein expression. After elution from the Ni^2+^-NTA column, the His_6_-Sumo-tagged ubiquitin G76C was mixed with K18C/K23C/C110S-mutated histone H3 in an 8:1 molar ratio and dialyzed against buffer containing 250 mM Tris-HCl, pH8.6, and 8 M urea at 4 °C overnight. The protein mixture was then supplemented with 5 mM TCEP and incubated at room temperature for 30 min. Next, the crosslinker 1,3-dichloroacetone was added into the solution with the amount equal to one half of the total sulfhydryl groups in the proteins. The reaction mixture was incubated on ice for 2 h and then quenched by 5 mM β-mercaptoethanol. The reaction product was purified through a SP-HP column (GE Healthcare) in the denaturation buffer with a KCl gradient of 0–1 M, followed size-exclusion chromatography on a HiLoad 16/600 Superdex 200 pg column (GE Healthcare) in a buffer containing 20 mM Tris-HCl (pH 7.5), 7 M urea, 1 M NaCl, and 2 mM β-mercaptoethanol. Subsequently, the His_6_-SUMO tag of the reaction product was removed by ULP1 cleavage. The tag-free, H3K18Ub/K23Ub-modified H3 protein was finally purified by nickel affinity chromatography. The purified histone protein was concentrated and stored at −80 °C before use.

### Preparation of nucleosome DNA

The nucleosome DNA for the DNMT1-^um^NCP complex (um-NucDNA), in which the 601 DNA in the um-NucDNA is preceded by a TpT dinucleotide on the 5′-end, was generated through PCR amplification. The product was then purified using a Mono Q 5/50 GL column (Cytiva).

To measure the steady-state kinetics of WT or mutant DNMT1_351–1600_, we generated the U-Linker1^5hmCG^-Linker2^CG^, U-Linker1^5hmCG^-Linker2^CA^, U-Linker1^3hmCG–6CG^ and U-Linker1^3hmCG–6CA^ nucleosome DNAs. The 601 DNA in the U-Linker1^5hmCG^-Linker2^CG^ is flanked by one 34-bp linker containing 5 hemiCG sites (Linker 1), and one 30-bp linker containing 6 CpG sites (Linker 2). The U-Linker1^5hmCG^-Linker2^CA^ DNA shares the same sequence as the U-Linker1^5hmCG^-Linker2^CG^, except that the CpG sites on Linker 2 were replaced by the CpA sites. The 601 DNA in the U-Linker1^3hmCG–6CG^ is flanked by one 34-bp linker containing 3 hemiCG sites, surrounded by 6 CpG sites, and one 30-bp linker lacking CpG or hemiCG sites. The U-Linker1^3hmCG–6CA^ shares the same sequence as U-Linker1^3hmCG–6CG^ except that the CpG sites flanking the hemiCG sites were replaced by CpA sites.

For EMSA assays, we generated 201-bp NucDNA^CG^ and 211-bp CG^Mut^-601 nucleosome DNA. The 601 DNA in the NucDNA^CG^ is flanked by one 34-bp linker containing 9 CpG sites and one 30-bp CpG-lacking linker. CG^Mut^-601 shares the same DNA sequence as U-Linker1^3hmCG–6CA^ except that 5 dyad-adjacent CpG sites were replaced by non-CpG sites. The NucDNA^CG^ DNA was also used in assembling nucleosome for de novo DNA methylation assay. In addition, U-Linker1^3hmCG–6CA^ was used as native 601 nucleosome DNA for the EMSA assay.

For the BLI binding assay, we generated the biotinylated nucleosome DNA (biotin-NucDNA) through PCR amplification. Two 25-bp linkers, one of which was modified with 5′-biotin, were incorporated.

The primers and sequences of the nucleosomal DNAs used in this study are summarized in Supplementary Data 6 and 7, respectively.

### Reconstitution of nucleosomes

Reconstitution of histone octamer and nucleosomes were performed as described previously^65^. In essence, equimolar purified histone H2A-H2B tetramer, H3 and H4 were mixed and dialyzed sequentially against denaturation buffer in the absence of β-mercaptoethanol, a buffer containing 20 mM Tris-HCl, pH 7.5, 7 M Guanidine-HCl and 10 mM DTT, and a buffer containing 20 mM Tris-HCl, pH 7.5, 2 M NaCl, 1 mM EDTA and 5 mM β- mercaptoethanol at 4 °C. The histone octamer was then concentrated and purified using a HiLoad 16/600 Superdex-200 pg column (GE Healthcare). The purified histone octamer sample was concentrated and stored at –80 °C before use. The nucleosomes were assembled with the purified histone octamer and DNA in 1:1.1 molar ratio and reconstituted by stepwise salt dialysis against a buffer containing 10 mM Tris-HCl (pH 7.5), 1 mM EDTA, and 1 mM DTT in a gradient of 0.25–2 M KCl. After 24 h of dialysis, the assembled NCP was dialyzed against another buffer containing 10 mM Tris-HCl (pH 7.5) and 1 mM DTT for 3 h. The reconstituted nucleosomes were stored at 4 °C before use.

### In vitro DNA methylation assay

All in vitro DNA methylation assays were performed in 20-µL reactions containing indicated concentrations of WT or mutant DNMT1 protein and nucleosome in a buffer containing 0.56 µM *S*-adenosyl-L-[methyl-^3^H] methionine with a specific activity of 18 Ci/mmol (PerkinElmer), 50 mM Tris-HCl (pH 8.0), 100 mM NaCl, 0.05% β-mercaptoethanol, 5% glycerol and 200 µg/mL BSA. The reaction mixture was incubated at 37 °C for various times, before being quenched by 5 µL of 10 mM nonradioactive *S*-adenosyl-L-methionine (SAM) (Sigma). Subsequently, eight μL of the reaction mixture was loaded onto Amersham Hybond-N+ nylon membrane (Cytiva) and left air dried. The membrane was then washed twice in 0.2 M ammonium bicarbonate (pH 8.2) for 30 min, once in deionized water for 30 min, and once with 95% ethanol for 5 min. Finally, the membrane for each reaction was soaked in 4 mL of scintillation cocktail (Fisher) and the ^3^H radioactivity measured by a Beckman LS6500 counter. Each reaction was repeated three times.

#### De novo DNA methylation assay for WT DNMT1_351–1600_ vs Mut^KQ^

Each reaction contains 10 nM WT DNMT1_351–1600_ and 200 nM NucDNA^CG^ nucleosome, dissolved in the reaction buffer. The reaction mixtures were incubated for 0, 30, and 60 min for kinetic analysis.

#### Steady-state kinetics of WT or mutant DNMT1_351–1600_

Each reaction contains 10 nM DNMT1_351–1600_ protein and nucleosomes at various concentrations (10, 20, 40, 80, 120, 160, and 200 nM), dissolved in the reaction buffer supplemented with 0.65 µM cold SAM. The initial DNA methylation rate was determined by measuring the DNA methylation of each reaction mixture at 0 and 60-min time points. Linear progression of DNA methylation within this duration was confirmed through kinetic analysis of the reactions containing 10 nM DNMT1_351–1600_ protein and 200 nM nucleosome.

### Electrophoretic mobility shift assay

NucDNA^CG^, U-Linker1^3hmCG–6CA^ and CG^mut^-601 nucleosomes were used to compare the DNA- or nucleosome-binding affinity of WT and/or Mut^KQ^ mutant DNMT1. In essence, 0.1 µM partially dissociated nucleosome was incubated with various concentrations of DNMT1 in a buffer containing 10 mM Tris-HCl (pH 7.5), 100 mM (for NucDNA^CG^) or 50 mM (for U-Linker1^3hmCG–6CA^ and CG^mut^-601) NaCl, 10% Glycerol, and 1 mM DTT. The sample mixtures were loaded in a 5% DNA retardation gel (Invitrogen) and electrophoresed in 0.25x TBE buffer at 4 °C under 100 V for 90 min. The bands for free and protein-bound nucleosomes were visualized by ChemiDoc Imaging System (Bio-Rad).

### Biolayer interferometry (BLI) assay

The interactions between DNMT1_351–1600_, WT or Mut^KQ^, and the 5′-biotin-labeled nucleosome, containing either unmodified or H3Ub2/H4K20me3-modified histone octamer, were measured using a gator prime instrument (Gatorbio) at 30 °C with shaking speed of 1000 r.p.m. The biosensors of Streptavidin Probes (Gatorbio) were equilibrated in the binding buffer containing 20 mM Tris-HCl (pH 7.5), 100 mM NaCl, 1 mM DTT and 0.01% Tween-20 for 10 min and loaded with 10 nM biotin-nucleosome to a shift of up to 1.0 nM. The assay settings included (i) step of baseline in binding buffer for 120 s, (ii) step of association in various concentrations (2-fold serial dilution: for interactions with H3Ub2/H4K20me3-modified nucleosome, from 20 nM to 1.25 nM; for interactions with native nucleosome, from 500 nM to 31.25 nM) of DNMT1_351–1600_ protein solutions for 200 s, and (iii) step of dissociation in binding buffer for 300 s (for interactions with H3Ub2/H4K20me3-modified nucleosome) or 100 s (for interactions with unmodified nucleosome). The resulting data were processed with a 1:1 mode and analyzed using the Gatorbio data analysis software. The results were plotted in GraphPad Prism (v.6.0). All experiments were repeated twice.

### Preparation of DNMT1-nucleosome complexes for negative-stain and cryo-EM study

For preparation of WT or Mut^MHKKKQ^ DNMT1-^um^NCP complex, WT or Mut^MHKKKQ^ DNMT1_351–1600_ was mixed with the umNucDNA nucleosome in a molar ratio of 3:1 in a binding buffer containing 20 mM HEPES (pH 7.5), 50 mM NaCl, 5 mM DTT and 5% glycerol. The complex was incubated on ice for 10 min before subjected to crosslinking using the GraFix method^66^. Briefly, 300 pmol of sample was loaded on top of the buffer containing 50 mM HEPES (pH 7.5), 50 mM NaCl, 1 mM DTT, and concurrent 10–25% gradient of glycerol and 0–0.025% (v/v) gradient of glutaraldehyde in an ultracentrifuge tube. The mixture was centrifuged at 197,000 × *g* for 18 h in a Beckman SW41 rotor at 4 °C. Following centrifugation, the resulting gradients were partitioned in fractions of 500 µL from top to bottom. The fractions were examined by 5% native PAGE gel and the fractions containing DNMT1_351–1600_-^um^NCP complex were collected, buffer exchanged using a PD-10 column (Cytiva) equilibrated with the binding buffer. The purified complex was concentrated to 130 ng/μL and subjected to negative-stain electron microscopy and cryo-EM analysis.

To prepare the cryo-EM grids, 2.5 μL of the samples described above were applied onto freshly glow-discharged holey carbon grids (Quantifoil, copper, R1.2/1.3, 300 mesh for WT DNMT1-^um^NCP complex; Quantifoil, copper, R2/1, 300 mesh, with a 2 nm additional carbon layer, for Mut^MHKKKQ^ DNMT1-^um^NCP complex). The grids were incubated for 5 s at 4 °C with 100% humidity, blotted for 5 s with blot force of 1, then plunge-frozen in liquid ethane in a Vitrobot Mark IV instrument (Thermo Fisher Scientific).

### Cryo-EM data collection

The micrographs of the cryo-EM grids were initially screened on 200-kV T20 iCorr TEM equipped with a Gatan Ultrascan US4000 4k Camera at Electron Imaging Center for NanoMachines (EICN) at University of California, Los Angeles and a 120-kV Talos L120C TEM equipped with a CETA 164k x 4k CMOS camera at the Central Facility for Advanced Microscopy and Microanalysis (CFAMM) at University of California, Riverside. The cryo-EM data for the WT DNMT1-^um^NCP complex were collected at the Stanford-SLAC CryoEM Center (S^2^C^2^) on a Titan Krios electron microscope operated at 300 kV, equipped with a Gatan K3 imaging system using EPU (Thermo Fisher Scientific). The cryo-EM data for the Mut^MHKKKQ^ DNMT1-^um^NCP complex were collected on a Titan Krios electron microscope operating at 300 kV, equipped with a Gatan K3 imaging system, using Leginon (v 3.66)^67^ in the National Center for CryoEM Access and Training (NCCAT).

#### WT DNMT1-^um^NCP complex

A total of 16,915 movies were recorded at a nominal magnification of 81,000× in super-resolution mode with a pixel size of 1.1 Å per pixel (calibrated pixel size of 1.07 Å per pixel) and a defocus range of –1.5– to –2.1 µm. The data were collected with a total dose of 50 electrons Å^–2^ for a total exposure time of 2.0 s. Intermediate frames were recorded every 0.05 s for a total of 40 frames per micrograph.

#### Mut^MHKKKQ^ DNMT1-^um^NCP complex

A total of 31,434 movies were recorded at a nominal magnification of 81,000× in super-resolution mode with a calibrated pixel size of 1.07 Å per pixel (super-resolution of 0.535 Å per pixel) and a defocus range of –0.8 to –2.5 µm. The data were collected with a dose rate of 26.20 electrons Å^–2^s^–1^ for a total exposure time of 2.5 s. Intermediate frames were recorded every 0.05 s for a total of 50 frames per micrograph.

### Cryo-EM Data processing

#### General workflow

The movies were motion-corrected and dose-weighted using the patch-based motion correction module in CryoSPARC^68^. The contrast transfer function (CTF) of the resulting images was processed using the patch CTF estimation module. Subsequently, particles were picked using the TOPAZ method^69^. The particles were then extracted and subjected to Fourier binning and reference-free 2D classification to remove artifacts. Initial 3D models were generated using the ab-initio reconstruction module in CryoSPARC, followed by one or several rounds of heterogeneous refinement to select the classes with clearly defined density for the DNMT1-nucleosome complexes. After further classification and refinement, the resolutions of the final maps were determined by the Fourier shell correlation at 0.143 cut-off^70^.

#### WT DNMT1-^um^NCP complex

The particles were first extracted with a down-scaled pixel size of 3.30 Å per pixel. After the initial rounds of heterogeneous refinement in the general workflow, the particles with notable features for the DNMT1-NCP complex were re-extracted with a pixel size of 1.88 Å per pixel and subjected to another two rounds of heterogeneous refinement. The particles with the relatively strong density of DNMT1 (417,039 particles) were selected for non-uniform refinement, resulting in a 3.12-Å map. Next, the particles were subjected to further classification through two independent workflows. In the first workflow, the particles were subjected to DNMT1-masked particle subtraction in Relion (v4), and heterogeneous refinement and local refinement in CryoSPARC, leading to local map of DNMT1 of 4.86 Å. In parallel, particle subtraction and local refinement were performed for the CXXC-NCP component, resulting in a local map of 3.02 Å. The combined map derived from the local maps of DNMT1 and CXXC-NCP described above are denoted as State I DNMT1-^um^NCP complex. In the second workflow, the particles were subjected to sequential heterogeneous refinement, DNMT1-masked 3D classification and non-uniform refinement, resulting in a 3.84-Å map (State II). To clarify the density for the RFTS domain, the particles associated with State II density were further subjected to two rounds of RFTS-masked 3D classification in CryoSPARC. The particles with the best RFTS density were then selected for non-uniform refinement, resulting in a map of 4.41 Å (15,764 particles; State III).

#### Mut^MHKKKQ^ DNMT1-^um^NCP complex

The particles were first extracted with a down-scaled pixel size of 2.98 Å per pixel. After the initial rounds of heterogeneous refinement in the general workflow, the particles with best density for the DNMT1 molecule were re-extracted with a pixel size of 1.49 Å per pixel and subjected to non-uniform refinement, resulting in a 3.76-Å consensus map (287,638 particles). The particles were subjected to CXXC-NCP-masked particle subtraction and local refinement, resulting in a map of 3.60 Å. To further improve the density of the DNMT1 molecule, the particles for the consensus map were subjected to DNMT1-masked particle subtraction and focused classification in Relion (v4), with the class giving the best DNMT1 density converted back to CryoSPARC for sequential non-uniform refinement, resulting in a map of 4.12 Å (81,191 particles), and DNMT1-masked local refinement, resulting in a map of 5.24 Å. The consensus map and the local maps for the DNMT1 and CXXC-NCP were used to generate the composite map through the combine_focused_map module in the Phenix software package^71^. To clarify the density for the RFTS domain, the particles for the consensus map were also subjected to RFTS-masked 3D classification in CryoSPARC. The particles with the best RFTS density were then selected for non-uniform refinement, resulting in a map of 4.13 Å (72,038 particles).

### Model building

For both WT and mutant DNMT1-^um^NCP complexes, the structures of a nucleosome wrapped with 601 DNA (PDB 7JZV) and previously reported DNMT1 under various DNA-binding states were used to fit into the density map in ChimeraX^72^. In essence, the structural model of the DNMT1 BAH1-BAH2-MTase segment was built by fitting the crystal structure of DNMT1_351-1600_ (PDB 4WXX) into the density map. The CXXC domain was modeled by fitting the CXXC domain in the structure of DNMT1-CpG DNA complex (PDB 3PTA) into the density map. The final structural models were obtained after iterative model building in Coot^73^ and real-space refinement in Phenix.

### Cell lines and culture

The mouse embryonic stem cells (mESCs) with the knockout (KO) of *Dnmt1* (1KO-mESCs; a gift from Dr. M. Okano) were cultivated as previously^23^. In brief, mESCs were cultured on a gelatin-coated dish in the DMEM base medium (Gibco, cat# 11995065), supplemented with 15% of heat inactivated fetal bovine serum (FBS; VWR cat# 97068-085, heated at 56 °C for 30 min before use), 1% of antibiotics (penicillin and streptomycin), 1000 units/mL of leukemia inhibitory factor (LIF; EMD Millipore, cat# LIF2010), 55 μM of β-Mercaptoethanol (Gibco, cat# 21985023), and 1× non-essential amino acids (Gibco, cat# 11140050). 1KO-mESCs were transfected with the pPyCAGIP plasmid carrying 3×Flag-tagged DNMT1, either WT or mutant, using a Lipofectamine 3000 (Invitrogen)-based protocol. Forty-eight hours post-transfection, the cells were subject to selection with 1 μg/mL puromycin for 2 weeks. Independently derived clonal lines and pooled cell cultures were established, followed by characterization and verification such as immunoblotting.

The 1KO group represents the single isogenic parental cell line from which all other lines were derived. We analyzed this line in three technical replicates to establish a robust baseline for global 5mC levels in the absence of DNMT1. To ensure that the observed phenotypes were not due to random clonal effects, we established three independent clonal lines (biological replicates) for both WT and Mut^KQ^. Each of these three independent clones was then analyzed in three technical replicates (3 × 3 = 9), providing statistical confidence in the consistency of the rescue effect.

### Antibodies and Western blotting (WB)

Antibodies used for WB were anti-FLAG M2-Peroxidase (HRP) (Sigma-Aldrich, cat# A8592), anti-DNMT1 (Abcam, cat# ab13537) and anti-GAPDH (Cell Signaling Technology, cat# 2118 L). The freshly cultured cells were rinsed in ice-cold phosphate-buffered saline (PBS), suspended in the RIPA Lysis Buffer with the freshly added protease inhibitor (1 mM PMSF and 1× protease inhibitor cocktail, Roche), and then placed immediately on ice for 30 min. After centrifugation, the collected soluble cell lysate was added with 5× SDS-PAGE loading buffer and boiled at 95 °C for 10 min, followed by loading onto an SDS-PAGE gel for WB analysis.

### Co-immunoprecipitation (Co-IP) analysis

Cells (i.e., 1KO-mESCs reconstituted with 3×Flag-tagged DNMT1) were harvested, lysed in lysis buffer (50 mM Tris, pH 8.0, 120 mM NaCl, 0.5% NP-40, 0.1 mM EDTA, 10% glycerol, and protease and phosphatase inhibitors) and clarified by centrifugation. For immunoprecipitation, clarified lysates were incubated overnight at 4 °C with anti-FLAG M2 magnetic beads (Sigma, cat# M8823). The beads were washed, and bound proteins were eluted by boiling in SDS sample buffer. Total cell lysates and immunoprecipitated proteins were subsequently analyzed by western blotting using the indicated antibodies, including anti-UHRF1 (Proteintech, cat# 21402-1-AP, 1:1,000).

### Immunofluorescence and EdU Labeling

The 1KO-mESCs reconstituted with 3×Flag-tagged DNMT1, either WT or Mut^KQ^, were cultured on 0.1% gelatin-precoated coverslips. For EdU incorporation, cells were processed using the Click-iT EdU Cell Proliferation Kit for Imaging (Invitrogen, cat# C10339) according to the manufacturer’s instructions, with samples incubated in the Click-iT reaction cocktail for 30 min at room temperature. Cells were fixed in 4% PFA, permeabilized with 0.5% Triton X-100 in PBS, blocked in 3% BSA in PBS for 1 h, and incubated overnight at 4 °C with anti-FLAG primary antibody (Sigma-Aldrich, cat# F1804, 1:200). Secondary detection was performed using goat anti-mouse Alexa Fluor 488 (Invitrogen, cat# A11001; 2 h, room temperature). Specimens were mounted with Fluoromount-G containing DAPI (Invitrogen, cat# 00-4959-92) and imaged on a Zeiss LSM 880 Airyscan confocal microscope. Signal quantification was performed in Fiji/ImageJ.

### Quantification of 5-methyl-2′-deoxycytidine (5-mdC) in genomic DNA

Measurement of the global 5-mdC level in cells was carried out as previously^65^. Briefly, total genomic DNA was isolated and then digested into mononucleotides by nuclease P1 and alkaline phosphatase. After the enzymes in the digestion mixtures were removed by chloroform extraction, the resulting aqueous layer was dried, reconstituted in water, and subjected to LC–MS/MS analyses on a TSQ Altis triple quadrupole mass spectrometer for quantifications of 5-mdC and dG in the multiple-reaction monitoring mode. The amounts of 5-mdC and dG (in moles) in the nucleoside mixtures were calculated based on comparison of their signal intensities with their corresponding stable isotope-labeled standards and calibration curves. The final levels of 5-mdC were calculated as molar ratios of 5-mdC over dG.

### Cleavage Under Targets and Tagmentation (CUT&Tag) and data analysis

CUT&Tag was performed as described before^74^ with a commercially available kit according to the manufacturer’s instructions (EpiCypher CUTANA™ Direct-to-PCR CUT&Tag Protocol). Across all samples, 0.5 million of 1KO-mESCs with reconstituted expression of 3×Flag-tagged DNMT1 (either WT or mutant) were added with 5% of spike-in cells (namely, human 293 cells with stable expression of a 3×Flag-tagged chromatin factor), with the latter serving as the spike-in control for normalization. Cells were processed to isolate the nuclei in the NE buffer (20 mM HEPES-KOH, pH 7.9, 10 mM KCl, 0.1% Triton X-100, 20% Glycerol, 0.5 mM spermidine, 1× Roche cOmplete™ Protease Inhibitor). Nuclei were immobilized onto the activated ConA beads (Bangs Laboratories, cat# BP531) and then, such nuclei:bead complex sample was incubated with the antibodies of the protein target (3×Flag-tagged DNMT1; 1:100 dilution of the FLAG M2 antibody, Sigma-Aldrich, cat# F1804; anti-H3K27me3 antibody, Cell Signaling Technology, cat# 9733S) as primary antibody and Rabbit Anti-Mouse IgG H&L (Abcam, cat# ab46540; 1:100 dilution) or Anti-Rabbit Secondary Antibody (EpiCypher, cat# 13-1047) as secondary antibody. Next, the sample was permeabilized in the digitonin-containing buffer (with 0.02% digitonin), followed by washing in the digitonin buffer, incubation with pAG-Tn5, and another washing in the digitonin buffer to remove the unbound pAG-Tn5. After the final wash, the Tagmentation Buffer (the digitonin 300 buffer plus 10 mM MgCl_2_) was added to activate the pAG-Tn5 transposase, which cleaves the DNA near the target protein while simultaneously inserting the sequencing adapters, followed by incubation on a nutator for 1 h at 37 °C. Reactions were quenched with the SDS Release Buffer (0.1% SDS, 10 mM TAPS, pH 8.5), and the DNA was purified for library preparation by PCR. Sequencing was conducted in an Illumina NextSeq 1000/2000 Sequencing System.

Fastq files (trim_galore v.0.6.10) were mapped to the main reference genome (mm10) by using bowtie2^75^ (v.2.4.4), and for the spike-in control normalization, fastq files were mapped to the spike-in reference genome (hg38) using STAR^76^ (v.2.7.11a). The non-primary alignment and PCR duplicates were removed from the aligned data using Samtools^77^ (v.1.10) (-q 30 -F 1804 -f 2 for unique mapping reads), the Picard “MarkDuplicates” function (v.2.18.2), and bedtools^78^ (v.2.30.0) (the “intersect” function used for excluding the genome blacklist region), respectively. A scale factor was calculated by comparing the total number of spike-in alignment reads among different samples and then used for normalization. For peak annotation, two different methods were performed. Peak calling was performed using MACS2^79^ (v.2.2.6) (macs2 callpeak -f BAMPE -g mm --keep-dup 1 --cutoff-analysis -q 0.05), The annotating program of HOMER^80^ v.4.8.2 (annotatePeaks.pl) was performed to associate the called peaks with genomic features, while the R package ChIPpeakAnno^81^ (v.3.34.1) was used to assign the peaks to the nearby genes (bindingType = “fullRange”, bindingRegion = c(−1,1), ignore.peak.strand = TRUE, select = “bestOne”). Motif analysis was performed using hypergeometric optimization of motif enrichment (HOMER) (v.4.8.2). Deeptools^82^ (v.3.2.0) was used to generate the bigwig files, heatmaps and averaged plotting of CUT&Tag signals. Genomic binding profiles were generated using the deepTools “bamCoverage” and “computeMatrix” functions. The called peaks, as well as the CGIs with coordinates acquired from the UCSC Genome Browser, were visualized in the Integrative Genomics Viewer (IGV)^83^.

### Whole genome-wide bisulfite sequencing (WGBS) and data analysis

WGBS was conducted as previously described^84^, with 1 μg of total genomic DNA and the commercial kits (Admera Health). Briefly, the isolated genomic DNA was subjected to bisulfite treatment by using the EZ DNA Methylation-Gold kit (Zymo Research, cat# D5005) per the vendor-provided instructions. Then, the bisulfite-converted single-stranded DNA (ssDNA) was recovered and used for library construction using the Accel-NGS@Methyl-seq DNA Library kit (Swift BioSciences, cat# 30024) based on the manufacturer’s instructions. DNA enrichment was performed using PCR with the primers compatible with Illumina deep sequencing. After assessing the concentration and molecular size, the prepared WGBS libraries were sequenced on an Illumina NovaSeq S4 sequencer platform (150-bp read lengths in a paired-end mode), with an output of approximately 750 million reads or 375 million in each direction per sample.

The raw data were trimmed by trim_galore (--paired -q 30 --stringency 5 --phred33 -e 0.1 --length 20 --clip_R1 10 --clip_R2 20 --three_prime_clip_R1 10 --three_prime_clip_R2 15), with FastQC (v.0.11.7) and MultiQC (v.1.22.2) used for quality control. To evaluate the rate of bisulfite conversion, we employed BCREval (v.1.3)^85^ and the bisulfite conversion ratio was calculated to be over 99.5% across all samples. Read mapping and methylated cytosine calling were generated using the Bismark (v.0.24.2)^86^ pipeline. The data were further filtered, normalized and merged using the R package methylKit^87^ (v.1.26.0) (filterByCoverage(lo.count = 10, lo.perc = NULL, hi.count = NULL, hi.perc = 99.9), normalizeCoverage(method = “median”)). The above steps and results of quality control and WGBS data processing are summarized in Supplementary Data 2.

The volcano plots of differentially methylated cytosine sites (DMCs in the CpG and non-CpG content) were shown using ggplot2, followed by the cut-off standard set to the *P* value less than 1 × 10^–3^ (or 1 × 10^–4^) and the difference in overall methylation level over 25%. R package genomation^88^ was performed to annotate the genomic sites as the overlap with CpG island (CGI), CGI shore, promoter, exon, intron or intergenic region. The heatmap of the enrichment of hyper-DMCs or hyper-DMRs was generated using the R package. Metagene plot of methylation level around CGIs was shown using R packages. The box plots of CpG methylation differences between the samples with DNMT1 WT versus the mutant at different genomic features (CGIs, shores, promoters, exons and intergenic regions) were generated using R packages.

For calling for DMRs, we employed the R package, DSS (v.2.56.0)^89^, with a cut-off standard set to the adjusted *P* value (*Padj*) less than 0.01 and the difference in overall methylation level over 15% for those CpG sites with at least 10 coverages across all samples. *P*-value is calculated using logistic regression and then adjusted to *Padj* value using sliding linear model (SLIM) method. Annotation of DMCs and DMRs was performed by HOMER v.4.8.2 (annotatePeaks.pl) and shown in Supplementary Data 3 and 5, respectively. Each DMR was assigned a nearby gene, followed by the Gene Ontology (GO) and KEGG pathway enrichment analysis by using the R package clusterProfiler (v.4.16.0)^90^. Functional enrichment analysis on DMRs was independently performed by using the Genomic Regions Enrichment of Annotations Tool^91^ (GREAT v.4.0.4). IGV was used to present the location of DMRs or CGIs, as well as the absolute methylation level (based on WGBS profiles), at specific genomic loci.

Correlations between CpG density (mm10 mouse genome) and DNA methylation or DNMT1 binding were assessed using custom Perl and R scripts. The genome (chromosomes 1–19, X, and Y) was divided into non-overlapping 1-kb windows, excluding those with ≥10% undetermined nucleotides (N). CpG counts in each window were used to calculate CpG density, and windows were ranked from low to high, yielding approximately 2.65 million windows. To reduce noise and enhance statistical power, windows were smoothed by averaging ~530 consecutive windows into single bins, resulting in 5,000 bins. The top 10% of CpG density bins (500 bins, 1.5–17.7%) were further subdivided into ten equal subgroups to provide higher resolution. Average methylation levels and DNMT1 binding signals were calculated for each bin and subgroup, with DNMT1 binding quantified from CUT&Tag experiments in WT and Mut^KQ^ samples as the log_2_ converted value of Reads Per Kilobase per Million mapped reads (RPKM; added with an arbitrary value of 1 for conversion purposes).

### RNA sequencing and data analysis

RNA-seq was performed as previously described^92,93^. Total RNA was extracted from cells utilizing the RNeasy Plus Mini kit (Qiagen, 74134). Subsequently, multiplexed RNA sequencing libraries were constructed using the NEBNext Ultra II RNA Library Prep Kit (NEB, E7770). RNA-seq libraries were subjected to high-throughput sequencing on an Illumina platform (NextSeq 500, HiSeq 4000, or NovaSeq 6000) according to the manufacturer’s instructions. The FASTQ files were trimmed using Trim Galore and aligned to the mm10 mouse genome using STAR v2.4.2 with the following parameters: --outSAMunmapped Within --outSAMtype BAM Unsorted --quantMode TranscriptomeSAM. The transcript abundance for each sample was estimated with salmon (v1.4.0) to quantify the transcriptome defined by GENCODE vM25. Gene-level counts (summed across isoforms) were pre-filtered to remove low-expression genes. Only genes with a total count of no less than 10 across all samples were retained for downstream analysis. Differentially expressed genes (DEGs) were defined using DESeq2 (v1.38.2) in R, with genes considered significantly differentially expressed at an adjusted *p*-value (*p*adj) < 0.05 and an absolute value of log_2_ fold-change > 0.58.

#### Principal component analysis

Gene expression values were rlog-transformed using DESeq2. PCA was performed on the transformed expression matrix using prcomp in R, and the percentage of variance explained by each principal component was calculated from the eigenvalues. Samples were plotted in PC1–PC2 space using ggplot2 and ggrepel, colored by genotype and time point.

#### Lineage marker heatmap analysis

Gene-level Fragments Per Kilobase of transcript per Million mapped fragments (FPKM) values were log_2_(FPKM + 1)-transformed and z-score scaled across samples. A curated panel of lineage markers^94–97^, spanning naive pluripotency, general pluripotency, primitive endoderm, early commitment, ectoderm, mesoderm, and definitive endoderm, was visualized as a heatmap using pheatmap. Samples were hierarchically clustered by Euclidean distance, with dendrograms reordered using dendsort to follow the day 0–3–5 time course.

#### GO enrichment analysis

DEGs (*p*adj < 0.05, |log2 fold-change| > 0.58) were split into up- and down-regulated sets for each comparison. Gene symbols were converted to Entrez IDs using bitr (org.Mm.eg.db) and GO Biological Process terms were tested for enrichment using enrichGO (clusterProfiler; BH-adjusted *p* < 0.01, *q* < 0.05).

#### Gene set enrichment analysis (GSEA)

Genes were ranked by sign(log2 fold-change) × −log_10_(*p*-value), with *p*-values capped at 1e-300 to avoid numerical underflow. GSEA was performed against the MSigDB Hallmark gene set collection (msigdbr) using the GSEA function (clusterProfiler; gene set size 10–500, *p* < 0.05). Development- and differentiation-related gene sets (*q* < 0.25) were visualized as bubble plots (NES vs. gene set, sized by gene set size, colored by FDR *q*-value) and as running-enrichment-score plots (gseaplot2).

### The mESC differentiation

#### Embryoid body (EB) formation

The mESCs were dissociated from the gelatin-coated culture plates and seeded onto ultra-low attachment plates (Corning, cat# CLS3471) at a density of 1 × 10^5^ cells/mL in the differentiation medium (which is the mESC culture medium without addition of LIF). The medium was replaced every 48 h and EBs were collected at different time points (such as days 3 and 5 post-differentiation).

#### Neuronal differentiation

For monolayer neuronal differentiation, mESCs were plated on gelatin-coated plates at a density of 1 × 10⁴ cells/cm² in ES cell culture medium. 24 h later, the medium was replaced with the differentiation medium supplemented with 1 μM RA (Sigma-Aldrich, cat# R2625) to induce differentiation. The medium was refreshed every 48 h, and cells were harvested at various time points post-differentiation.

### Quantitative reverse transcription PCR (qRT–PCR)

Total RNA was extracted using the RNeasy Plus Mini Kit (Qiagen, cat# 74136) and reverse transcribed into cDNA with the High-Capacity cDNA Reverse Transcription Kit (Applied Biosystems, cat# 4368813). Quantitative PCR was performed in triplicate using the iTaq Universal SYBR Green Supermix (Bio-Rad, cat# 1725124) on a ViiA 7 Real-Time PCR System (Applied Biosystems). Gene expression levels were normalized to an internal control (such as beta-Actin) using the ΔCt method. In some cases, a second normalization to WT control cells was applied to calculate relative fold changes. Primer sequences are listed in Supplementary Data 6.

### Reporting summary

Further information on research design is available in the Nature Portfolio Reporting Summary linked to this article.

## Supplementary information

Supplementary Information

Description of Additional Supplementary Files

Supplementary Data 1

Supplementary Data 2

Supplementary Data 3

Supplementary Data 4

Supplementary Data 5

Supplementary Data 6

Supplementary Data 7

Reporting Summary

Transparent Peer Review file

## Source data

Source Data

## Data Availability

The consensus, local and composite 3D cryo-EM maps have been deposited in the Electron Microscopy Data Bank under the corresponding accession numbers. WT DNMT1-^um^NCP complex: EMD-73698 (composite map); EMD-73713 (consensus map); EMD-73701EMD-73708 (local map for CXXC-NCP); Mut^MHKKKQ^ DNMT1-^um^NCP complex: EMD-48749 (composite map), (consensus map), EMD-77709 (local map for CXXC-NCP), EMD-77710 (local map for Mut^MHKKKQ^ DNMT1), and EMD-77711 (RFTS-focused map). Atomic coordinates for the structural models have been deposited in the Protein Data Bank under accession code: 9Z07: WT DNMT1-^um^NCP complex; and 9MYS: Mut^MHKKKQ^ DNMT1-^um^NCP complex. The atomic coordinates used for model building and structural comparisons were downloaded from the Protein Data Bank under the following accession codes: 7JZV, 7XI9, 3PTA, 4WXX. The RNA-seq, CUT&Tag and WGBS genomic datasets generated in this work are deposited to the NCBI GEO under accession numbers GSE338522, GSE287117 and GSE287140, respectively. All custom scripts are available from the authors upon request. Source data are provided with this paper.
