# Supplementary material for: The DNMT1 CXXC domain senses CpG islands for DNA methylation inhibition
Source: Nat Commun. 2026 Sep 18;17:10970. doi: 10.1038/s41467-026-77872-8 (PMC13683344; doi:10.1038/s41467-026-77872-8)
Supplement: Supplementary file 1 [file 41467_2026_77872_MOESM10_ESM.pdf]

nature portfolio

Corresponding author(s): Gang, G. Wang, Jikui Song

Last updated by author(s): Aug 25, 2026

Reporting Summary

Nature Portfolio wishes to improve the reproducibility of the work that we publish. This form provides structure for consistency and transparency in reporting. For further information on Nature Portfolio policies, see our Editorial Policies and the Editorial Policy Checklist.

Statistics

For all statistical analyses, confirm that the following items are present in the figure legend, table legend, main text, or Methods section.

n/a	Confirmed	
☐	☒	The exact sample size (n) for each experimental group/condition, given as a discrete number and unit of measurement
☐	☒	A statement on whether measurements were taken from distinct samples or whether the same sample was measured repeatedly
☐	☒	The statistical test(s) used AND whether they are one- or two-sided<br>*Only common tests should be described solely by name; describe more complex techniques in the Methods section.*
☒	☐	A description of all covariates tested
☐	☒	A description of any assumptions or corrections, such as tests of normality and adjustment for multiple comparisons
☐	☒	A full description of the statistical parameters including central tendency (e.g. means) or other basic estimates (e.g. regression coefficient) AND variation (e.g. standard deviation) or associated estimates of uncertainty (e.g. confidence intervals)
☐	☒	For null hypothesis testing, the test statistic (e.g. F, t, r) with confidence intervals, effect sizes, degrees of freedom and P value noted<br>*Give P values as exact values whenever suitable.*
☒	☐	For Bayesian analysis, information on the choice of priors and Markov chain Monte Carlo settings
☒	☐	For hierarchical and complex designs, identification of the appropriate level for tests and full reporting of outcomes
☐	☒	Estimates of effect sizes (e.g. Cohen's d, Pearson's r), indicating how they were calculated

Our web collection on statistics for biologists contains articles on many of the points above.

Software and code

Policy information about availability of computer code

Data collection	Cryo-EM data were collected using the Leginon software at Nation Center for CryoEM Access and Training (NCCAT), Stanford-SLAC CryoEM Center (S2C2). CUT&Tag sequencing data was conducted in an Illumina NextSeq 1000/2000 Sequencing System. WGBS data were collected on the Illumina NovaSeq S4 sequencer platform.
Data analysis	For structural study, cryoSPARC v4.0.1 was used for image processing, particle classification, density refinement, local resolution estimation and map sharpening. Relion v4.1 was used for particle subtraction and alignment-free 3D classification. UCSF ChimeraX v1.5 and Coot v0.9.6 were used for modeling building. PHENIX v1.20.1 was used for Real-Space structure calculation, and Pymol v2.5.2 was used for structural analysis. GraphPad Prism v6.01 was used for graph plotting. GatorOne v2.10.4 was used for BLI data analysis. trim_galore (v.0.6.10), bowtie2 (v.2.4.4), STAR (v.2.7.11a), Samtools (v.1.10), Picard (v.2.18.2), bedtools (v.2.30.0), MACS2 (v.2.2.6), HOMER (v.4.8.2), ChIPpeakAnno (v.3.34.1), Deeptools (v.3.2.0), Integrative Genomics Viewer (v.2.19.1), FastQC (v.0.11.7), MultiQC (v.1.22.2), BCREval (v.1.3), Bismark (v.0.24.2), methylKit (v.1.26.0), DSS (v.2.56.0), clusterProfiler (v.4.16.0), GREAT (v.4.0.4).

For manuscripts utilizing custom algorithms or software that are central to the research but not yet described in published literature, software must be made available to editors and reviewers. We strongly encourage code deposition in a community repository (e.g. GitHub). See the Nature Portfolio guidelines for submitting code & software for further information.

Data

Policy information about availability of data

All manuscripts must include a data availability statement. This statement should provide the following information, where applicable:

- Accession codes, unique identifiers, or web links for publicly available datasets
- A description of any restrictions on data availability
- For clinical datasets or third party data, please ensure that the statement adheres to our policy

The consensus, local and composite 3D cryo-EM maps have been deposited in the Electron Microscopy Data Bank under the following accession numbers: WT DNMT1-umNCP complex, EMD-73698 (composite map), EMD-73713 (consensus map), EMD-73708 (local map for CXXC-NCP),EMD-73701 (local map for WT DNMT1), ; MutMHKKKQ DNMT1-umNCP complex, EMD-48749 (composite map), EMD-48748 (consensus map), EMD-77709 (local map for CXXC-NCP), EMD-77710 (local map for MutMHKKKQ DNMT1), EMD-77711 (RFTS-focused map), respectively. Atomic coordinates for the structural models have been deposited in the Protein Data Bank under accession code: WT DNMT1-umNCP complex, 9Z07; and MutMHKKKQ DNMT1-umNCP complex, 9MYS, respectively. Next-generation sequencing data are deposited to the NCBI GEO under accession number GSE287117 (CUT&Tag data; token: inupmokgvrgjjex), GSE338522 (RNA-seq data; token: qlursuagndgvjwv) and GSE287140 (WGBS data; token: cxoxwoaypzqhrax).

Research involving human participants, their data, or biological material

Policy information about studies with human participants or human data. See also policy information about sex, gender (identity/presentation), and sexual orientation and race, ethnicity and racism.

Reporting on sex and gender	n/a
Reporting on race, ethnicity, or other socially relevant groupings	n/a
Population characteristics	n/a
Recruitment	n/a
Ethics oversight	n/a

Note that full information on the approval of the study protocol must also be provided in the manuscript.

Field-specific reporting

Please select the one below that is the best fit for your research. If you are not sure, read the appropriate sections before making your selection.

☒ Life sciences ☐ Behavioural & social sciences ☐ Ecological, evolutionary & environmental sciences

For a reference copy of the document with all sections, see nature.com/documents/nr-reporting-summary-flat.pdf

Life sciences study design

All studies must disclose on these points even when the disclosure is negative.

Sample size	Sample size was chosen to ensure reproducibility of the results at affordable costs. Biochemical assays were completed using wild type or mutants of DNMT1 fragments. The sample size is sufficient to delineate the mutational effects of DNMT1. At least 3 independent experiments were carried for Western blot. Whole genome-wide bisulfite sequencing and CUT&Tag experiments were performed with two biological replicates. LC-MS experiments were performed at least three biological replicates.
Data exclusions	No data exclusion.
Replication	All attempts at replication were successful and noted in the relevant figure legends. The BLI binding was performed twice with consistent results. The DNA methylation assays were performed in triplicates with consistent results.
Randomization	The assays performed in this study require a rational approach for activity comparison. Therefore, randomization is not applicable to our experimental set up.
Blinding	The assays performed in this study require rational design. Therefore, blinding is not applicable to any biochemical or cellular assay performed in this study.

Reporting for specific materials, systems and methods

We require information from authors about some types of materials, experimental systems and methods used in many studies. Here, indicate whether each material, system or method listed is relevant to your study. If you are not sure if a list item applies to your research, read the appropriate section before selecting a response.

Materials & experimental systems

n/a	Involved in the study	
☐	☒	Antibodies
☐	☒	Eukaryotic cell lines
☒	☐	Palaeontology and archaeology
☒	☐	Animals and other organisms
☒	☐	Clinical data
☒	☐	Dual use research of concern
☒	☐	Plants

Methods

n/a	Involved in the study	
☒	☐	ChIP-seq
☒	☐	Flow cytometry
☒	☐	MRI-based neuroimaging

Antibodies

Antibodies used	Antibodies used for western blotting were anti-FLAG M2-Peroxidase (HRP) (Sigma-Aldrich, cat# A8592), anti-DNMT1 (abcam, cat# ab13537) and anti-GAPDH (Cell Signaling Technology, cat# 2118L). Antibodies used for CUT&Tag were anti-FLAG M2 antibody (Sigma-Aldrich, F1804) as primary antibody and Rabbit Anti-Mouse IgG H&L (Abcam, cat# ab46540) as secondary antibody.
Validation	All antibodies used are commercially available and validated by corresponding companies.<br>FLAG M2-Peroxidase (HRP) antibody: https://www.sigmaaldrich.com/US/en/product/sigma/a8592<br>DNMT1 antibody: https://www.abcam.com/en-us/products/primary-antibodies/dnmt1-antibody-60b12201-ab13537<br>GAPDH antibody: https://www.cellsignal.com/products/primary-antibodies/gapdh-14c10-rabbit-mab/2118<br>FLAG M2 antibody: https://www.sigmaaldrich.com/US/en/product/sigma/f1804<br>Rabbit Anti-Mouse IgG H&L antibody: https://www.abcam.com/en-us/products/secondary-antibodies/rabbit-mouse-igg-h-l-ab46540

Eukaryotic cell lines

Policy information about cell lines and Sex and Gender in Research

Cell line source(s)	Mouse embryonic stem cells with knockout of Dnmt1 were cultivated.
Authentication	Authentication of cell line identity, including that of parental and derived lines, was ensured by the Cell Culture Facility affiliated to the Duke University School of Medicine using the genetic signature profiling and fingerprinting analysis.
Mycoplasma contamination	Every 1-2 month, a routine examination of cell lines in culture for any possible mycoplasma contamination was carried out using MycoAlert Mycoplasma Detection Kit (Lonza). No mycoplasma contamination was identified.
Commonly misidentified lines (See ICLAC register)	No commonly misidentified cell lines were used in the study.

Plants

Seed stocks	n/a
Novel plant genotypes	n/a
Authentication	n/a
