# Supplementary material for: The DNMT1 CXXC domain senses CpG islands for DNA methylation inhibition
Source: Nat Commun. 2026 Sep 18;17:10970. doi: 10.1038/s41467-026-77872-8 (PMC13683344; doi:10.1038/s41467-026-77872-8)
Supplement: Supplementary file 2 [file 41467_2026_77872_MOESM11_ESM.pdf]

nature portfolio

Peer Review File

The DNMT1 CXXC domain senses CpG islands for DNA methylation inhibition

Corresponding Author: Dr Jikui Song

This file contains all reviewer reports in order by version, followed by all author rebuttals in order by version.

Version 0:

Reviewer comments:

Reviewer #1

(Remarks to the Author)
In this manuscript by Fang et al., the authors reveal the structural and functional basis for how DNMT1 avoids the aberrant methylation of CpG islands (CGIs). They demonstrate that the DNMT1 CXXC domain binds to unmethylated CpG sites on the nucleosome, triggering an autoinhibitory conformation that blocks the enzymatic active site. This mechanism operates independently of the previously characterized RFTS-mediated regulation. The authors further valid these findings in vivo, showing that disrupting the CXXC-CpG interaction leads to CGI hypermethylation and defects in stem cell differentiation. Altogether, the study is well-conceived and executed. I appreciate the high quality of the Cryo-EM data and the rigorous validation using biochemical and genomic approaches. The findings provide a critical missing link in understanding how DNA methylation patterns are faithfully maintained. I consider this manuscript of sufficient novelty and importance. It would be great if the authors can address specific concerns below to make the conclusion more solid.

1. There are several spelling errors that need correction: Fig5a Y-axis should be “DNA Methylation”; Page 12, line 19, should be “CGI-rich” instead of “GCI-rich”; Page 16, line 7, should be “carboxylcytosine”.

2. The in vitro experiments using H3Ub2-modified nucleosomes mimic a UHRF1-independent model. However, the in vivo environment is more complex. The observed CGI hypermethylation in MutKQ cells could potentially result from enhanced recruitment by UHRF1 (due to conformational changes in MutKQ) rather than solely the loss of autoinhibition. Suggested experiments:
(1). Perform Co-IP to demonstrate whether the interaction between DNMT1 and UHRF1 differs between WT and MutKQ cells.
(2). Perform UHRF1 knockdown in MutKQ cells. If CGI methylation remains high, it supports the authors' intrinsic activity hypothesis; if it decreases, it suggests UHRF1 dependence.

3. Since MutKQ loses binding at CGIs, does the protein redistribute to other genomic regions, such as transposons? The authors should analyze the methylation status and binding levels at these repetitive elements more comprehensively.

4. The authors should examine H3K9me3 levels at CGIs in the MutKQ group. Unchanged H3K9me3 levels would further confirm that the observed gene silencing is directly driven by DNA methylation rather than heterochromatin spreading.

5. To rigorously prove the specific "safeguarding" role of the CXXC domain, it is highly suggested to include an RFTS-defective mutant. If the model holds, this mutant (with an intact CXXC domain) should maintain normal CGI methylation despite potential global hyperactivity.

6. In Figure 4a, the 1KO group has a sample size of n=3. Please clarify if this is statistically robust enough compared to the other groups.

7. In Figures 5h and 5i, the 1KO group is missing. Including 1KO data is essential to determine whether the MutKQ phenotype represents a complete or partial loss of function. Please add this data or explain the exclusion.

8. Figure 5h relies on RT-qPCR for a few selected genes (e.g., Gata6, Nkx2-5). To conclusively show that CGI

hypermethylation leads to global gene silencing, the authors should perform RNA-seq comparing WT and MutKQ cells.

9. The model proposed in Figure 6 strictly distinguishes between S phase and Non-S phase mechanisms. However, the CUT&Tag data presented are from asynchronous cells, which cannot support phase-specific conclusions. It is unclear if the CXXC-mediated protection is distinct from or equally important during the S phase. Suggested Experiments: Perform cell synchronization (e.g., using nocodazole) to collect G1 and S phase cells separately. Then repeat CUT&Tag or qPCR in these synchronized populations to verify if the binding differences between WT and MutKQ align with the proposed model.

Reviewer #2

(Remarks to the Author)
Cytosine methylation in CpG sequence plays a critical role in gametogenesis, embryogenesis, and development. DNA methylation patterns established during development are faithfully maintained through the coordinated actions of the maintenance DNA methyltransferase DNMT1 and its recruiter and activator, UHRF1. Disruption of DNA methylation maintenance leads to aberrant methylation patterns, which in turn cause genome instability and contribute to tumorigenesis. Therefore, elucidating the molecular mechanisms underlying DNA methylation maintenance remains a central and important topic in the life sciences.
Several structural studies of DNMT1 have revealed its autoinhibitory mechanism mediated by the RFTS domain, the exclusion of unmethylated CpG DNA by the CXXC domain, and its activation by ubiquitinated histone H3 and PAF15. However, the molecular mechanisms by which DNMT1 associates with chromatin and executes its function in a chromatin context remain incompletely understood. In this study, Fang et al. determined the structure of DNMT1 bound to the nucleosome containing ubiquitinated histone H3 and H4K20me3. The authors also performed biological experiments to demonstrate the functional significance of unmethylated CpG recognition by the DNMT1 CXXC domain. While the structural analysis is of high quality, details regarding sample preparation are limited. In addition, several aspects of the biological experiments require further clarification and improvement.

1) Given that the CUT&TAG methodology is inherently biased towards open chromatin regions such as promoters and enhancers, which often exhibit high CpG density, appropriate controls are required to support the statement that “DNMT1 preferentially binds to sites exhibiting high CG density.” Please show the read depth of the and also include a negative control such as the 1KO-mESCs without transfection of exogenous DNMT1 proteins, in relevant figures (e.g., Figs. 3F and 4H).
2) The gain of DNA methylation in CGIs per se, and in the neighboring CGI shores, appears to be very modest (Fig 4H). Therefore, the statement that the authors observed “most pronounced increases of 5mC at the CGI shore regions”, seems overstated and should be revised to a more accurate description , such as “there was a modest increase of 5mC at the CGI shore regions” to give a fair description of the results.
3) Related to the point above, what is the median methylation level within CGIs (not including the shores) in cells expressing WT or mutant DNMT1? I Is there a statistically significant difference between these conditions?
4) The weak gain of DNA methylation in CGI shores may simply reflect a genome-wide, modest increase in DNA methylation. To address this possibility, the authors should quantify DNA methylation level in well-characterized heterochromatic regions, such as H3K9me3-enriched domains and specific ERV or LINE families.

5) As the authors propose a functional link between DNA methylation gain around CGIs and altered developmental gene expression during ESC differentiation, it is important to further define DNA methylation changes according to the transcriptional and/or epigenetic states in CGIs. Do the authors see a difference in the propensity for methylation gain in different types of CGIs, such as active vs repressed, and/or bound or not bound by the polycomb group (PcG) of proteins?

6) Changes in the expression level of developmental genes shown in Fig 5h should be accompanied by more detailed information. Gata6, Nkx2-5 and Eomes are typical targets of polycomb group (PcG) of proteins, and therefore it is expected that the expression of such genes will mainly depend on binding of PcG factors at the CGI regions of those genes. The authors should check H3K27me3 levels and/or binding of PRC1/2 (polycomb repressive complexes 1 or 2, such as RING1B, EZH2 and SUZ12) factors at those CGIs, to describe whether the observed perturbation in the transcription levels of these genes is related to changes in local chromatin regulation by PcG proteins.

7) On page 6, lines 4–6 under “Structural details of the DNMT1 CXXC domain–CpG interaction at the dyad”, the sentence starting with “rather than …” is difficult to understand and would benefit from clarification or rephrasing.

8) In Figure 2d–e and Supplementary Figures 2j, 2l, and 4e, the regions highlighted by the color coding are not clearly explained. It would be helpful to explicitly indicate which regions are being shown, for example by following the labeling style used in Figure 2a.

9) Please show the purity of each histone protein and the histone octamer by SDS-PAGE, and also the assembled nucleosomes by native PAGE.

Version 1:

Reviewer comments:

Reviewer #1

(Remarks to the Author)
The authors have adequately addressed all of my concerns, and the additional experiments and revisions have substantially strengthened the manuscript. I have no further comments and support publication of the revised manuscript.

Reviewer #2

(Remarks to the Author)
The authors have adequately addressed the reviewer's comments and suggestions.
I hope that my comments have helped improve the quality of the manuscript.
I recommend acceptance of this manuscript.

Open Access This Peer Review File is licensed under a Creative Commons Attribution 4.0 International License, which permits use, sharing, adaptation, distribution and reproduction in any medium or format, as long as you give appropriate credit to the original author(s) and the source, provide a link to the Creative Commons license, and indicate if changes were made.
In cases where reviewers are anonymous, credit should be given to 'Anonymous Referee' and the source.
The images or other third party material in this Peer Review File are included in the article's Creative Commons license, unless indicated otherwise in a credit line to the material. If material is not included in the article's Creative Commons license and your intended use is not permitted by statutory regulation or exceeds the permitted use, you will need to obtain permission directly from the copyright holder.
To view a copy of this license, visit https://creativecommons.org/licenses/by/4.0/

General Response

We thank both reviewers for their collective efforts in reviewing our manuscript, their positive view of our work, and their constructive comments for improving the manuscript. As outlined below in the point-by-point response (marked in blue), we have now systematically addressed all the critiques raised and have updated the revised manuscript accordingly (marked in red).

Responses to Reviewer 1

Reviewer #1 (Remarks to the Author):

In this manuscript by Fang et al., the authors reveal the structural and functional basis for how DNMT1 avoids the aberrant methylation of CpG islands (CGIs). They demonstrate that the DNMT1 CXXC domain binds to unmethylated CpG sites on the nucleosome, triggering an autoinhibitory conformation that blocks the enzymatic active site. This mechanism operates independently of the previously characterized RFTS-mediated regulation. The authors further valid these findings in vivo, showing that disrupting the CXXC-CpG interaction leads to CGI hypermethylation and defects in stem cell differentiation.
Altogether, the study is well-conceived and executed. I appreciate the high quality of the Cryo-EM data and the rigorous validation using biochemical and genomic approaches. The findings provide a critical missing link in understanding how DNA methylation patterns are faithfully maintained. I consider this manuscript of sufficient novelty and importance. It would be great if the authors can address specific concerns below to make the conclusion more solid.

We thank the Reviewer for the careful review and positive assessment of this work. We agree with the Reviewer that this study, through establishing a safeguarding role of the DNMT1 CXXC domain in CGI hypomethylation, "provide a critical missing link in understanding how DNA methylation patterns are faithfully maintained". We also thank the Reviewer for the constructive suggestions in improving the manuscript and have addressed his/her critiques as below.

1. There are several spelling errors that need correction: Fig5a Y-axis should be "DNA Methylation"; Page 12, line 19, should be "CGI-rich" instead of "GCI-rich"; Page 16, line 7, should be "carboxylcytosine".

We thank the Reviewer for pointing out these typographical errors and have corrected them in the revised manuscript.

2. The in vitro experiments using H3Ub2-modified nucleosomes mimic a UHRF1-independent model. However, the in vivo environment is more complex. The observed CGI hypermethylation in MutKQ cells could potentially result from enhanced recruitment by UHRF1 (due to conformational changes in MutKQ) rather than solely the loss of autoinhibition. Suggested experiments:

We thank the Reviewer for raising the point. As the Reviewer may well know, the most well-established mechanism by which UHRF1 regulates DNMT1-mediated maintenance DNA methylation lies in UHRF1-catalyzed dual monoubiquitylation of H3 (H3Ub2), which is recognized by the RFTS domain of DNMT1, thereby recruiting and activating DNMT1 [1-3]. In this study, we used the H3Ub2-modified nucleosomes to represent the product of UHRF1, providing a model system for a UHRF1-regulated event. We have clarified this point in the revised manuscript (Line 83-84). On the other hand, direct interaction between DNMT1 and UHRF1 has also been observed and mapped to various domains of DNMT1[4], the functional significance of which remains to be clarified.

(1). Perform Co-IP to demonstrate whether the interaction between DNMT1 and UHRF1 differs between WT and MutKQ cells.

Following the Reviewer's suggestion, we performed co-immunoprecipitation (Co-IP) experiments for DNMT1-FLAG and UHRF1 in both WT and Mut^{KQ} cells (Fig. R1). Our results demonstrated no obvious difference in UHRF1 level between WT and Mut^{KQ} DNMT1 pull-downs. This observation supported the notion that the hypermethylation phenotype in Mut^{KQ} cells is not a consequence of enhanced recruitment via UHRF1, but rather the relief of the CXXC domain-mediated DNMT1 autoinhibition. We also wish to clarify that the CGI binding of Mut^{KQ} DNMT1 was decreased, rather than increased than WT DNMT1 (Fig. 3e,f), which further argues against the possibility that the CGI hypermethylation arises from increased DNMT1 recruitment.

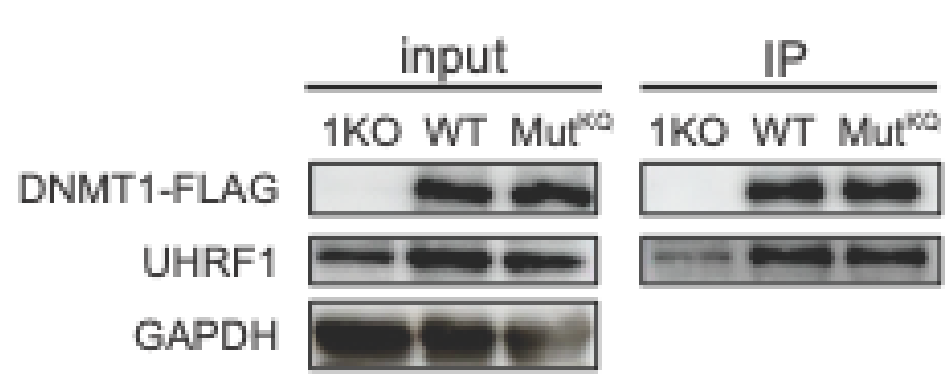

Figure R1. Co-IP analysis of DNMT1 and UHRF1 in wild-type (WT), DNMT1-knockout (1KO), and Mut^{KQ} cells. Total cell lysates (input, left panels) and proteins immunoprecipitated with anti-FLAG M2 magnetic beads (Sigma, M8823) (IP, right panels) were analyzed by western blotting using the indicated antibodies, including anti-UHRF1 (Proteintech, 21402-1-AP, 1:1,000). GAPDH served as a loading control for the input fractions.

We have included this data as Supplementary Fig. 7d and clarified the point in the revised manuscript.

(2). Perform UHRF1 knockdown in MutKQ cells. If CGI methylation remains high, it supports the authors' intrinsic activity hypothesis; if it decreases, it suggests UHRF1 dependence.

We appreciate the Reviewer for the suggestion. As clarified above, this study supports both the critical role of UHRF1 in target recruitment of DNMT1 and the downstream role of DNMT1 autoinhibition in CGI hypomethylation. Therefore, UHRF1 knockdown is predicted to cause genome-wide DNA hypomethylation, including CGIs, in both WT and Mut^{KQ} cells. The suggested experiment may further be complicated by the recent finding of the non-canonical function of UHRF1, including the interaction with DNMT3A/3B and the suppression of TET2-mediated active demethylation at CGIs; as a result, UHRF1-depleted cells exhibit more profound hypomethylation than DNMT1-depleted cells at CGIs [5]. For the reasons above, we respectfully request this experiment not to be pursued in this study.

3. Since MutKQ loses binding at CGIs, does the protein redistribute to other genomic regions, such as transposons? The authors should analyze the methylation status and binding levels at these repetitive elements more comprehensively.

Following the Reviewer’s suggestion, we performed a comprehensive analysis of DNA methylation and protein occupancy at major classes of repetitive elements (REs).

Genome-wide redistribution of DNMT1 protein: Our CUT&Tag analysis confirms that WT DNMT1 is preferentially targeted to genomic regions with high CpG density (Fig. 3b-d), especifically CGIs and CGI-rich regulatory elements (shores) (Fig. 3e,f). In contrast, the Mut^{KQ} mutation significantly reduces DNMT1 binding at these regions (Fig. 3c-f and Fig. R2A top panel), supporting the notion that the CXXC domain senses unmodified CpG sites to enrich DNMT1 at CGIs. Furthermore, we observed that Mut^{KQ} exhibits slightly increased binding at genomic regions with lower CpG density, including H3K9me3-enriched regions and repetitive elements such as SINE, LINE, and LTR elements (Fig. 3c and Fig. R2A bottom panel), when compared to the highly targeted occupancy of WT DNMT1 at CGIs. This observation suggests that disrupting the CXXC-CpG interaction leads to modest redistribution of DNMT1 across the genome.

Methylation Profiling of Transposons: Detailed analysis of our Whole-Genome Bisulfite Sequencing (WGBS) data reveals a global shift in hypermethylation across multiple genomic features (Figure R2B). In addition to DNA hypermethylation at CGIs and CGI-shores, subtle increase in DNA methylation was observed for repetitive elements, which was overshadowed by the near-saturation baseline methylation of the corresponding regions.

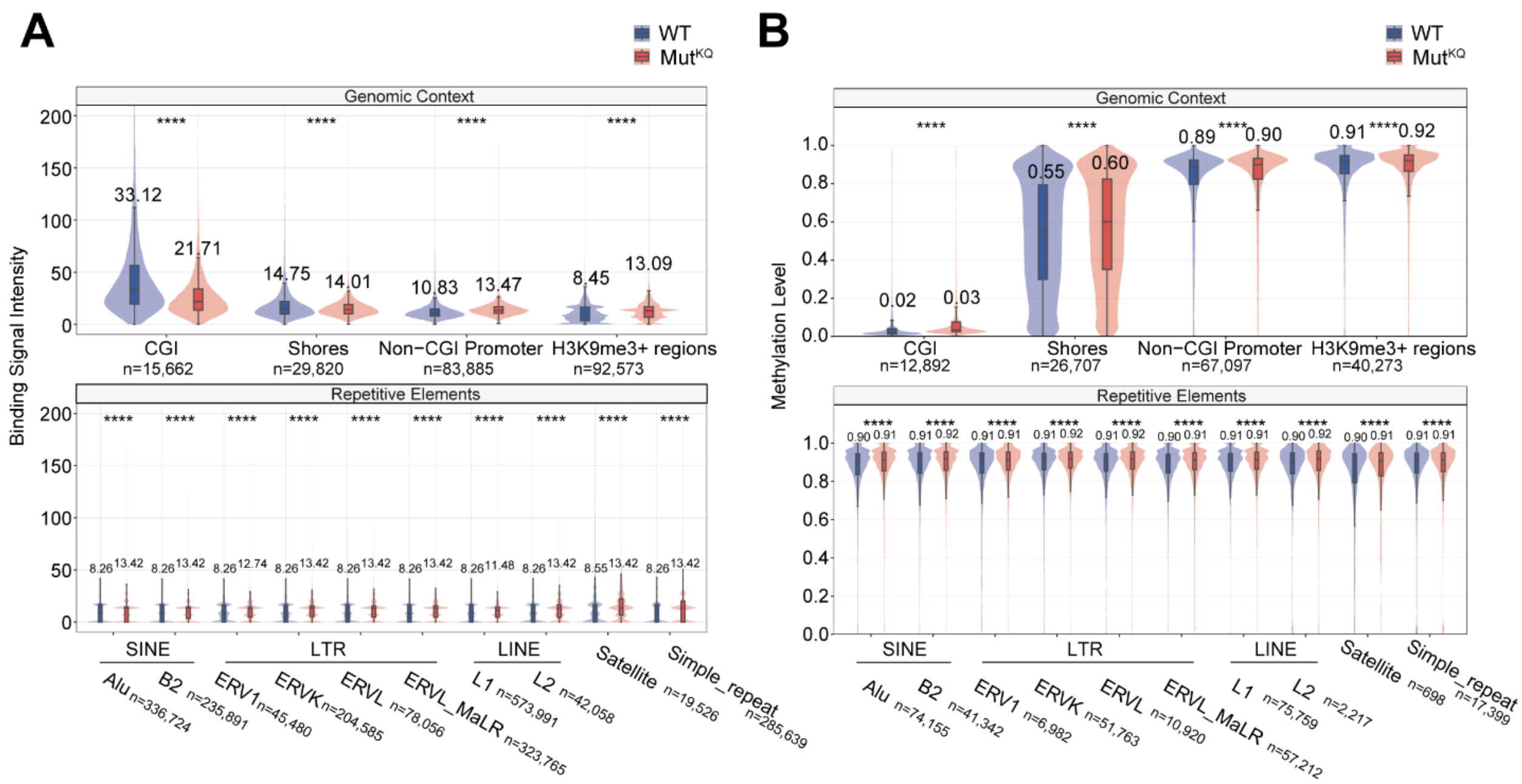

Figure R2. Genomic analysis of DNMT1 binding and DNA methylation in WT and Mut^{KQ} cells. (A) DNMT1 binding signal intensities and (B) global DNA methylation levels evaluated across distinct genomic features (top panels) and repetitive elements (bottom panels) in WT (blue) vs. Mut^{KQ} (red) cells. Median values are indicated numerically above each distribution cluster. The total number of evaluated loci (n) is provided beneath each feature name. Statistical significance was determined using a paired Wilcoxon signed-rank test (****$p < 0.0001$). CGI, CpG island; Non-CGI, areas outside CpG islands; SINE, short interspersed nuclear element; ERV, endogenous retrovirus; LTR, long terminal repeats; LINE (L1/L2), long interspersed nuclear element.

We have updated Supplementary Fig. 7f&9a to include this comprehensive RE analysis.

4. The authors should examine H3K9me3 levels at CGIs in the MutKQ group. Unchanged H3K9me3 levels would further confirm that the observed gene silencing is directly driven by DNA methylation rather than heterochromatin spreading.

The notion that enhanced CGI methylation is attributed to the loss of DNMT1 autoinhibition, rather than heterochromatin silencing, is supported by the observation that (i) Mut^{KQ} led to reduced, rather than increased, binding to CGIs (Fig. 3e,f), and (ii) the Mut^{KQ} does not affect the DNMT1-UHRF1 interaction (Fig. R1 per the Reviewer's request). Following the Reviewer's suggestion, we performed H3K9me3 CUT&Tag experiment in WT and Mut^{KQ} cells, which shows that H3K9me3 signal was very low across CGIs, with a modest decrease, rather than increase, observed for Mut^{KQ} compared to WT cells. The molecular basis for the slight decrease of H3K9me3 in Mut^{KQ} cells awaits further investigation. Nevertheless, this data falls in line with notion that enhanced CGI methylation is not attributed to H3K9me3 spreading.

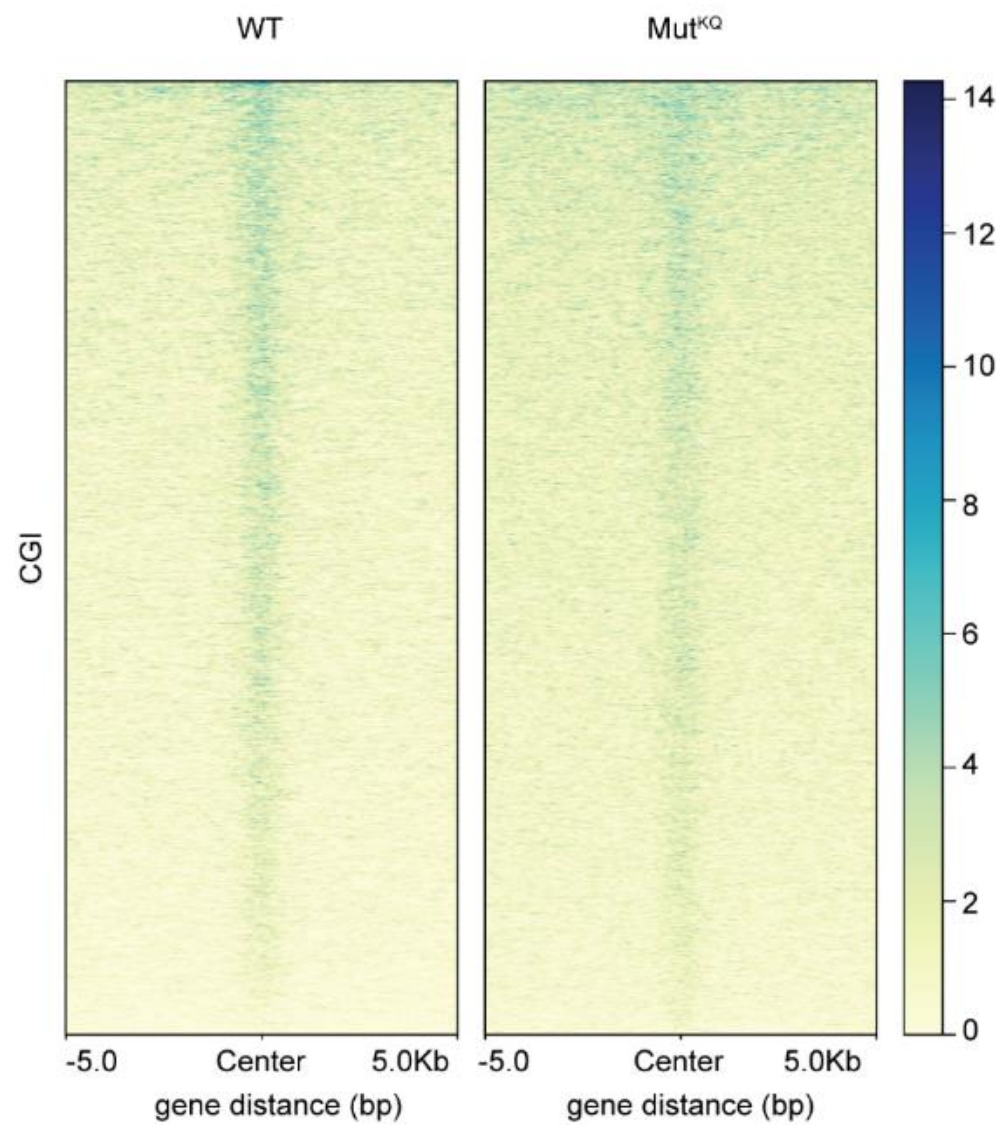

Figure R3. H3K9me3 signal at CpG islands in WT and Mut^{KQ} cells. Heatmap of H3K9me3 CUT&Tag signal across all CpG islands (CGIs) genome-wide in WT and Mut^{KQ} cells, generated with deepTools computeMatrix/plotHeatmap, centered on CGIs ± 5 kb. Rows represent individual CGIs; color scale indicates normalized H3K9me3 signal, shared between WT and Mut^{KQ}.

5. To rigorously prove the specific "safeguarding" role of the CXXC domain, it is highly suggested to include an RFTS-defective mutant. If the model holds, this mutant (with an intact CXXC domain) should maintain normal CGI methylation despite potential global hyperactivity.

We appreciate the reviewer's suggestion. We wish to clarify that as reported in our previous study[6], the RFTS-defective mutant (e.g. W465A or W464A/W465A), despite hyperactive on methylating naked DNA *in vitro*, led to genomic hypomethylation in cells, which was attributed to disrupted interaction with H3Ub2.

Following the Reviewer's comment, we re-examined our previously published eRRBS data for the RFTS-defective mutants W465A and W464A/W465A[6] (Fig. R4). As expected, whereas these RFTS-defective mutants possess a functional CXXC domain, they exhibit a profound global loss of DNA methylation in cells, supporting the critical role of UHRF1-catalyzed H3Ub2 in DNA methylation maintenance.

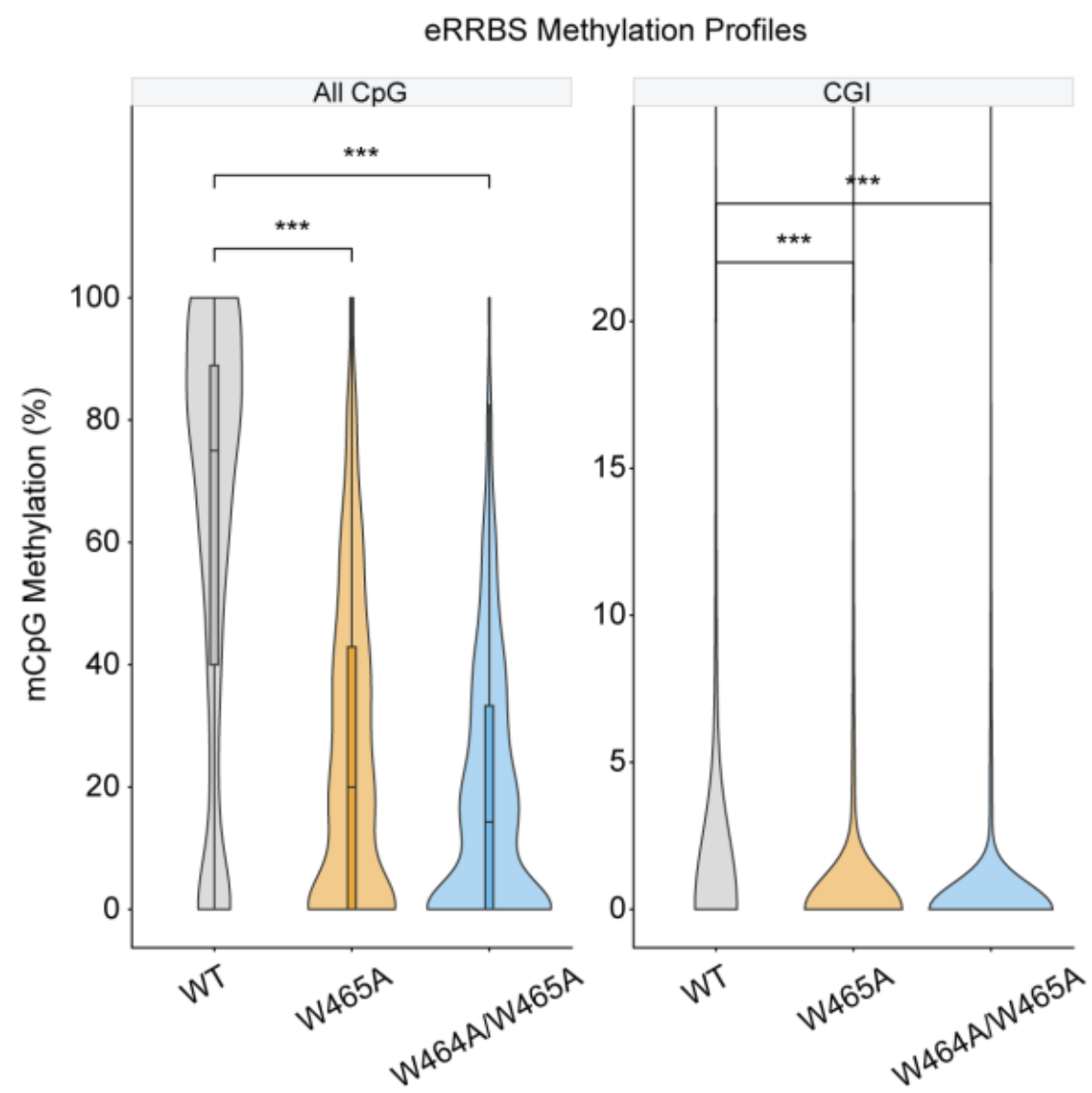

Figure R4. Enhanced Reduced Representation Bisulfite Sequencing (eRRBS) methylation profiles in wild-type (WT, grey), W465A (orange), and double-mutant W464A/W465A (blue) cells. Violin plots illustrate the distribution of fractional methylation percentages across all sequenced CpG sites (left) and specifically within CpG islands (CGI, right). Overlaid boxplots outline the median values and interquartile distribution spans. Pairwise significance brackets indicate cross-group variations relative to WT, evaluated using a two-tailed Student's *t*-test (***$p < 0.001$).

6. In Figure 4a, the 1KO group has a sample size of n=3. Please clarify if this is statistically robust enough compared to the other groups.

We thank the reviewer for the suggestion. In this study, the differences in sample sizes (n=3 for 1KO vs. n=9 for WT and Mut^{KQ}) reflect the distinction between the parental baseline control and the independently derived experimental rescue lines. Parental 1KO-ESCs (n=3): The 1KO group represents the single isogenic parental cell line from which all other lines were derived. We analyzed this line in three technical replicates to establish a robust baseline for global 5mC levels in the absence of DNMT1. WT and Mut^{KQ} Rescue Groups (n=9 each): To ensure that the observed phenotypes were not due to random clonal effects, we established three independent clonal lines (biological replicates) for both WT and Mut^{KQ}. Each of these three independent clones was then analyzed in three technical replicates (3 × 3 = 9), providing statistical confidence in the consistency of the rescue effect.

We have clarified this point in the revised manuscript in the method section (Line 772-778).

7. In Figures 5h and 5i, the 1KO group is missing. Including 1KO data is essential to determine whether the MutKQ phenotype represents a complete or partial loss of function. Please add this data or explain the exclusion.

The Reviewer's point is well taken. Following the Reviewer's suggestion, we reperformed RT-qPCR and have included the 1KO data in the revised manuscript (Fig. R5), which indicates that for the assessed marker genes—Gata6, Eomes, Hoxa9, Neurog1, and Neurog2—the Mut^{KQ} phenotype closely mirrors the 1KO state: Whereas WT cells show robust induction of these markers upon differentiation, both Mut^{KQ} and 1KO cells failed to activate these developmental programs. This data has been included as Fig. 6f,g in the revised manuscript.

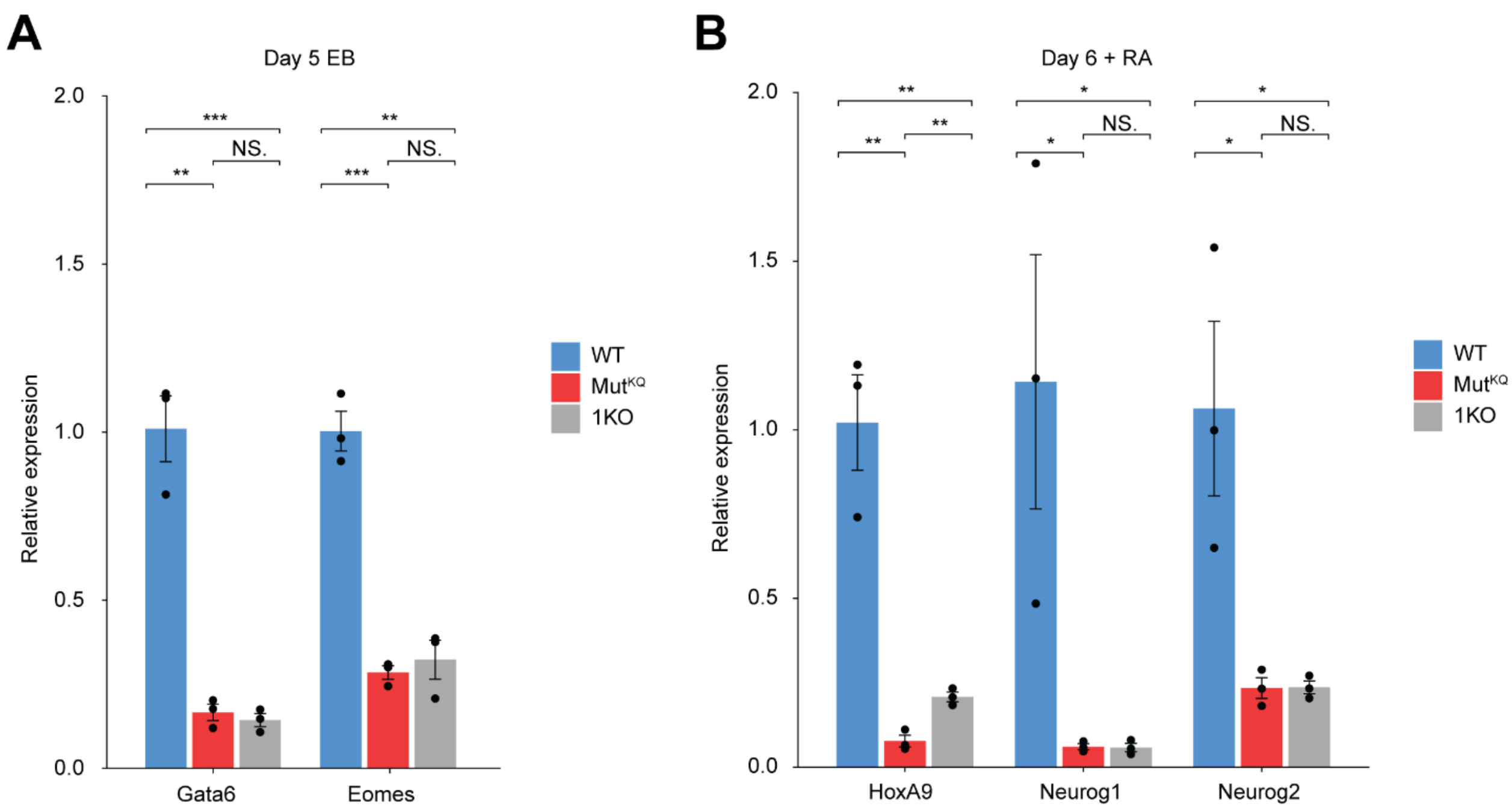

Figure R5. Quantitative RT-PCR (RT-qPCR) analysis of select lineage-specific marker genes in wild-type (WT, blue), Mut^{KQ} (red), and parental DNMT1-knockout (1KO; grey). (A) Relative expression of endodermal (Gata6) and mesodermal (Eomes) markers at Day 5 of embryoid body (EB) formation. (B) Relative expression of homeobox (HoxA9) and neurogenic (Neurog1, Neurog2) targets at Day 6 of differentiation following Retinoic Acid (+RA) induction. Data points represent individual biological replicates (n = 3), with error bars representing the standard error of the mean (SEM). Statistical significance was determined using a two-tailed Student's t-test (**p* < 0.05, ***p* < 0.01, ****p* < 0.001; NS, non-significant).

8. Figure 5h relies on RT-qPCR for a few selected genes (e.g., Gata6, Nkx2-5). To conclusively show that CGI hypermethylation leads to global gene silencing, the authors should perform RNA-seq comparing WT and MutKQ cells.

We thank the Reviewer for the excellent suggestion. In revision, we performed a time-course RNA-seq analysis comparing WT and Mut^{KQ} cells at key stages of embryoid body (EB) formation (Day 0, Day 3, and Day 5) (Fig. R6).

Principal Component Analysis (PCA) confirmed high reproducibility and tight clustering among all biological replicates, providing a robust foundation for our differential expression analysis (Fig. R6A). Our analysis revealed that while WT cells follow a standard, highly coordinated and expected differentiation cascade (Fig. R6B), this program was significantly impaired in both Mut^{KQ} and 1KO lines (Fig. R6C). We observed a marked failure to activate key lineage-specific marker genes. Of note, several pluripotency and early specification genes expected to increase during the early transition (e.g., Pou5f1, Utf1, Dnmt3a, Pou3f1, Sox3, Lef1, Otx2 and Sall4) failed to reach expected expression levels by Day 3, and a broad suite of differentiation markers for later stages (e.g., Gata4, Gata6, Dkk1, T, Mixl1, Eomes, Gsc, Lhx1, Evx1, Fgf8, Shh, Flt1, Kdr, Aplnr, Foxa1, Foxa2, Hhex and Cer1) showed significantly lower expression in Mut^{KQ} and 1KO cells compared to WT at Day 5 (Fig. R6C).

Consistently, Gene Ontology (GO) (Fig. R7A) and Gene Set Enrichment Analysis (GSEA) (Fig. R7B) using MSigDB demonstrated that pathways fundamentally required for embryogenesis, axis specification, and lineage commitment were systematically down-regulated or disrupted in Mut^{KQ} cells at Day 5 (e.g., mesoderm formation, mesoderm morphogenesis, mesoderm development, mesenchyme development, stem cell differentiation, embryonic organ morphogenesis, cell fate commitment, somite development, etc) (Fig. R7C).

Together, these genome-wide functional enrichment results complement our single-locus observations (i.e., qRT-PCR), providing clear evidence that aberrant CGI hypermethylation in Mut^{KQ} cells blocks the activation of vital developmental regulons. This broad transcriptional silencing ultimately locks the cells in a differentiation-arrested state, preventing proper exiting from pluripotency, resulting in a subsequent failure in the differentiation process.

We have included these data as Fig. 6a,b and Supplementary Fig. 11 and 12a,b in the revised manuscript.

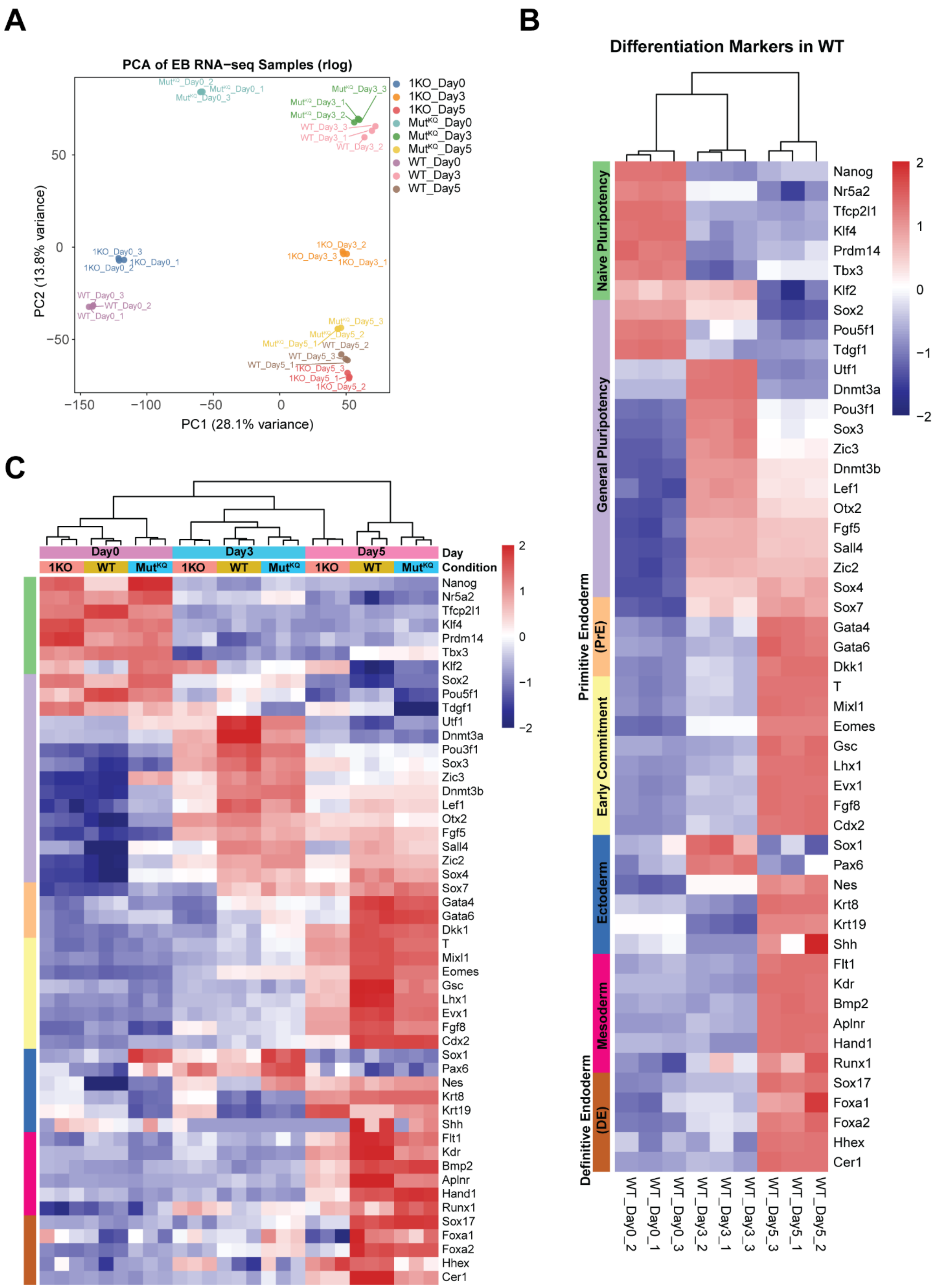

A
PCA of EB RNA−seq Samples (rlog)
PC1 (28.1% variance)
PC2 (13.8% variance)
1KO_Day0
1KO_Day3
1KO_Day5
Mut^{KQ}_Day0
Mut^{KQ}_Day3
Mut^{KQ}_Day5
WT_Day0
WT_Day3
WT_Day5
B
Differentiation Markers in WT
Naive Pluripotency
General Pluripotency
Primitive Endoderm (PrE)
Early Commitment
Ectoderm
Mesoderm
Definitive Endoderm (DE)
WT_Day0_2
WT_Day0_1
WT_Day0_3
WT_Day3_2
WT_Day3_1
WT_Day3_3
WT_Day5_3
WT_Day5_1
WT_Day5_2
C
Day
Condition
Day0
Day3
Day5
1KO
WT
Mut^{KQ}
Nanog
Nr5a2
Tfcp2l1
Klf4
Prdm14
Tbx3
Klf2
Sox2
Pou5f1
Tdgf1
Utf1
Dnmt3a
Pou3f1
Sox3
Zic3
Dnmt3b
Lef1
Otx2
Fgf5
Sall4
Zic2
Sox4
Sox7
Gata4
Gata6
Dkk1
T
Mixl1
Eomes
Gsc
Lhx1
Evx1
Fgf8
Cdx2
Sox1
Pax6
Nes
Krt8
Krt19
Shh
Flt1
Kdr
Bmp2
Aplnr
Hand1
Runx1
Sox17
Foxa1
Foxa2
Hhex
Cer1

Figure R6. RNA-seq analysis of WT and mut^{KQ} cells. (A) Principal Component Analysis (PCA) of RNA-seq samples (rlog-transformed counts) spanning a differentiation time course at Day 0, Day 3, and Day 5 for wild-type (WT), Mut^{KQ} and 1KO from mouse embryonic stem cells (mESCs) to embryoid body (EB). Replicates (n = 3) cluster tightly by time point and genotype. (B) Hierarchical clustering and heatmap presentation of z-score normalized expression values from log-transformed FPKM data (i.e., $\log_2$(FPKM + 1)) for standard lineage specification and pluripotency markers across the differentiation time course in WT cells alone, establishing expected baseline kinetics across embryonic layers. (C) Heatmap comparing marker expression across WT, Mut^{KQ} and 1KO genotypes across all time points. Left annotation color bars delineate functional developmental categories: Naive Pluripotency, General Pluripotency, Primitive Endoderm (PrE), Early Commitment, Ectoderm, Mesoderm, and Definitive Endoderm (DE). Selected markers were compiled and referenced based on established differentiation frameworks[7-10]. Color scales denote relative expression levels (z-score of normalized counts).

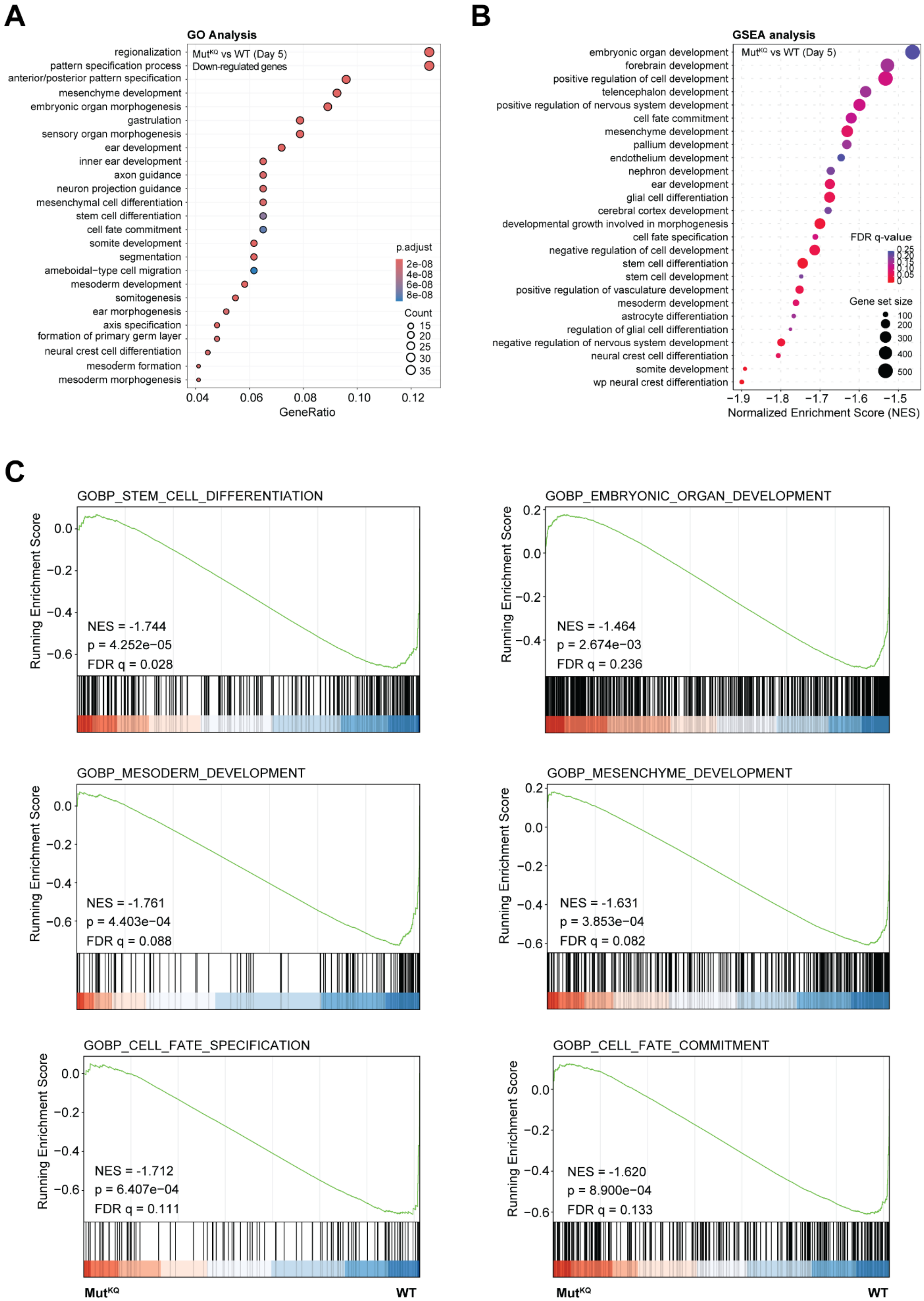
A
GO Analysis
Mut^KQ vs WT (Day 5)
Down-regulated genes
regionalization
pattern specification process
anterior/posterior pattern specification
mesenchyme development
embryonic organ morphogenesis
gastrulation
sensory organ morphogenesis
ear development
inner ear development
axon guidance
neuron projection guidance
mesenchymal cell differentiation
stem cell differentiation
cell fate commitment
somite development
segmentation
ameboidal−type cell migration
mesoderm development
somitogenesis
ear morphogenesis
axis specification
formation of primary germ layer
neural crest cell differentiation
mesoderm formation
mesoderm morphogenesis
p.adjust
2e-08
4e-08
6e-08
8e-08
Count
15
20
25
30
35
0.04
0.06
0.08
0.10
0.12
GeneRatio
B
GSEA analysis
Mut^KQ vs WT (Day 5)
embryonic organ development
forebrain development
positive regulation of cell development
telencephalon development
positive regulation of nervous system development
cell fate commitment
mesenchyme development
pallium development
endothelium development
nephron development
ear development
glial cell differentiation
cerebral cortex development
developmental growth involved in morphogenesis
cell fate specification
negative regulation of cell development
stem cell differentiation
stem cell development
positive regulation of vasculature development
mesoderm development
astrocyte differentiation
regulation of glial cell differentiation
negative regulation of nervous system development
neural crest cell differentiation
somite development
wp neural crest differentiation
FDR q-value
0.25
0.20
0.15
0.10
0.05
0
Gene set size
100
200
300
400
500
−1.9
−1.8
−1.7
−1.6
−1.5
Normalized Enrichment Score (NES)
C
GOBP_STEM_CELL_DIFFERENTIATION
Running Enrichment Score
NES = -1.744
p = 4.252e−05
FDR q = 0.028
GOBP_EMBRYONIC_ORGAN_DEVELOPMENT
NES = -1.464
p = 2.674e−03
FDR q = 0.236
GOBP_MESODERM_DEVELOPMENT
NES = -1.761
p = 4.403e−04
FDR q = 0.088
GOBP_MESENCHYME_DEVELOPMENT
NES = -1.631
p = 3.853e−04
FDR q = 0.082
GOBP_CELL_FATE_SPECIFICATION
NES = -1.712
p = 6.407e−04
FDR q = 0.111
GOBP_CELL_FATE_COMMITMENT
NES = -1.620
p = 8.900e−04
FDR q = 0.133
Mut^KQ
WT
Mut^KQ
WT

Figure R7. (A) Over-representation analysis (ORA) of significantly down-regulated genes (p.adjust < 0.05, $\log_2$FoldChange < -0.58) in Mut^{KQ} versus WT cells at Day 5 of embryoid body (EB) differentiation, implemented via clusterProfiler::enrichGO mapping against the org.Mm.eg.db mouse genome database (Ontology: Biological Process). The x-axis represents the Gene Ratio, dot size scales with absolute gene count, and point color corresponds to Benjamini-Hochberg (BH) adjusted *p*-values (p.adjust). (B) Gene Set Enrichment Analysis (GSEA) profile. Pre-ranked gene lists were generated by scoring total transcripts via signed significance ranking (sign($\log_2$FoldChange) × -$\log_{10}$(*p*-value)), capped at a maximum value of 300 to break ties. Normalized Enrichment Scores (NES) are plotted against Molecular Signatures Database (MSigDB) annotated pathways, filtered for development- and differentiation-associated terms under an FDR *q*-value cutoff of < 0.25. (C) Representative GSEA running enrichment plots for essential multi-lineage and morphogenetic gene sets derived from the MSigDB, including Stem Cell Differentiation, Embryonic Organ Development, Mesoderm Development, Mesenchyme Development, Cell Fate Specification, and Cell Fate Commitment. Corresponding NES, *p*-values, and FDR *q*-values are listed internally. The skewing of the running enrichment score asymmetry toward the right (WT-enriched spectrum) represents a systematic and coordinated transcriptional down-regulation of primary lineage-commitment networks within the Mut^{KQ} cells.

9. The model proposed in Figure 6 strictly distinguishes between S phase and Non-S phase mechanisms. However, the CUT&Tag data presented are from asynchronous cells, which cannot support phase-specific conclusions. It is unclear if the CXXC-mediated protection is distinct from or equally important during the S phase. Suggested Experiments: Perform cell synchronization (e.g., using nocodazole) to collect G1 and S phase cells separately. Then repeat CUT&Tag or qPCR in these synchronized populations to verify if the binding differences between WT and MutKQ align with the proposed model.

We agree with the Reviewer that CUT&Tag with asynchronous cells represents a population average. Given that DNMT1 is localized to replication fork across the entire genome during S phase, the observed chromatin binding of DNMT1 is likely influenced mainly by a mechanism other than UHRF1-mediated histone ubiquitylation (H3Ub2). For clarity, we have modified the model to remove the distinction between S phase and non-S phase. Instead, we propose that the CXXC-mediated inhibition occurs in both H3Ub2-positive and H3Ub2-negative conditions, based on the knowledge that DNMT1-mediated maintenance DNA methylation is critically regulated by UHRF1[11, 12].

Following the Reviewer's comment, we performed immunofluorescence experiments using a short EdU pulse to unambiguously mark S-phase cells (EdU+) versus non-S-phase cells (EdU−) within the same asynchronous population, followed by co-staining for DNMT1-FLAG (Fig. R8). In non-S-phase (EdU−) cells, both WT and Mut^{KQ} DNMT1-FLAG

show a diffuse nucleoplasmic distribution. In S-phase (EdU+) cells, both WT and Mut^{KQ} show DNMT1-FLAG reorganizing into discrete puncta that overlap extensively with EdU-labeled replication foci (merge, yellow puncta). This data supports that WT and Mut^{KQ} DNMT1 binds to chromatin similarly in S phase.

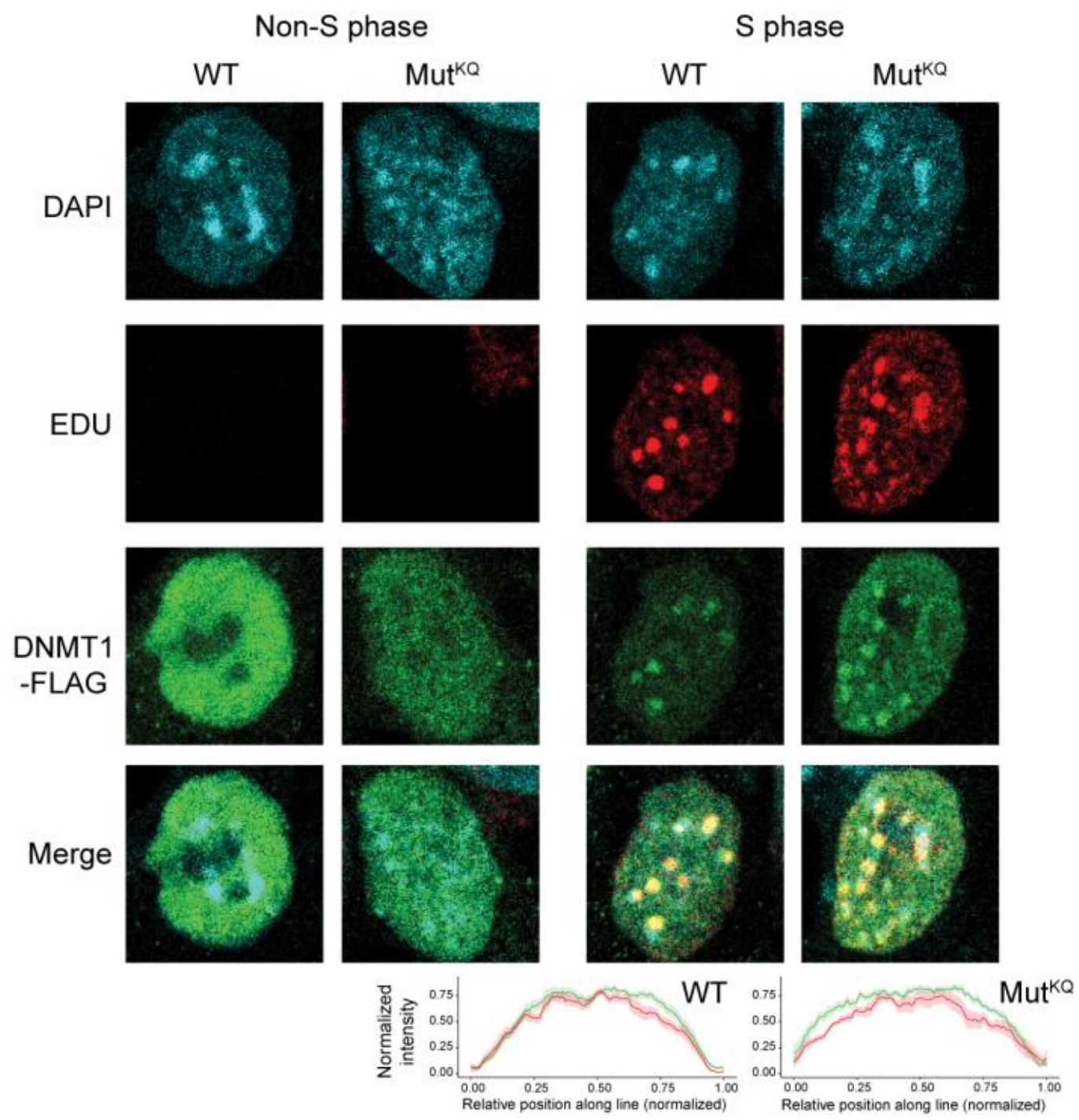

Figure R8. Cell cycle-dependent DNMT1 localization in WT and Mut^{KQ} cells. Representative immunofluorescence images of DAPI (cyan), EdU (red; 30 min pulse labeling nascent DNA to mark S-phase cells), and DNMT1-FLAG (green) in WT and Mut^{KQ} cells, comparing non-S-phase (EdU-negative) and S-phase (EdU-positive) cells. Merge panels show overlay of all three channels; regions of EdU/DNMT1-FLAG overlap appear yellow. (Bottom right) Intensity profiles of EdU (red) and DNMT1-FLAG (green) signal plotted as a function of distance (μm) across representative S-phase nuclei for WT and Mut^{KQ} cells. Fluorescence intensity was extracted along a square drawn across each nucleus using Fiji/ImageJ. Data are mean±SEM (n = 10 cells). The mean intensities are shown as solid lines. The intensity values within 1 SEM are shown in shade.

We have included the data as Supplementary Fig. 7e in the revised manuscript.

In addition, we would like to note that a recent study (Ref. 44 in the revised manuscript), published during the revision of our work, shows that DNMT1 is enriched in CGIs under steady-state conditions but redistributed to heterochromatin under inhibitor treatment, in MV-4-11 cells[13], further supporting the model proposed in this study.

Response to Reviewer 2

Reviewer #2 (Remarks to the Author):

Cytosine methylation in CpG sequence plays a critical role in gametogenesis, embryogenesis, and development. DNA methylation patterns established during development are faithfully maintained through the coordinated actions of the maintenance DNA methyltransferase DNMT1 and its recruiter and activator, UHRF1. Disruption of DNA methylation maintenance leads to aberrant methylation patterns, which in turn cause genome instability and contribute to tumorigenesis. Therefore, elucidating the molecular mechanisms underlying DNA methylation maintenance remains a central and important topic in the life sciences.

Several structural studies of DNMT1 have revealed its autoinhibitory mechanism mediated by the RFTS domain, the exclusion of unmethylated CpG DNA by the CXXC domain, and its activation by ubiquitinated histone H3 and PAF15. However, the molecular mechanisms by which DNMT1 associates with chromatin and executes its function in a chromatin context remain incompletely understood. In this study, Fang et al. determined the structure of DNMT1 bound to the nucleosome containing ubiquitinated histone H3 and H4K20me3. The authors also performed biological experiments to demonstrate the functional significance of unmethylated CpG recognition by the DNMT1 CXXC domain. While the structural analysis is of high quality, details regarding sample preparation are limited. In addition, several aspects of the biological experiments require further clarification and improvement.

We thank the Reviewer for the summary of this work and pointing out the importance for mechanistic understanding of DNA methylation maintenance. We have addressed the Reviewer's concern on sample preparation and the cellular assays as outlined below.

1) Given that the CUT&TAG methodology is inherently biased towards open chromatin regions such as promoters and enhancers, which often exhibit high CpG density, appropriate controls are required to support the statement that "DNMT1 preferentially binds to sites exhibiting high CG density." Please show the read depth of the and also include a negative control such as the 1KO-mESCs without transfection of exogenous DNMT1 proteins, in relevant figures (e.g., Figs. 3F and 4H).

The Reviewer's point is well taken. Following the Reviewer's suggestion, we have included the following controls to ensure the biological relevance of the observation. (i) Spike-in Normalization: To rigorously account for technical variation and accessibility bias, we implemented a spike-in normalization strategy using human HEK293 cells expressing a 3xFlag-tagged chromatin factor as an internal standard. This allowed for precise

quantification and confirmed that DNMT1 occupancy at CGIs significantly exceeds both the 1KO baseline and the background signal at other open chromatin regions, providing conclusive evidence for the enzyme's preferential recruitment to these loci. (ii) Sequencing Depth: All current replicates were sequenced to a high depth, ranging from ~26.1M to ~32.9M reads (WT: 32.9M, 30.3M; Mut^{KQ}: 26.1M, 27.1M), providing the resolution necessary for high-confidence peak calling and quantitative comparison. (iii) Negative Control: We performed DNMT1 CUT&Tag in 1KO-mESCs (the parental line lacking exogenous DNMT1) and WT cells, confirming that the DNMT1-FLAG signal is not non-specific background noise (Figure R9). This control clearly demonstrates that the enrichment observed at CpG islands (CGIs) is dependent on the presence of DNMT1. This data has been included as Fig. 3f and Supplementary Fig. 6 in the revised manuscript.

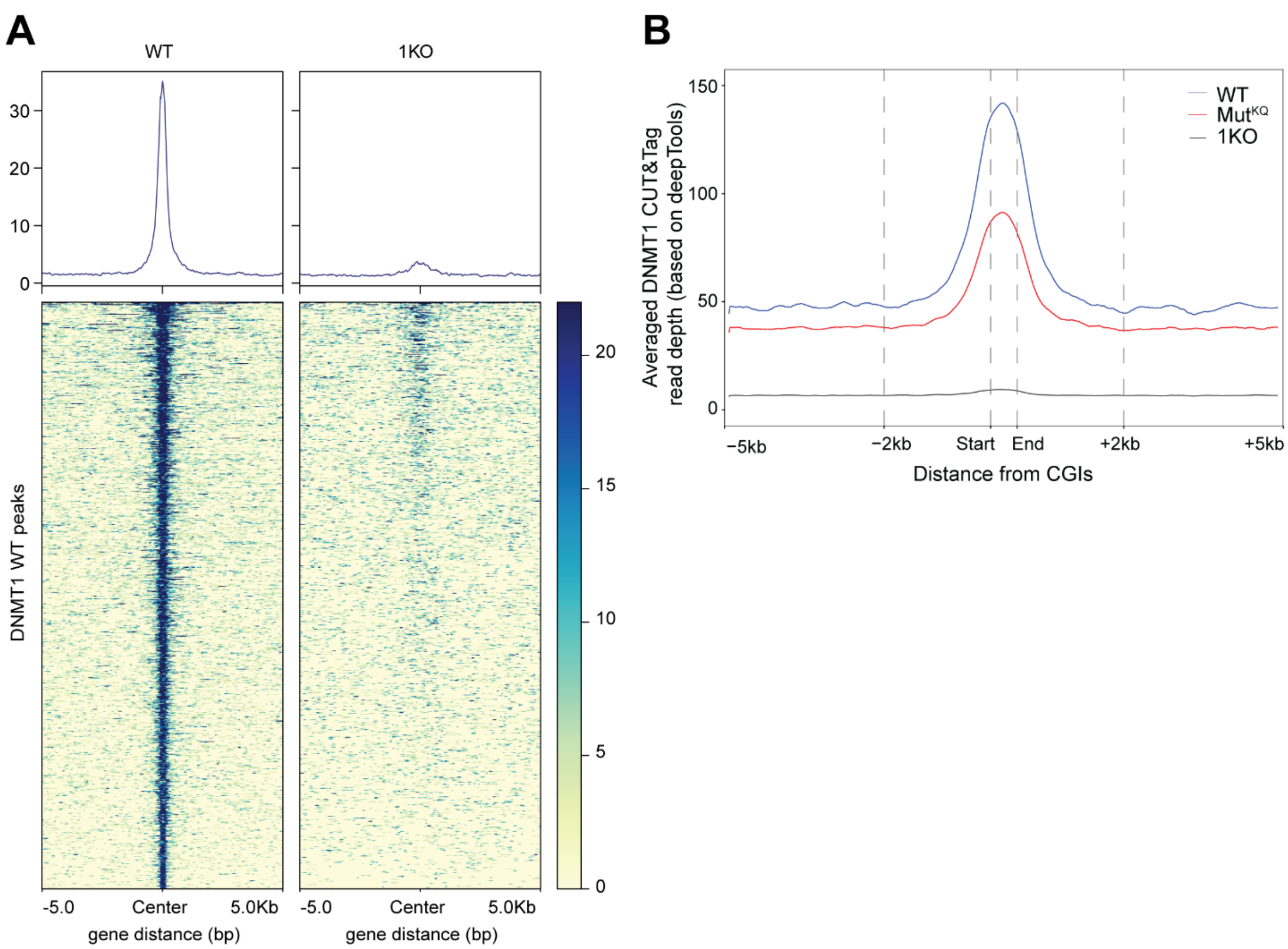

Figure R9. DNMT1 signal at WT peaks and CpG islands in 1KO, WT and Mut^{KQ} cells. (A) Heatmap of DNMT1 CUT&Tag signal across WT DNMT1 peaks in WT and 1KO cells, generated with deepTools computeMatrix/plotHeatmap, centered on DNMT1 peak centers ± 5 kb. Rows represent individual peak regions; color scale indicates normalized DNMT1 signal, shared between WT and 1KO. (B) Metagene plot comparing the 1KO, WT, and Mut^{KQ} DNMT1 binding over a window of ±5 kb from CGIs, with a bin size of 10 bp.

Vertical dashed lines denote key genomic landmarks, namely, CGIs and CGI shores (defined to be ±2 kb from CGIs). CGIs coordinates were obtained from the mm10 genome assembly and extended by ±5 kb to include flanking regions. Each CGI, along with its flanking regions, was scaled to a uniform length (500bp) to facilitate comparative analysis.

2) The gain of DNA methylation in CGIs per se, and in the neighboring CGI shores, appears to be very modest (Fig 4H). Therefore, the statement that the authors observed "most pronounced increases of 5mC at the CGI shore regions", seems overstated and should be revised to a more accurate description, such as "there was a modest increase of 5mC at the CGI shore regions" to give a fair description of the results.

We agree with the reviewer that while the most obvious increase of DNA methylation occurred at CGI shores (Fig. 4e), the absolute increase was modest in scale (Fig. 4h). Following the Reviewer's suggestion, we have rephrased the statement as "**calculation for the methylation level differences in cells with the Mut^{KQ} versus WT DNMT1 revealed rather modest increases of 5mC across the genome, with the most obvious changes occurring at the CGI shore regions (with CpG site-by-site methylation difference—or delta value—of 3.33%) and CGIs (with a delta value of 2.94%)**" in the revised manuscript.

To clarify our observation, we have included additional analysis of DNA methylation among various genomic elements (Fig. R2B). We observed that whereas the non-CGI promoters and H3K9me3+ regions remain methylated in a near-saturation level between WT and Mut^{KQ} cells, the median methylation within CGIs shifted from 2% in WT to 3% in Mut^{KQ}, and increased from 55% to 60% in CGI shores (both with $p < 0.001$, Wilcoxon rank-sum test) (Figure R2B, top panel). The data supports the notion that the CGI and shores represent the regions of highest vulnerability to the loss of CXXC-mediated safeguarding. This data has been included as Supplementary Fig. 9a in the revised manuscript.

3) Related to the point above, what is the median methylation level within CGIs (not including the shores) in cells expressing WT or mutant DNMT1? I Is there a statistically significant difference between these conditions?

In our analysis of CGIs specifically (excluding shore regions) as above (Fig. R2B, top panel), the median methylation level shifted from 2% in WT to 3% in Mut^{KQ} ($p < 0.001$). While the absolute numerical shift is 1%, this represents a 1.5-fold increase over the basal level. Crucially, this signifies a statistically significant departure from the highly robust, near-zero methylation state typically maintained at CGIs.

4) The weak gain of DNA methylation in CGI shores may simply reflect a genome-wide, modest increase in DNA methylation. To address this possibility, the authors should

quantify DNA methylation level in well-characterized heterochromatic regions, such as H3K9me3-enriched domains and specific ERV or LINE families.

Following the Reviewer (and Reviewer 1)'s suggestion, we quantified mCpG levels in H3K9me3-enriched heterochromatic domains (using published ChIP-seq data; GSE90893) (Fig. R2B, top panel) and major repetitive element families (e.g., LINE-1 and ERVs) (Fig. R2B, bottom panel). Our analysis shows that while Mut^{KQ} cells do exhibit a modest global increase in methylation, the biological impact is most evident in CpG-rich regions like CGIs and shores. In heterochromatic and repetitive regions where baseline methylation is already high (~90%), additional gains are largely constrained by a saturation effect. In contrast, the most significant de novo methylation events occur at CpG-rich loci that are normally safeguarded by the CXXC domain. This data has been included as Supplementary Fig. 9a in the revised manuscript.

5) As the authors propose a functional link between DNA methylation gain around CGIs and altered developmental gene expression during ESC differentiation, it is important to further define DNA methylation changes according to the transcriptional and/or epigenetic states in CGIs. Do the authors see a difference in the propensity for methylation gain in different types of CGIs, such as active vs repressed, and/or bound or not bound by the polycomb group (PcG) of proteins?

We thank the reviewer for this insightful suggestion. To further define the propensity for DNA methylation gain, we re-analyzed our WGBS data by stratifying CGIs based on their genomic context and epigenetic status.

1. Genomic Context (Orphan vs. Promoter CGIs): We categorized CGIs as either Promoter CGIs (overlapping with annotated TSS +/- 2kb) or Orphan CGIs (distal/intergenic or intergenic) (Fig. R10A). While both groups exhibited statistically significant methylation gains in the Mut^{KQ} line ($p < 0.0001$), Orphan CGIs showed a markedly higher propensity for methylation gain, with the median methylation level increasing from 4.5% in WT to 8.6% in Mut^{KQ} (a median delta of 2.9%), compared to the 1.5% to 2.8% shift observed at Promoter CGIs (Fig. R10B).

2. Epigenetic State (Active vs. PcG-Repressed): To directly address the role of Polycomb-mediated repression, we stratified CGIs by their H3K27me3 status (using our newly performed CUT&Tag data) (Fig. R10C). We found that H3K27me3-positive (PcG-bound/repressed) CGIs are significantly more susceptible to methylation gain in the absence of CXXC-mediated protection. These repressed regions showed a median gain of 2.1%, double the 1.0% gain observed at H3K27me3-negative (active) CGIs (Fig. R10D).

Taken together, these data demonstrate that PcG-marked repressed CGIs and distal Orphan CGIs have a higher propensity for methylation gain when the CXXC-mediated

protection of DNMT1 is compromised. These data have been included as Fig. 5d-g in the revised manuscript.

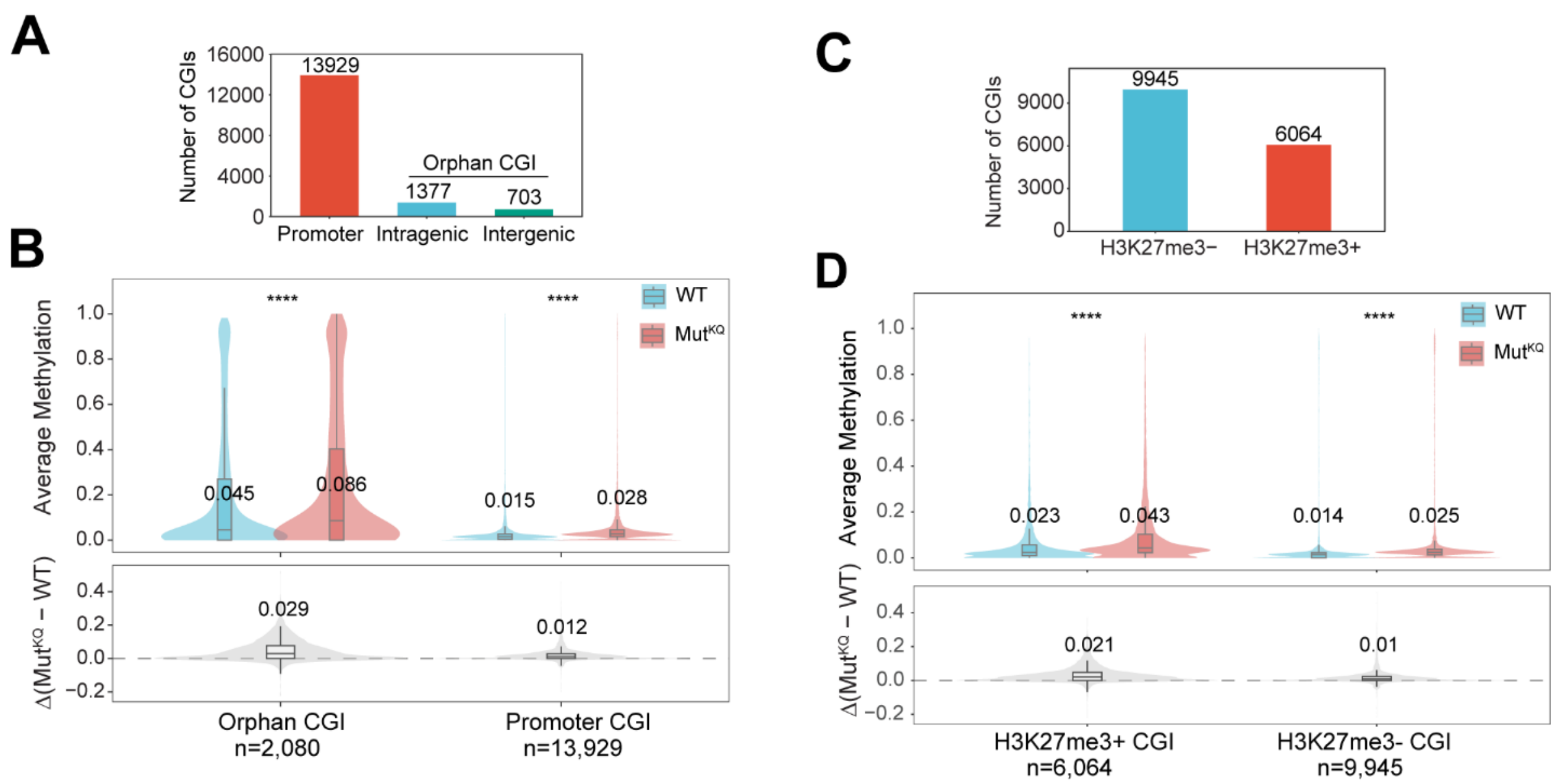

Figure R10. DNA methylation gains in CGIs. (A) Bar chart displaying the distribution and absolute counts of annotated CpG islands (CGIs) categorized as either Promoter CGIs (TSS ± 2 kb) or Orphan CGIs (subdivided into intragenic and intergenic elements). (B) Top panel: Violin plots illustrating average DNA methylation levels in WT (blue) and CXXC-deficient Mut^{KQ} (red) cells across Orphan vs. Promoter CGIs. Numbers represent population medians. Bottom panel: Expanded distribution of the direct methylation shift (Delta = Mut^{KQ} - WT) per individual CGI locus, overlaid with central boxplots. (C) Distribution and relative percentage of total CGIs stratified by Polycomb chromatin status into H3K27me3- (active/unmarked) and H3K27me3+ (PcG-repressed) cohorts. (D) Top panel: Violin plots comparing baseline and mutant fractional methylation distributions between H3K27me3+ and H3K27me3- CGIs. Bottom panel: Direct locus-by-locus methylation variance (Delta = Mut^{KQ} - WT). Total island counts (n) are indicated beneath all functional groups. Statistical significance was evaluated using a two-tailed paired Wilcoxon signed-rank test (****$p < 0.0001$).

6) Changes in the expression level of developmental genes shown in Fig 5h should be accompanied by more detailed information. Gata6, Nkx2-5 and Eomes are typical targets of polycomb group (PcG) of proteins, and therefore it is expected that the expression of such genes will mainly depend on binding of PcG factors at the CGI regions of those genes. The authors should check H3K27me3 levels and/or binding of PRC1/2 (polycomb repressive complexes 1 or 2, such as RING1B, EZH2 and SUZ12) factors at those CGIs, to describe whether the observed perturbation in the transcription levels of these genes is related to changes in local chromatin regulation by PcG proteins.

We thank the Reviewer for raising this excellent point. Following the Reviewer's suggestion, we performed H3K27me3 CUT&Tag in WT and Mut^{KQ} cells and examined the signal genome-wide across CGIs (Fig. R11A). Compared with WT cells, the H3K27me3 signal in Mut^{KQ} cells was reduced across genome-wide CGIs, with significant decrease observed for those CGIs overlapping with the promoters (TSS ± 2 kb) of the developmental gene panel (including Gata6, Nkx2-5, and Eomes) (Fig. R11B-D). Therefore, this data links the observed transcriptional downregulation of the developmental genes to the change in local chromatin regulation by PcG proteins. The concurrent gain of DNA methylation and loss of H3K27me3 in the corresponding regions further reinforces the previous notion that DNA methylation and H3K27me3 marks are mutually exclusive at CGIs[14-17]. We have included the data in the revised manuscript as Supplementary Fig.10.

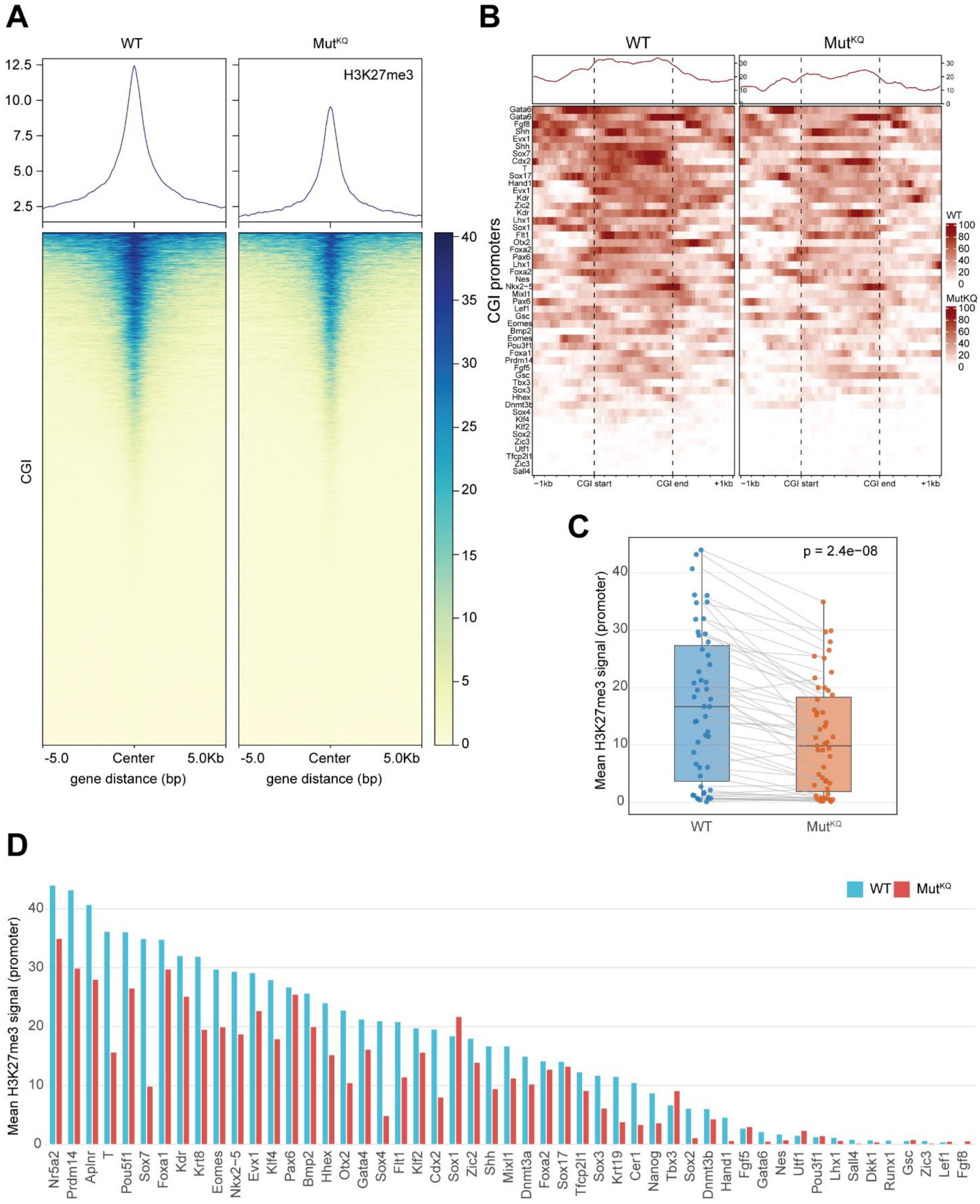
A
WT
Mut^KQ
H3K27me3
12.5
10.0
7.5
5.0
2.5
CGI
40
35
30
25
20
15
10
5
0
-5.0
Center
5.0Kb
gene distance (bp)
B
WT
Mut^KQ
CGI promoters
Gata6
Gata6
Fgf8
Shh
Evx1
Shh
Sox7
Cdx2
T
Sox17
Hand1
Evx1
Kdr
Zic2
Kdr
Lhx1
Sox1
Flt1
Otx2
Foxa2
Pax6
Lhx1
Foxa2
Nes
Nkx2−5
Mixl1
Pax6
Lef1
Gsc
Eomes
Bmp2
Eomes
Pou3f1
Foxa1
Prdm14
Fgf5
Gsc
Tbx3
Sox3
Hhex
Dnmt3b
Sox4
Klf4
Klf2
Sox2
Zic3
Utf1
Tfcp2l1
Zic3
Sall4
−1kb
CGI start
CGI end
+1kb
WT
MutKQ
100
80
60
40
20
0
C
p = 2.4e−08
Mean H3K27me3 signal (promoter)
40
30
20
10
0
WT
Mut^KQ
D
WT
Mut^KQ
Mean H3K27me3 signal (promoter)
40
30
20
10
0
Nr5a2
Prdm14
Aplnr
T
Pou5f1
Sox7
Foxa1
Kdr
Krt8
Eomes
Nkx2−5
Evx1
Klf4
Pax6
Bmp2
Hhex
Otx2
Gata4
Sox4
Flt1
Klf2
Cdx2
Sox1
Zic2
Shh
Mixl1
Dnmt3a
Foxa2
Sox17
Tfcp2l1
Sox3
Krt19
Cer1
Nanog
Tbx3
Sox2
Dnmt3b
Hand1
Fgf5
Gata6
Nes
Utf1
Pou3f1
Lhx1
Sall4
Dkk1
Runx1
Gsc
Zic3
Lef1
Fgf8

Figure R11. H3K27me3 signal at CGIs and gene promoters in WT and Mut^{KQ} cells. (A) Heatmap of H3K27me3 CUT&Tag signal across all CGIs genome-wide in WT and Mut^{KQ} cells, generated with deepTools computeMatrix/plotHeatmap, centered on CGIs ± 5 kb. Rows represent individual CGIs; color scale indicates normalized H3K27me3 signal, shared between WT and Mut^{KQ}. (B) Heatmap of H3K27me3 CUT&Tag signal restricted to the CGIs overlapping with the promoters (TSS ± 2 kb) of the developmental gene panel Fig. 5h, in WT and Mut^{KQ}. Each row is one CGI, generated with EnrichedHeatmap::normalizeToMatrix, flanked by 1 kb on either side of the CGI boundaries (dashed lines: CGI start/end). Rows are ranked by WT signal intensity, high to low; gene names are indicated at left. Color scale shared between WT and Mut^{KQ}. (C) Boxplot summarizing mean H3K27me3 signal at the promoter (TSS ± 2 kb) of each gene in (B), comparing WT and Mut^{KQ}. Each point represents one gene's promoter; grey lines connect paired WT–Mut^{KQ} values for the same gene. H3K27me3 signal was significantly reduced in Mut^{KQ} (paired Wilcoxon signed-rank test, $p = 2.4 \times 10^{-8}$). (D) Bar plot of mean H3K27me3 signal at the promoter of each individual gene in the panel, comparing WT (blue) and Mut^{KQ} (red), sorted by WT signal from high to low. Most genes show reduced H3K27me3 in Mut^{KQ} relative to WT.

7) On page 6, lines 4–6 under "Structural details of the DNMT1 CXXC domain–CpG interaction at the dyad", the sentence starting with "rather than …" is difficult to understand and would benefit from clarification or rephrasing.

Following the Reviewer's suggestion, we have rephrased the statement as "**The fact that the CXXC domain failed to interact with the rest of the CpG sites is presumably due to the reduced accessibility of those sites**".

8) In Figure 2d–e and Supplementary Figures 2j, 2l, and 4e, the regions highlighted by the color coding are not clearly explained. It would be helpful to explicitly indicate which regions are being shown, for example by following the labeling style used in Figure 2a.

We thank the Reviewer for pointing out the issue. We have clarified these figures in the revised manuscript.

9) Please show the purity of each histone protein and the histone octamer by SDS-PAGE, and also the assembled nucleosomes by native PAGE.

We have included the SDS-PAGE images of the histone proteins and histone octamers in Supplementary Fig. 1a, and the native PAGE of assembled nucleosomes in Fig. 2f and Supplementary Fig 1b, 2m, 5a,f-h in the revised manuscript.

References

1. Ishiyama, S. *et al.* Structure of the Dnmt1 Reader Module Complexed with a Unique Two-Mono-Ubiquitin Mark on Histone H3 Reveals the Basis for DNA Methylation Maintenance. *Mol Cell* **68**, 350-360 e357 (2017).
2. Nishiyama, A. *et al.* Uhrf1-dependent H3K23 ubiquitylation couples maintenance DNA methylation and replication. *Nature* **502**, 249-253 (2013).
3. Qin, W. *et al.* DNA methylation requires a DNMT1 ubiquitin interacting motif (UIM) and histone ubiquitination. *Cell research* **25**, 911-929 (2015).
4. Bronner, C., Alhosin, M., Hamiche, A. & Mousli, M. Coordinated Dialogue between UHRF1 and DNMT1 to Ensure Faithful Inheritance of Methylated DNA Patterns. *Genes* **10** (2019).
5. Yamaguchi, K. *et al.* Non-canonical functions of UHRF1 maintain DNA methylation homeostasis in cancer cells. *Nature communications* **15**, 2960 (2024).
6. Ren, W. *et al.* Direct readout of heterochromatic H3K9me3 regulates DNMT1-mediated maintenance DNA methylation. *Proc Natl Acad Sci U S A* **117**, 18439-18447 (2020).
7. Medina-Cano, D. *et al.* Rapid and robust directed differentiation of mouse epiblast stem cells into definitive endoderm and forebrain organoids. *Development* **149** (2022).
8. Li, J. *et al.* The sotos syndrome gene Nsd1 safeguards developmental gene enhancers poised for transcription by maintaining the precise deposition of histone methylation. *J Biol Chem* **301**, 108423 (2025).
9. Tovy, A. *et al.* p53 is essential for DNA methylation homeostasis in naive embryonic stem cells, and its loss promotes clonal heterogeneity. *Genes Dev* **31**, 959-972 (2017).
10. Vann, K.R. *et al.* Structure-function relationship of ASH1L and histone H3K36 and H3K4 methylation. *Nature communications* **16**, 2235 (2025).
11. Bostick, M. *et al.* UHRF1 plays a role in maintaining DNA methylation in mammalian cells. *Science* **317**, 1760-1764 (2007).
12. Sharif, J. *et al.* The SRA protein Np95 mediates epigenetic inheritance by recruiting Dnmt1 to methylated DNA. *Nature* **450**, 908-912 (2007).
13. Liddicoat, B.J. *et al.* SUMOylation enhances DNMT1 function to repress mega-intergenic RNAs and viral mimicry. *Nat Genet* **58**, 2000-2012 (2026).
14. Brinkman, A.B. *et al.* Sequential ChIP-bisulfite sequencing enables direct genome-scale investigation of chromatin and DNA methylation cross-talk. *Genome research* **22**, 1128-1138 (2012).
15. Lindroth, A.M. *et al.* Antagonism between DNA and H3K27 methylation at the imprinted Rasgrf1 locus. *PLoS genetics* **4**, e1000145 (2008).
16. Reddington, J.P. *et al.* Redistribution of H3K27me3 upon DNA hypomethylation results in de-repression of Polycomb target genes. *Genome biology* **14**, R25 (2013).
17. Wu, H. *et al.* Dnmt3a-dependent nonpromoter DNA methylation facilitates transcription of neurogenic genes. *Science* **329**, 444-448 (2010).
