# Supplementary material for: The DNMT1 CXXC domain senses CpG islands for DNA methylation inhibition
Source: Nat Commun. 2026 Sep 18;17:10970. doi: 10.1038/s41467-026-77872-8 (PMC13683344; doi:10.1038/s41467-026-77872-8)
Supplement: Supplementary file 3 [file 41467_2026_77872_MOESM1_ESM.pdf]

Supplementary information for

The DNMT1 CXXC domain senses CpG islands for DNA methylation inhibition

Jian Fang[1,#], Bo Pan[2,3,#], Wendan Ren[1,#], Jiuwei Lu[1], Ting Zhao[4], Yiran Guo[2], Ling Cai[3,5], Yinsheng Wang[4,6], Gang Greg Wang[2,3,5,*], Jikui Song[1,*]

[1]Department of Biochemistry, University of California, Riverside, California, USA

[2]Department of Pharmacology and Cancer Biology, Duke University School of Medicine, Durham, NC 27710, USA

[3]Department of Pathology, Duke University School of Medicine, Durham, NC 27710, USA

[4]Environmental Toxicology Graduate Program, University of California, Riverside, CA 92521, USA

[5]Duke Cancer Institute, Duke University School of Medicine, Durham, NC 27710, USA

[6]Department of Chemistry, University of California, Riverside, CA 92521, USA

[#]These authors contributed equally to this work

*Correspondence: greg.wang@duke.edu; jikui.song@ucr.edu

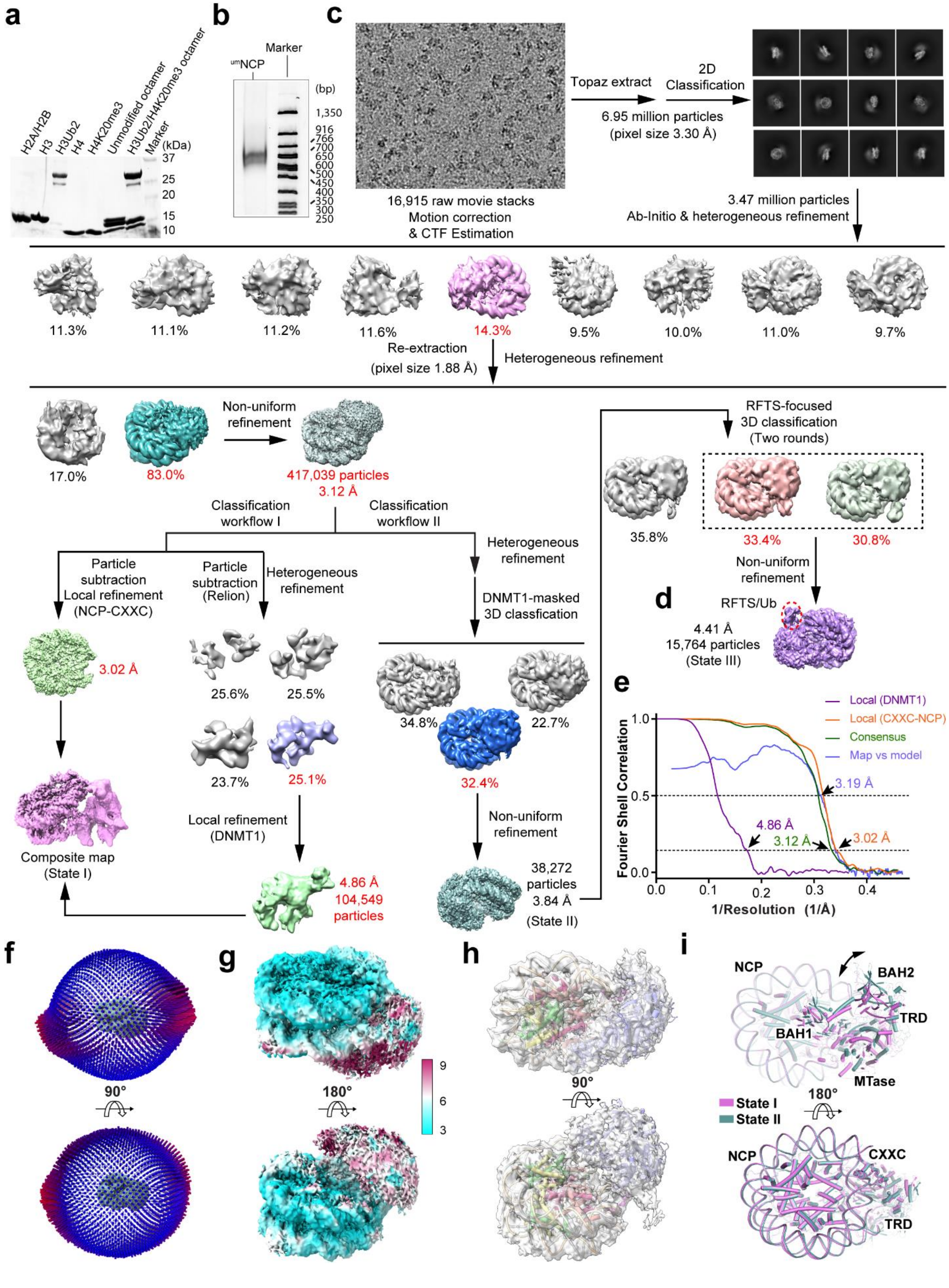

a
H2A/H2B
H3
H3Ub2
H4
H4K20me3
Unmodified octamer
H3Ub2/H4K20me3 octamer
Marker
(kDa)
37
25
20
15
10
b
umNCP
Marker
(bp)
1,350
916
766
700
650
600
500
450
400
350
300
250
c
16,915 raw movie stacks
Motion correction
& CTF Estimation
Topaz extract
6.95 million particles
(pixel size 3.30 Å)
2D
Classification
3.47 million particles
Ab-Initio & heterogeneous refinement
11.3%
11.1%
11.2%
11.6%
14.3%
9.5%
10.0%
11.0%
9.7%
Re-extraction
(pixel size 1.88 Å)
Heterogeneous refinement
17.0%
83.0%
Non-uniform
refinement
417,039 particles
3.12 Å
Classification
workflow I
Classification
workflow II
Particle
subtraction
Local refinement
(NCP-CXXC)
3.02 Å
Particle
subtraction
(Relion)
Heterogeneous
refinement
25.6%
25.5%
23.7%
25.1%
Local refinement
(DNMT1)
4.86 Å
104,549
particles
Composite map
(State I)
Heterogeneous
refinement
DNMT1-masked
3D classfication
34.8%
22.7%
32.4%
Non-uniform
refinement
38,272
particles
3.84 Å
(State II)
RFTS-focused
3D classification
(Two rounds)
35.8%
33.4%
30.8%
Non-uniform
refinement
d
RFTS/Ub
4.41 Å
15,764 particles
(State III)
e
Local (DNMT1)
Local (CXXC-NCP)
Consensus
Map vs model
Fourier Shell Correlation
1/Resolution (1/Å)
3.19 Å
4.86 Å
3.12 Å
3.02 Å
f
90°
g
180°
9
6
3
h
90°
i
NCP
BAH2
TRD
BAH1
MTase
State I
State II
180°
NCP
CXXC
TRD

Supplementary Fig. 1. Cryo-EM data processing of the DNMT1-^{um}NCP complex. a SDS-PAGE images of histone proteins used in this study. **b** Reconstituted ^{um}NCP on native PAGE. **c** Workflow of the cryo-EM data processing of the DNMT1-^{um}NCP complex. The percentage of the particles selected for further analysis is labeled red. **d** A subset of the DNMT1-^{um}NCP particles, after non-uniform refinement, led to identification of the density corresponding to the RFTS/H3Ub2 component (circled). **e-g** FSC curves of overall and local maps as a function of resolution (e), Angular distribution (f) and local resolution map (g) for State I DNMT1-^{um}NCP complex. **h** Two orthogonal views of the transparent density map (light grey) for State-II DNMT1-^{um}NCP complex overlaid with the corresponding structural model. **i** Structural overlay between State-I and State-II DNMT1-^{um}NCP complex in two opposite views. The subtle conformational swing of the DNMT1 BAH1-BAH2-MTase region between the two states is indicated by arrow. Source data are provided with this paper.

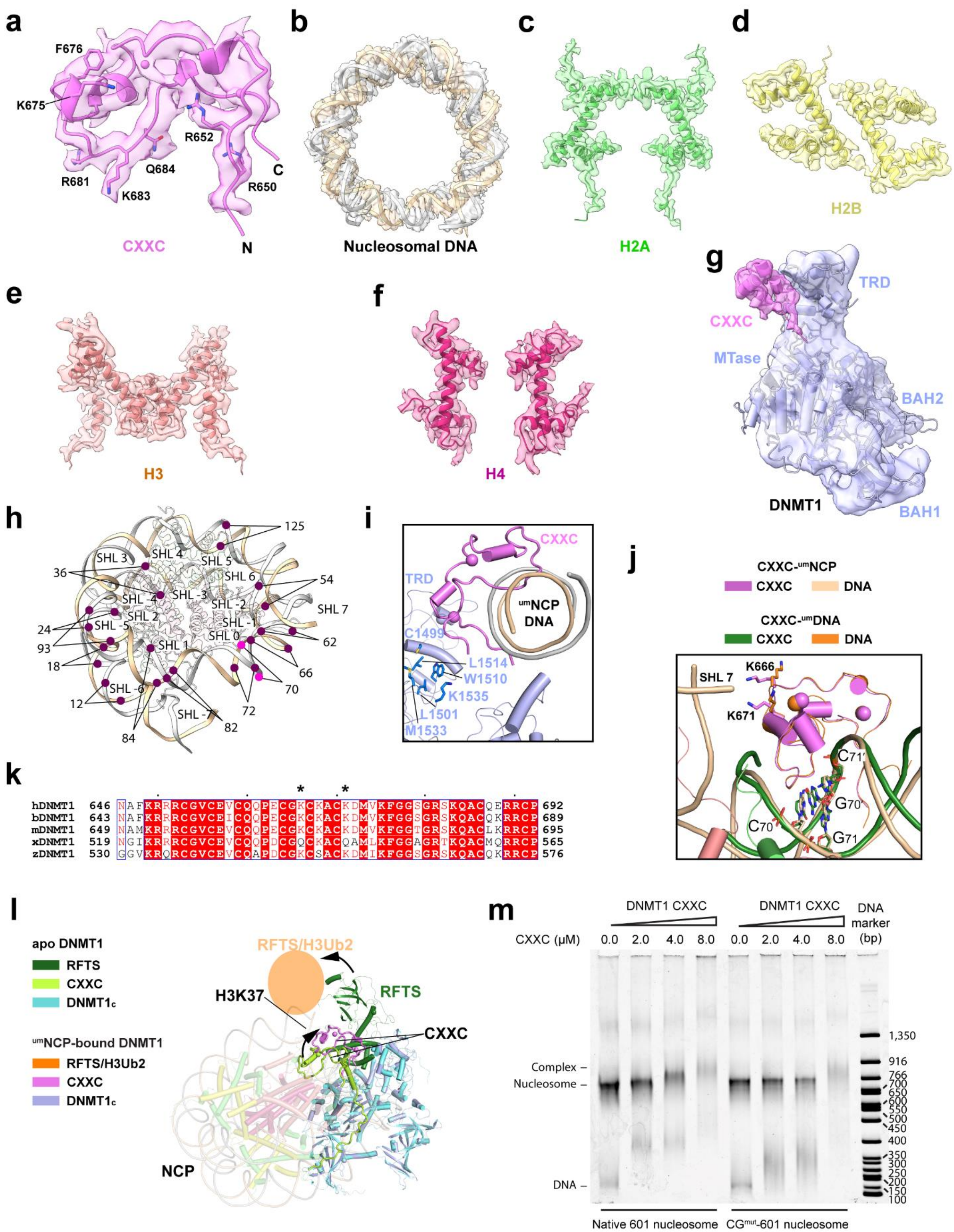
a
F676
K675
R652
Q684
R681
K683
R650
C
N
CXXC
b
Nucleosomal DNA
c
H2A
d
H2B
e
H3
f
H4
g
CXXC
TRD
MTase
BAH2
DNMT1
BAH1
h
125
SHL 3
SHL 4
SHL 5
SHL 6
36
54
SHL -4
SHL -3
SHL 2
SHL -2
SHL 7
24
SHL -5
SHL -1
93
SHL 0
62
18
SHL 1
66
70
12
SHL -6
72
SHL -7
82
84
i
CXXC
TRD
umNCP DNA
C1499
L1514
W1510
K1535
L1501
M1533
j
CXXC-umNCP
CXXC
DNA
CXXC-umDNA
CXXC
DNA
SHL 7
K666
K671
C71'
G70'
C70
G71
k
hDNMT1 646 NAFKRRRCGVCEVCQQPECGKCKACKDMVKFGGSGRSKQACQERRCP 692
bDNMT1 643 NAFKRRRCGVCEICQQPECGKCKACKDMVKFGGSGRSKQACQKRRCP 689
mDNMT1 649 NAMKRRRCGVCEVCQQPECGKCKACKDMVKFGGTGRSKQACLKRRCP 695
xDNMT1 519 NGIKRRRCGVCEVCQQPDCGQCKACQAMLKFGGAGRTKQACMQRRCP 565
zDNMT1 530 GGVKRQRCGVCEVCQAPDCGKCSACKDMIKFGGSGRSKQACQKRRCP 576
l
apo DNMT1
RFTS
CXXC
DNMT1c
umNCP-bound DNMT1
RFTS/H3Ub2
CXXC
DNMT1c
RFTS/H3Ub2
RFTS
H3K37
CXXC
NCP
m
DNMT1 CXXC
DNMT1 CXXC
DNA marker (bp)
CXXC (μM)
0.0 2.0 4.0 8.0 0.0 2.0 4.0 8.0
Complex –
Nucleosome –
DNA –
1,350
916
766
700
650
600
550
500
450
400
350
300
250
200
150
100
Native 601 nucleosome
CGmut-601 nucleosome

Supplementary Fig. 2. Cryo-EM analysis of the DNMT1-^{um}NCP complex. a-g Density maps and atomic models for the DNMT1 CXXC domain, with select residues shown stick representation (a), the NCP DNA (b), H2A (c), H2B (d), H3 (e), H4 (f) and DNMT1 (g). **h** Schematic view of the distribution of the unmethylated CpG sites in the NCP. The CpG dinucleotides interacting with the DNMT1 CXXC domain are colored in magenta and the rest in dark brown. The position of the cytosine with the CpG site of the upper strand is indicated. **i** Close-up view of the DNA-binding site (stick representation) of DNMT1 TRD, the DNA interaction of which is blocked by the CXXC domain. **j** Structural overlay of the CXXC-CpG region between the DNMT1-^{um}NCP complex and the DNMT1-B form CpG DNA complex (DNMT1-^{um}DNA; PDB 3PTA). **k** Sequence alignment of the CXXC domain of DNMT1 from various species (human: hDNMT1; bovine: bDNMT1; mouse: mDNMT1; xenopus: xDNMT1; zebrafish: zDNMT1). Identical residues are in white font on a red background, and similar residues are in red font. The residues corresponding to hDNMT1 K666 and K671 are marked by dark asterisks on top. **l** Structural overlay between the DNMT1-^{um}NCP complex and apo-form DNMT1$_{351-1600}$ (PDB 4WXX). DNMT1c denotes the DNMT1 fragment (except for the CXXC domain) downstream of the RFTS domain. The positioning of the RFTS/H3Ub2 component in the DNMT1-^{um}NCP complex is indicated by shaded circle in beige. The conformational differences in the CXXC domain and the RFTS domain between the two states of DNMT1 are indicated by arrows. **m** EMSA analysis of the interaction of the DNMT1 CXXC domain with a nucleosome containing native 601 sequence (U-Linker1$^{3hmCG-6CA}$) or a nucleosome variant (CG^{Mut}-601) in which five of the CpG sites near to the dyad, site 62, 66, 70, 72, and 82 as indicated in panel (h), were replaced with non-CG sites (see Methods). The experiment was repeated twice with consistent results. Source data are provided with this paper.

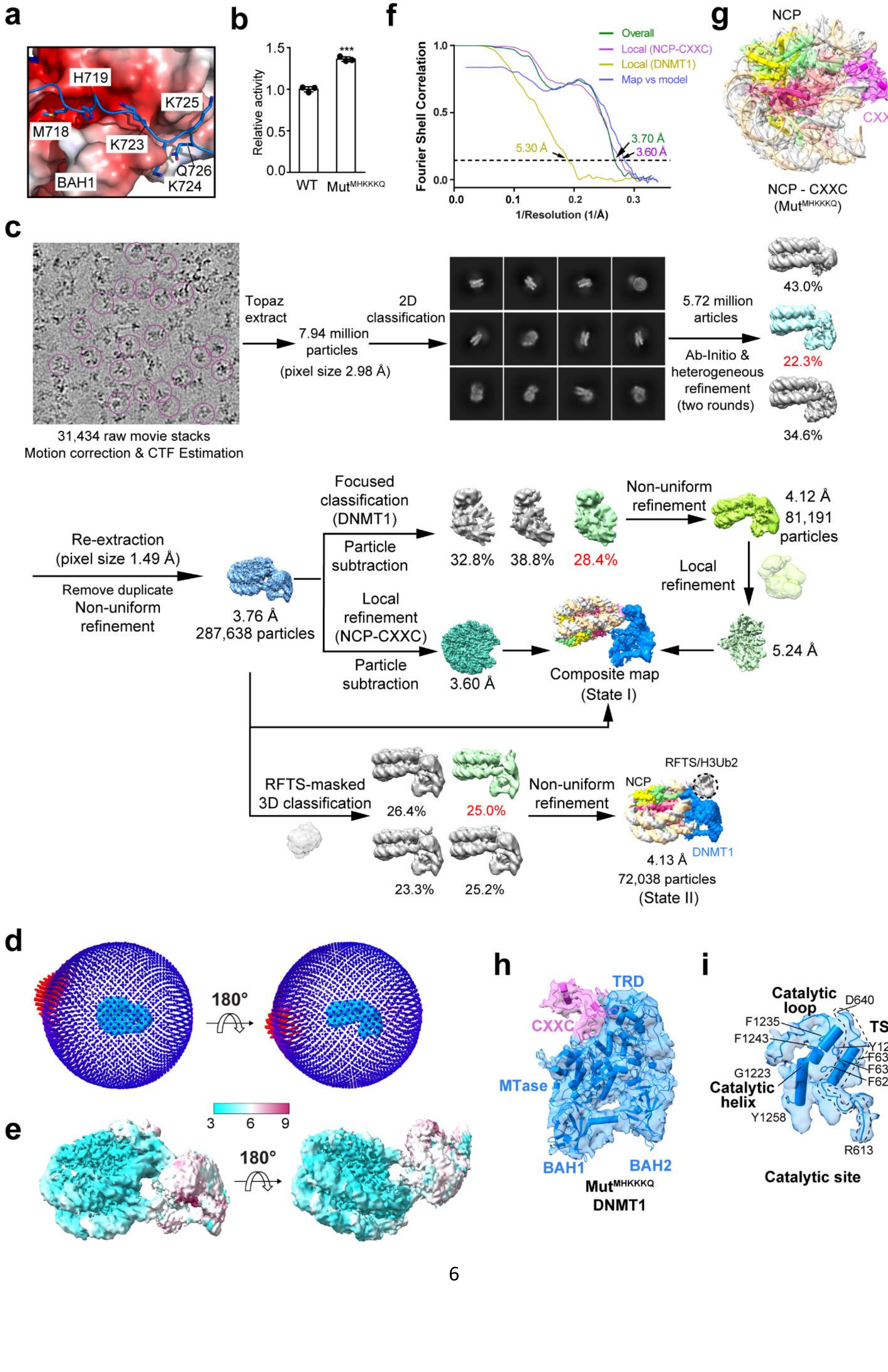
a
H719
K725
M718
K723
Q726
BAH1
K724
b
1.5
1.0
0.5
0.0
Relative activity
***
WT
Mut^MHKKKQ
f
Overall
Local (NCP-CXXC)
Local (DNMT1)
Map vs model
Fourier Shell Correlation
1/Resolution (1/Å)
5.30 Å
3.70 Å
3.60 Å
g
NCP
CXXC
NCP - CXXC
(Mut^MHKKKQ)
c
31,434 raw movie stacks
Motion correction & CTF Estimation
Topaz extract
7.94 million particles
(pixel size 2.98 Å)
2D classification
5.72 million articles
Ab-Initio & heterogeneous refinement (two rounds)
43.0%
22.3%
34.6%
Re-extraction (pixel size 1.49 Å)
Remove duplicate
Non-uniform refinement
3.76 Å
287,638 particles
Focused classification (DNMT1)
Particle subtraction
32.8%
38.8%
28.4%
Non-uniform refinement
4.12 Å
81,191 particles
Local refinement
Local refinement (NCP-CXXC)
Particle subtraction
3.60 Å
Composite map (State I)
5.24 Å
RFTS-masked 3D classification
26.4%
25.0%
23.3%
25.2%
Non-uniform refinement
RFTS/H3Ub2
NCP
DNMT1
4.13 Å
72,038 particles
(State II)
d
180°
e
3
6
9
180°
h
TRD
CXXC
MTase
BAH1
BAH2
Mut^MHKKKQ DNMT1
i
Catalytic loop
D640
TS
F1235
F1243
Y1240
F632
G1223
F631
Catalytic helix
F628
Y1258
R613
Catalytic site

Supplementary Fig. 3. Biochemical and cryo-EM analysis of the Mut^{MHKKKQ} DNMT1-^{um}NCP complex. a Close-up view of the electrostatic surface of the BAH1 domain occupied by a fragment of the autoinhibitory linker. The BAH1-interacting residues of the linker are shown in stick representation. **b** In vitro DNA methylation assays of DNMT1$_{351-1600}$, WT or Mut^{MHKKKQ} on the nucleosome substrate. Two-sided unpaired t-test was used for statistical analysis of WT vs mutant DNMT1$_{351-1600}$. ***p<0.001. Data are mean±s.d. (n=3 biological replicates). **c** Workflow of cryo-EM data processing of Mut^{MHKKKQ} DNMT1-^{um}NCP. The density map used for structural analysis, with the CXXC-NCP component derived from local refinement of 287,638 particles and DNMT1 derived from local refinement of 81,191 particles, denoted as State I. The map state with notable density for the RFTS/H3Ub2 component, indicated by dotted circle, is derived from further classification of the 287,638 particles and denoted as State II. **d-f** Angular distribution (d), and local resolution map (e) and FSC curves for overall and local maps as a function of resolution (f) for Mut^{MHKKKQ} DNMT1-^{um}NCP complex. **g-i** Density maps and atomic models for the DNMT1 CXXC-^{um}NCP component (g), Mut^{MHKKKQ} DNMT1 (h), the region encompassing the catalytic loop, catalytic helix and the toggle switch (TS) of Mut^{MHKKKQ} DNMT1 (i). Note that the catalytic loop, catalytic helix and the toggle switch all assume catalytically inactive conformation, with the bending conformation of the catalytic helix directs the catalytic loop away from potential DNA binding.

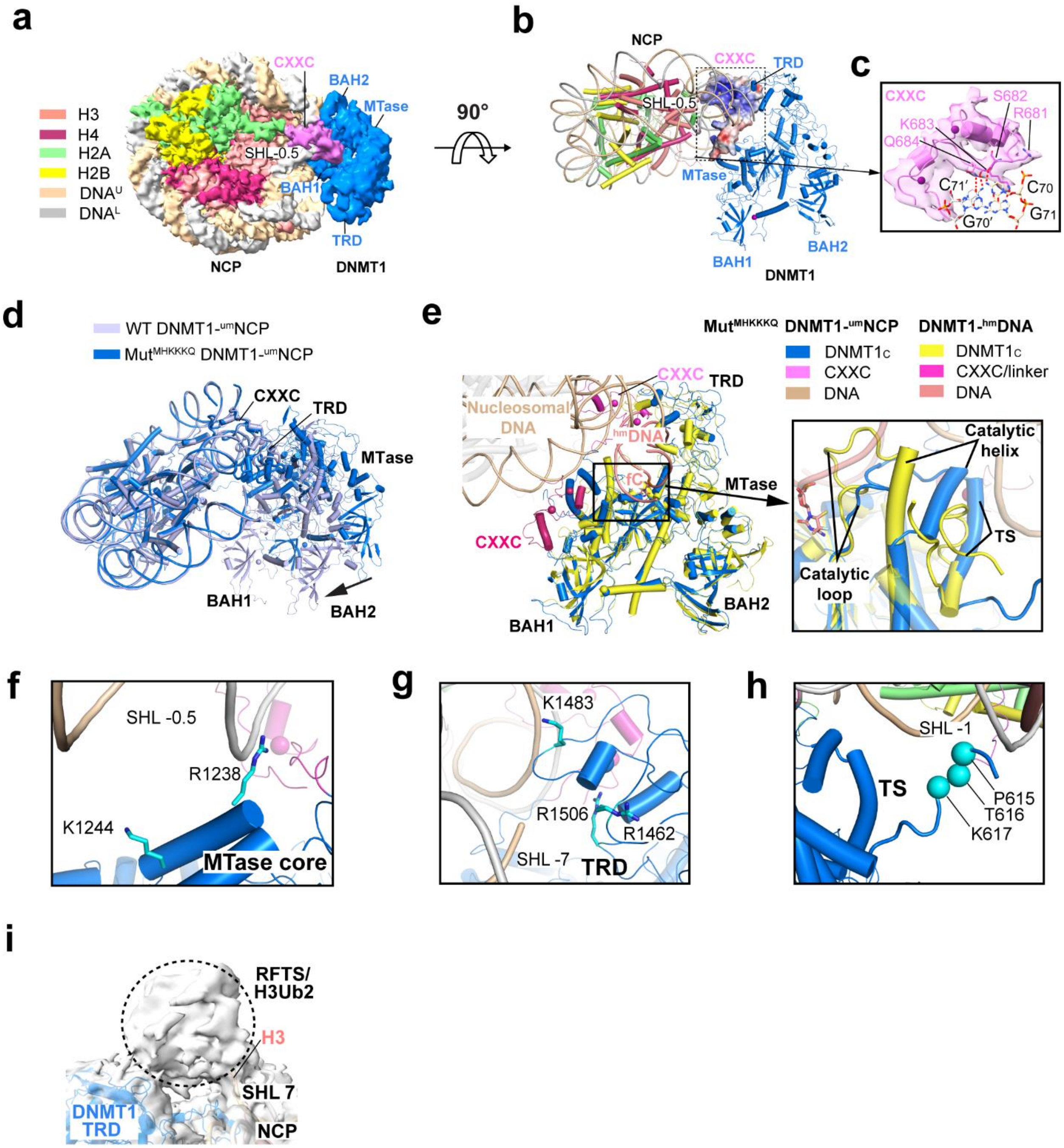

Supplementary Fig. 4. Structural analysis of the Mut^{MHKKKQ} DNMT1-^{um}NCP complex. a Composite density map of the Mut^{MHKKKQ} DNMT1-^{um}NCP complex with the CXXC domain colored in light magenta and the rest of DNMT1 colored in marine blue. **b** Atomic model of Mut^{MHKKKQ} DNMT1-^{um}NCP complex, with individual domains labeled

and the CXXC domain shown as electrostatic surface. **c** Density map and atomic model of the CXXC domain of Mut^{MHKKKQ} DNMT1 CXXC domain interaction between the CXXC domain and the CpG site. The CpG-interacting residues of the CXXC domain are shown in stick representation. The CpG dinucleotides at SHL -0.5 are labeled as C_{70} and G_{71} for the DNA^{U} strand and C_{70}' and G_{71}' for the DNA^{L} strand. shown in the expanded view. The hydrogen bonds are shown as dashed lines. **d** Structural overlay between WT and Mut^{MHKKKQ} DNMT1-^{um}NCP complexes, with the DNA-interacting CXXC domain and TRD labeled. **e** Structural overlay between the autoinhibitory Mut^{MHKKKQ} DNMT1-^{um}NCP complex and the active DNMT1-^{hm}DNA complex (7XI9), with the conformational difference in the catalytic loop, catalytic helix and toggle switch (TS) highlighted in the expanded view. DNMT1c denotes the DNMT1 fragment (except for the CXXC domain) downstream of the RFTS domain. **f-h** Close-up view of the DNA interaction by the DNMT1 MTase core (f), TRD (g) and toggle switch (TS) (h). The DNA-interacting residues are labeled as sticks in (f,g) and spheres in (h). **i** Close-up view of the density map for the unmodeled RFTS-H3Ub2 in the Mut^{MHKKKQ} DNMT1-^{um}NCP complex, positioned between the DNMT1 TRD and the H3 tail-proximal SHL 7 site.

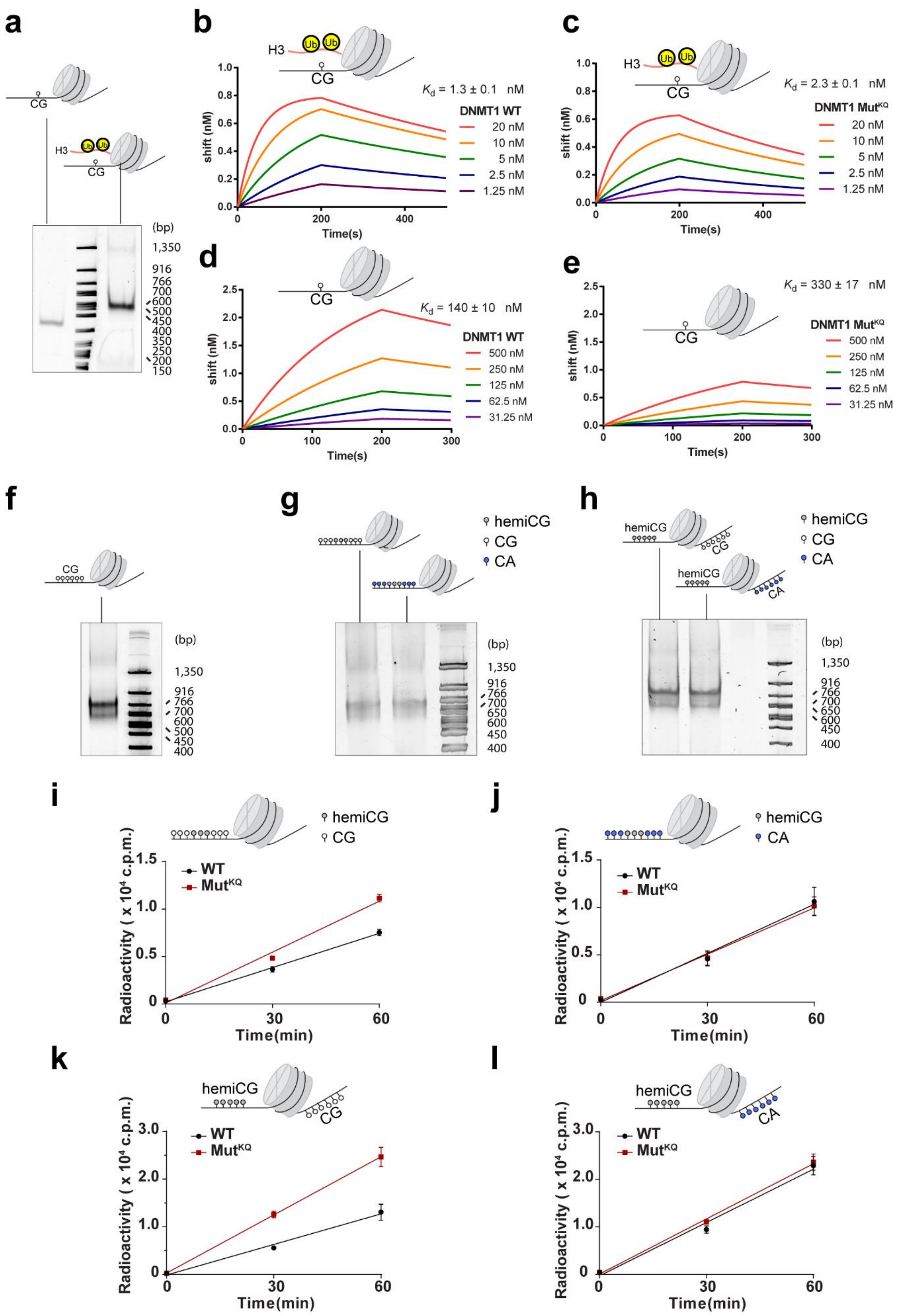

a
b
c
d
e
f
g
h
i
j
k
l
H3
Ub
CG
(bp)
K_d = 1.3 ± 0.1 nM
DNMT1 WT
K_d = 2.3 ± 0.1 nM
DNMT1 Mut[KQ]
K_d = 140 ± 10 nM
K_d = 330 ± 17 nM
shift (nM)
Time(s)
hemiCG
CA
WT
Mut[KQ]
Radioactivity (x 10[4] c.p.m.)
Time(min)

Supplementary Fig. 5. Biochemical and enzymatic analysis of DNMT1 CXXC domain-mediated autoinhibition. a Native gel images for the unmodified and H3Ub2-modified CpG-containing nucleosomes used for BLI assay. **b,c** BLI binding curves for $DNMT1_{351-1600}$, WT (b) or Mut^{KQ} (c), with H3Ub2-modified, CpG-containing nucleosome. Biotinylated nucleosomes were immobilized on streptavidin biosensors, titrated with various concentrations of $DNMT1_{351-1600}$, as indicated. Data are mean ± s.d. (*n* = 2 biological replicates). One representative set of BLI binding data is shown. **d,e** BLI binding curves for $DNMT1_{351-1600}$, WT (d) or Mut^{KQ} (e), with unmodified, CpG-containing nucleosome. Biotinylated nucleosomes were immobilized on streptavidin biosensors, titrated with various concentrations of $DNMT1_{351-1600}$, as indicated. Data are mean ± s.d. (*n* = 2 biological replicates). One representative set of BLI binding data is shown. **f** Native gel images for the unmodified CpG-containing nucleosome ($NucDNA^{CG}$) used for *de novo* DNA methylation assay in Fig. 2g. **g** Native gel images for the nucleosome with one linker containing hemiCG sites flanked by unmodified CpG (U-$Linker1^{3hmCG-6CG}$) and CpA (U-$Linker1^{3hmCG-6CA}$) sites used for Fig. 2h,i. **h** Native gel images for the nucleosome with one linker containing hemiCG sites and the other linker containing unmodified CpG (U-$Linker1^{5hmCG}$-$Linker2^{CG}$) and CpA (U-$Linker1^{5hmCG}$-$Linker2^{CA}$) sites used for Fig. 2j,k. **i,j** DNMT1-mediated methylation of the U-$Linker1^{3hmCG-6CG}$ (i) or U-$Linker1^{3hmCG-6CA}$ (j) nucleosome. **k,l** DNMT1-mediated methylation of the U-$Linker1^{5hmCG}$-$Linker2^{CG}$ (k) or U-$Linker1^{5hmCG}$-$Linker2^{CA}$ (l) nucleosome. Source data are provided with this paper.

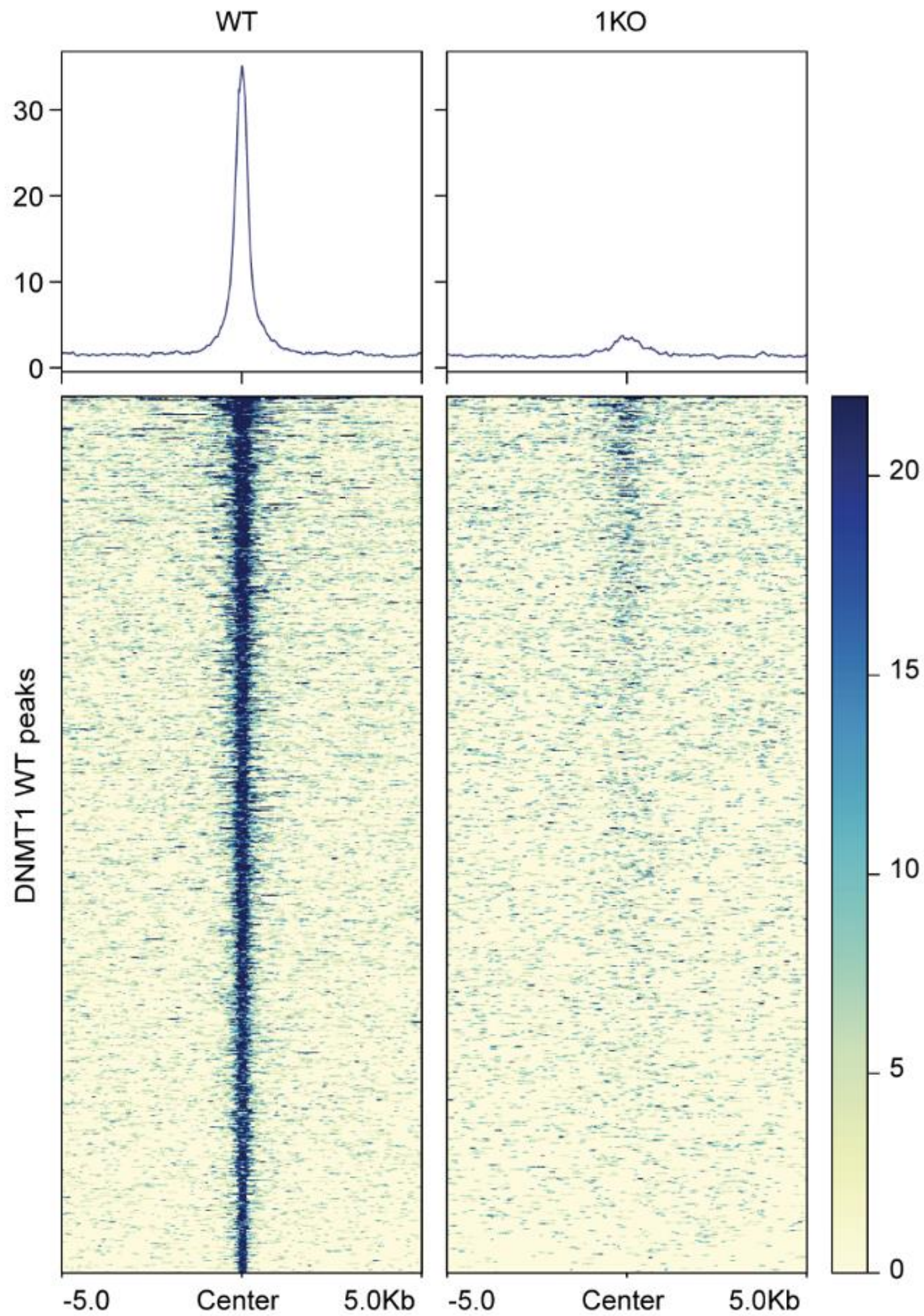

Supplementary Fig. 6. Averaged density (top panel) and heatmap (bottom panel) of DNMT1 CUT&Tag signals across DNMT1 peaks in the *Dnmt1*-knockout mouse embryonic stem cells (1KO-mESCs; right panel) and 1KO-mESCs reconstituted with WT DNMT1 (left panel). CUT&Tag signal heatmaps were generated using the deepTools function of computeMatrix/plotHeatmap, centered on the DNMT1 peak centers ± 5 kb. Rows in the heatmaps represent individual peak regions, while color scale on the right indicates normalized DNMT1 signals. Source data are provided with this paper.

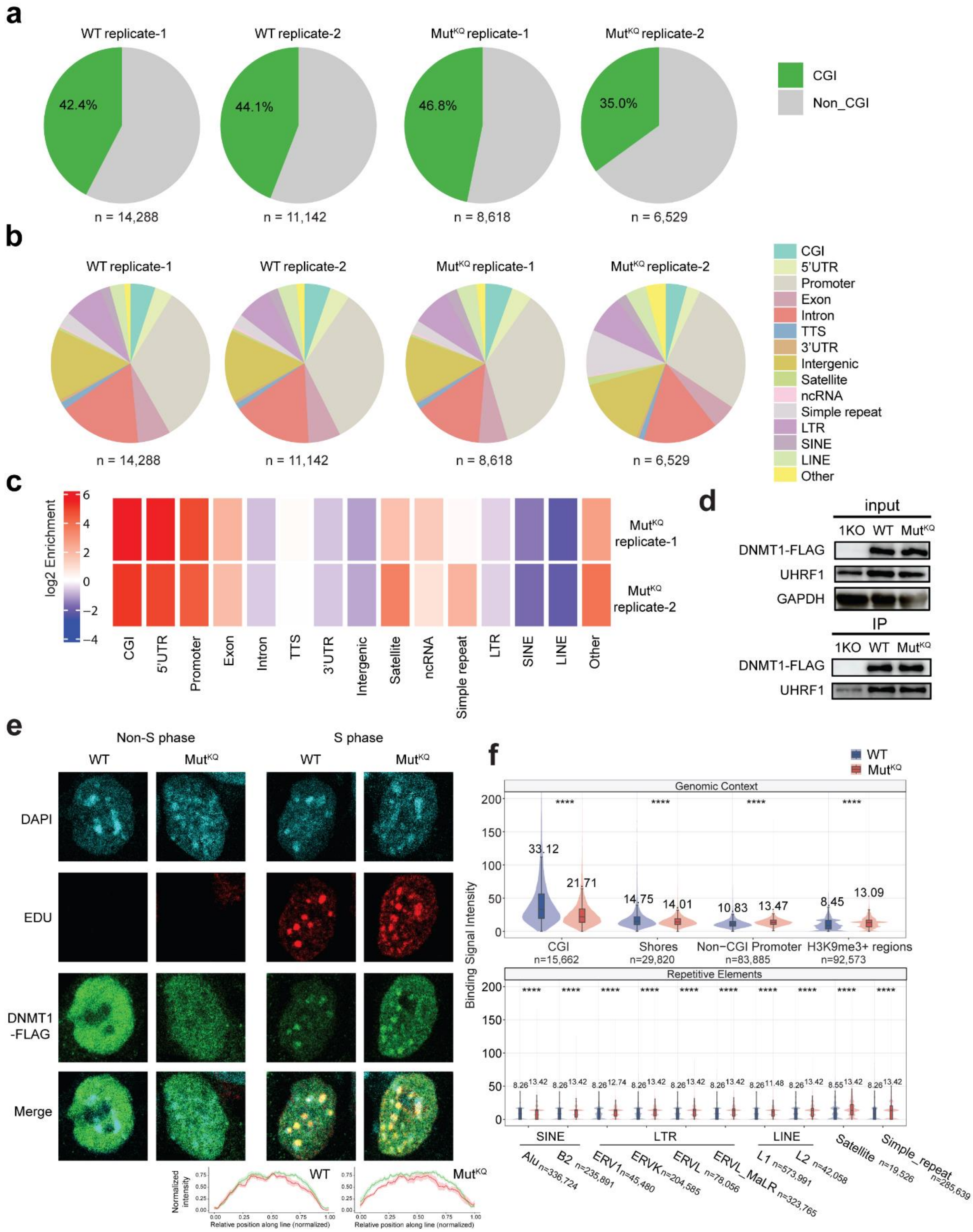
a
WT replicate-1
WT replicate-2
Mut^KQ replicate-1
Mut^KQ replicate-2
42.4%
44.1%
46.8%
35.0%
n = 14,288
n = 11,142
n = 8,618
n = 6,529
CGI
Non_CGI
b
CGI
5'UTR
Promoter
Exon
Intron
TTS
3'UTR
Intergenic
Satellite
ncRNA
Simple repeat
LTR
SINE
LINE
Other
c
log2 Enrichment
Mut^KQ replicate-1
Mut^KQ replicate-2
d
input
IP
1KO WT Mut^KQ
DNMT1-FLAG
UHRF1
GAPDH
e
Non-S phase
S phase
WT
Mut^KQ
DAPI
EDU
DNMT1 -FLAG
Merge
Normalized intensity
Relative position along line (normalized)
f
Genomic Context
Repetitive Elements
Binding Signal Intensity
CGI n=15,662
Shores n=29,820
Non−CGI Promoter n=83,885
H3K9me3+ regions n=92,573
SINE
LTR
LINE
Alu n=336,724
B2 n=235,891
ERV1 n=45,480
ERVK n=204,585
ERVL n=78,056
ERVL_MaLR n=323,765
L1 n=573,991
L2 n=42,058
Satellite n=19,526
Simple_repeat n=285,639

Supplementary Fig. 7. Unbiased genome-wide mapping unveils a preferential targeting of WT DNMT1 to genomic regions with high-GC contents—notably CpG islands (CGIs)—whereas the CXXC-CpG-interaction-defective mutant (Mut^{KQ}) exhibits the decreased binding to same regions. a Pie charts showing the proportion of the called DNMT1 CUT&Tag peaks that overlap CGIs or non-CGI regions in 1KO-mESCs after reconstitution with either WT DNMT1 or Mut^{KQ}. Green and grey indicate the CGI-overlapping peaks and non-CGI peaks, respectively, with n at the bottom denoting the total number of DNMT1 peaks for each sample (2 biological replicates). **b** Pie charts showing the genomic distribution of the called DNMT1 peaks using HOMER-based annotation in 1KO-mESCs reconstituted with WT DNMT1 or Mut^{KQ}. Features with at least 50 peaks are displayed on the right panel, with 'n' denoting the total number of all peaks. **c** Heatmap depicting the degree of enrichment of binding by the Mut^{KQ} mutant of DNMT1 to regions with the indicated genomic annotation feature, as determined by HOMER. **d** Co-immunoprecipitation (Co-IP) analysis of DNMT1 and UHRF1 in wild-type (WT), DNMT1-knockout (1KO), and Mut^{KQ} cells. **e** Representative immunofluorescence images of DAPI (cyan), EdU (red; 30 min pulse labeling nascent DNA to mark S-phase cells), and DNMT1-FLAG (green) in WT and Mut^{KQ} cells, comparing non-S-phase (EdU-negative) and S-phase (EdU-positive) cells. Merge panels show overlay of all three channels; regions of EdU/DNMT1-FLAG overlap appear yellow. (Bottom right) Intensity profiles of EdU (red) and DNMT1-FLAG (green) signal plotted as a function of distance (μm) across representative S-phase nuclei for WT and Mut^{KQ} cells. Fluorescence intensity was extracted along a square drawn across each nucleus using Fiji/ImageJ. Data are mean ± s.e.m. (n = 10 cells). The mean intensities are shown as solid lines. The intensity values within 1 s.e.m. are shown in shade. **f** DNMT1 binding signal intensities evaluated across distinct genomic features (top panels) and repetitive elements (bottom panels), where each data point represents the mean signal intensity across an individual locus, in 1KO-mESCs reconstituted with WT DNMT1 (blue) or Mut^{KQ} (red). Median values are indicated numerically above each distribution cluster. The total number of evaluated loci (n) is provided beneath each feature name. Statistical significance was determined using a paired Wilcoxon signed-rank test (*****p* < 0.0001). CGI, CpG island; Non-CGI, areas outside CpG islands; SINE, short

interspersed nuclear element; ERV, endogenous retrovirus; LTR, long terminal repeats; LINE (L1/L2), long interspersed nuclear element. Standardized 1-bp chromatin binding signals were mapped across the mm10 genome. Replicates for each condition were merged using a master union matrix. Individual coordinates were intersected with specific genomic annotations: CpG islands/shores, non-CGI promoters (TSS +/- 2kb), major repetitive element families, and H3K9me3+ (enriched) regions derived from public H3K9me3 ChIP-seq data (GSE90893; ChIP: GSM2417112, Input: GSM2417124). Source data are provided with this paper.

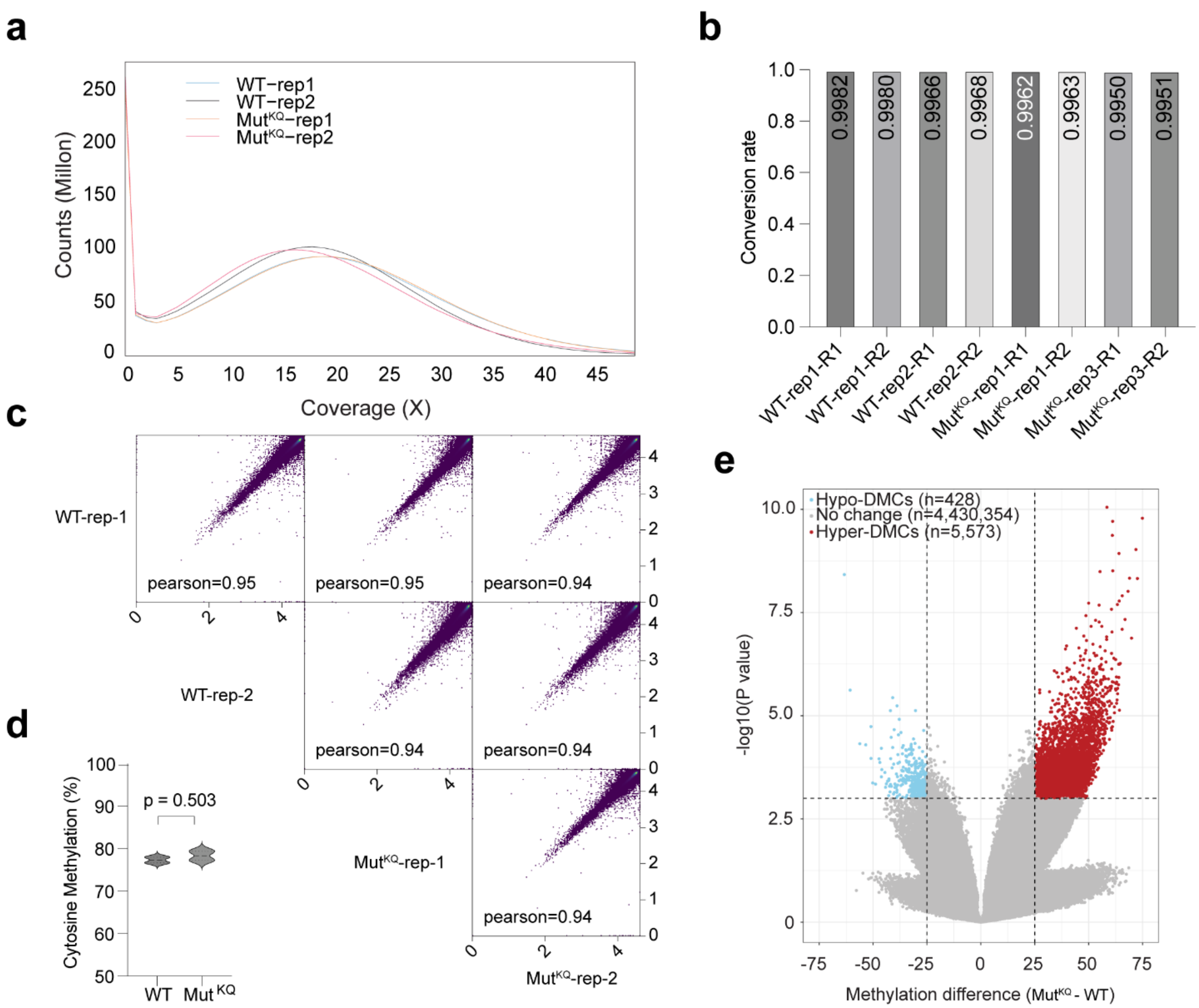

Supplementary Fig. 8. Whole-genome bisulfite sequencing (WGBS) analysis using the 1KO-mESCs reconstituted with DNMT1, either WT or its mutant defective in the CXXC-CpG interaction. a Genome coverage distribution of WGBS data, showing read depth (X) versus genome bin counts (millions) across the biological replicates. **b** Plot showing the bisulfite conversion efficiency (calculated using BCREval script; see Methods) across the biological replicates, demonstrating high conversion efficiency (with conversion rates approaching 1.0 for all replicated samples). **c** Correlation scatter plot of WGBS methylation profiles across the indicated replicated biological samples. **d** Plot of global DNA methylation levels, based on the WGBS profiles of 1KO-ESCs reconstituted with WT DNMT1 or Mut^{KQ}. Two-tailed Student's *t*-tests revealed no significant difference (p = 0.503). **e** Volcano plot illustrating the

differentially methylated CpG sites (DMCs) in cells with the Mut^{KQ} versus WT DNMT1. A total of 6,001 DMCs were detected with a cutoff of *P* value less than 0.001 and methylation difference greater than 25%, which include 5,573 hypermethylated and 428 hypomethylated DMCs. Source data are provided with this paper.

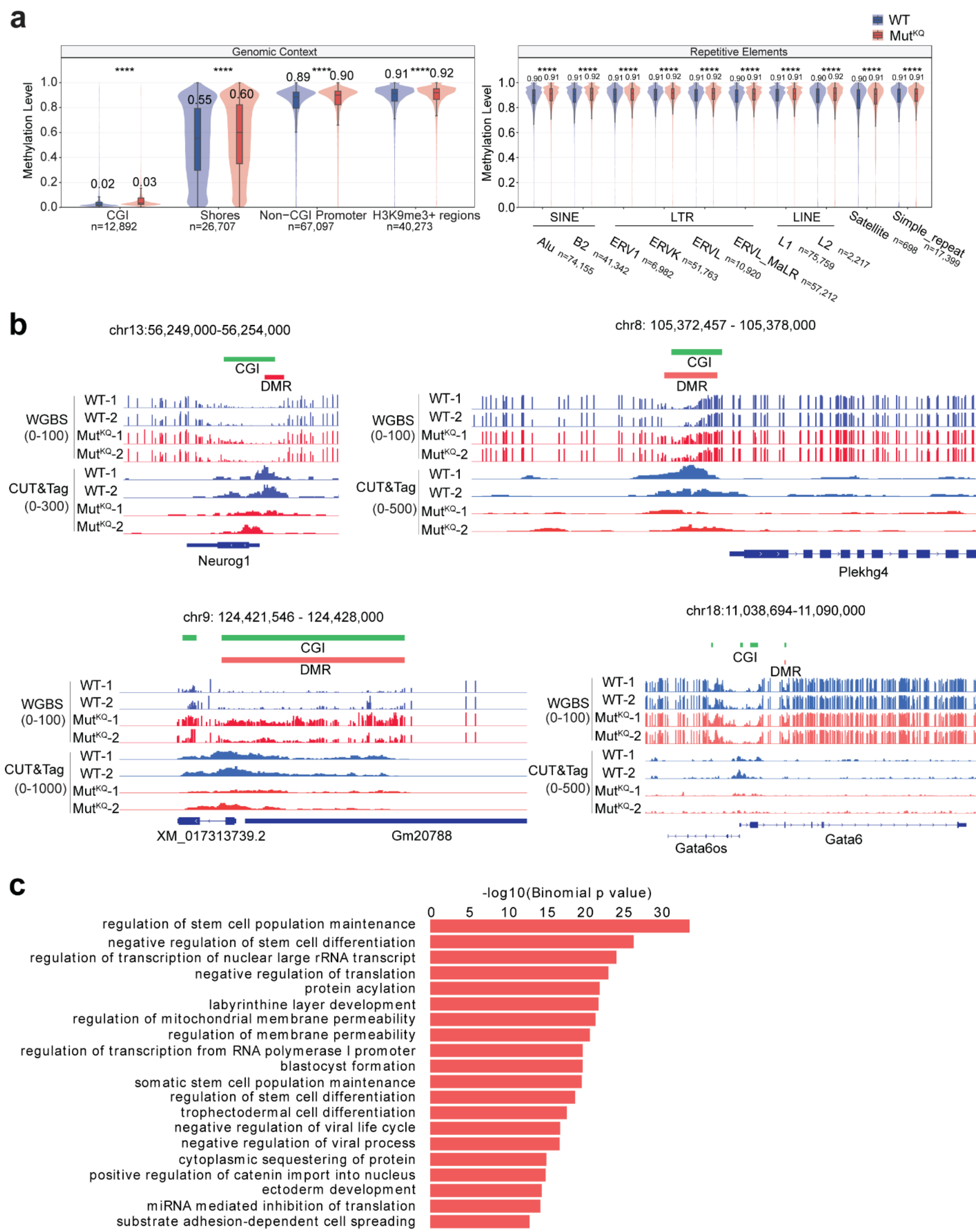

Supplementary Fig. 9. Disruption of the DNMT1 CXXC-CpG interaction induces CGI hypermethylation. a Global DNA methylation levels evaluated across distinct genomic features (left panels) and repetitive elements (right panels) in 1KO-mESCs

reconstituted with WT DNMT1 (blue) or Mut^{KQ} (red). Median values are indicated numerically above each distribution cluster. The total number of evaluated loci (n) is provided beneath each feature name. Statistical significance was determined using a paired Wilcoxon signed-rank test (*****p* < 0.0001). CGI, CpG island; Non-CGI, areas outside CpG islands; SINE, short interspersed nuclear element; ERV, endogenous retrovirus; LTR, long terminal repeats; LINE (L1/L2), long interspersed nuclear element. Single-nucleotide methylation frequencies (>= 10× coverage; SD > 2%) were mapped across the mm10 genome. Replicates for each condition were merged using a master union matrix. Individual coordinates were intersected with specific genomic annotations: CpG islands/shores, non-CGI promoters (TSS +/- 2kb), major repetitive element families, and H3K9me3+ (enriched) regions derived from public H3K9me3 ChIP-seq data (GSE90893; ChIP: GSM2417112, Input: GSM2417124). **b** Integrative Genomic Viewer (IGV) tracks showing DNA methylation (WGBS) and DNMT1 binding (CUT&Tag) at the indicated gene locus in 1KO-mESCs reconstituted with WT DNMT1 or Mut^{KQ} (n = 2 replicated samples). **c** Bar plot depicting enrichment of biological processes for hyper-DMRs, identified by Genomic Regions Enrichment of Annotations Tool (GREAT). The x-axis represents the statistical significance value shown in the -log10 of the binomial *P* value. The analysis highlights pathways involved in stem cell population maintenance and differentiation regulation. Source data are provided with this paper.

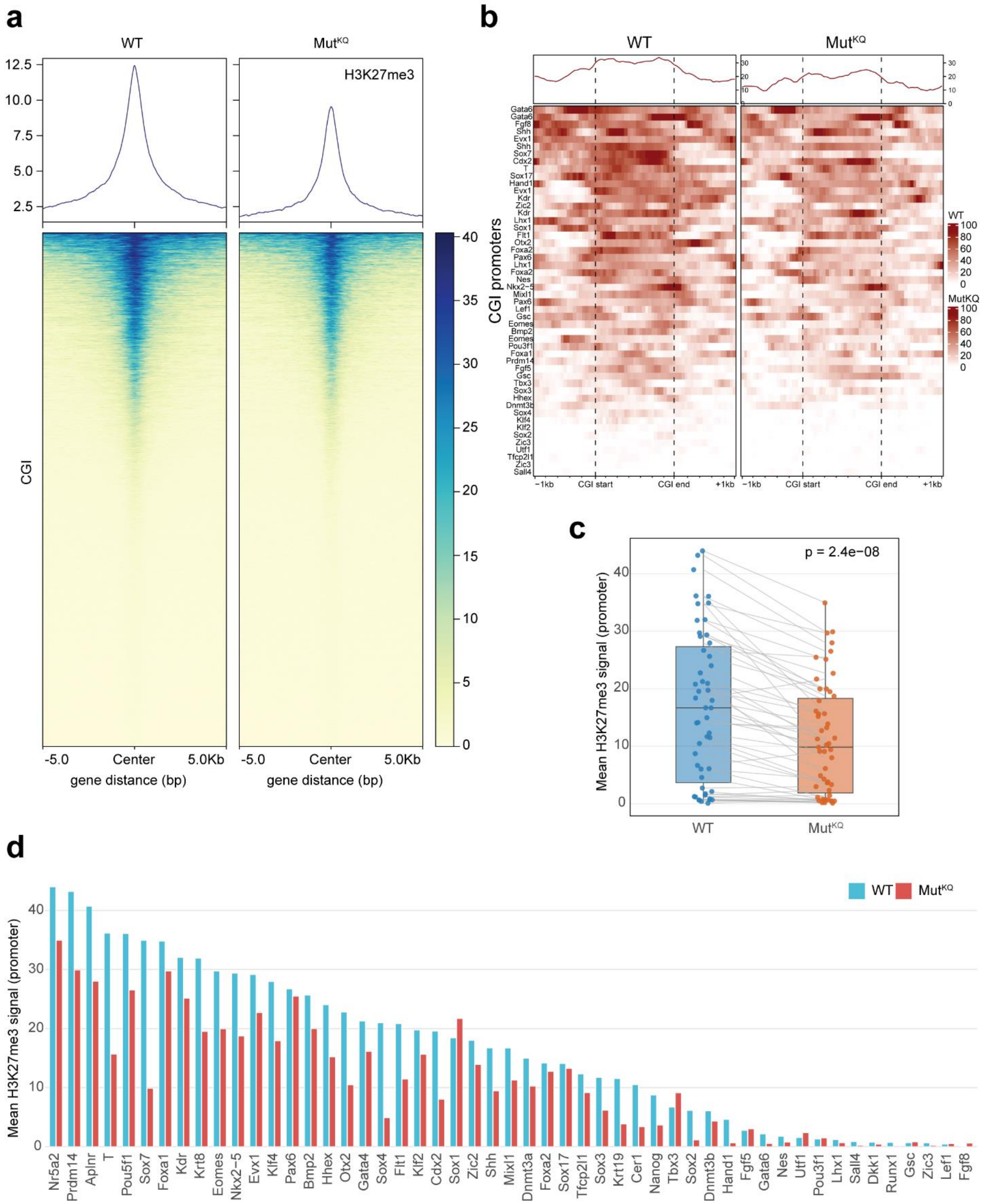

a
WT
Mut^KQ
H3K27me3
12.5
10.0
7.5
5.0
2.5
CGI
40
35
30
25
20
15
10
5
0
-5.0
Center
5.0Kb
gene distance (bp)
b
WT
Mut^KQ
CGI promoters
Gata6
Gata6
Fgf8
Shh
Evx1
Shh
Sox7
Cdx2
T
Sox17
Hand1
Evx1
Kdr
Zic2
Kdr
Lhx1
Sox1
Flt1
Otx2
Foxa2
Pax6
Lhx1
Foxa2
Nes
Nkx2−5
Mixl1
Pax6
Lef1
Gsc
Eomes
Bmp2
Eomes
Pou3f1
Foxa1
Prdm14
Fgf5
Gsc
Tbx3
Sox3
Hhex
Dnmt3b
Sox4
Klf4
Klf2
Sox2
Zic3
Utf1
Tfcp2l1
Zic3
Sall4
−1kb
CGI start
CGI end
+1kb
WT
MutKQ
100
80
60
40
20
0
c
p = 2.4e−08
Mean H3K27me3 signal (promoter)
WT
Mut^KQ
d
WT
Mut^KQ
Mean H3K27me3 signal (promoter)
Nr5a2
Prdm14
Aplnr
T
Pou5f1
Sox7
Foxa1
Kdr
Krt8
Eomes
Nkx2−5
Evx1
Klf4
Pax6
Bmp2
Hhex
Otx2
Gata4
Sox4
Flt1
Klf2
Cdx2
Sox1
Zic2
Shh
Mixl1
Dnmt3a
Foxa2
Sox17
Tfcp2l1
Sox3
Krt19
Cer1
Nanog
Tbx3
Sox2
Dnmt3b
Hand1
Fgf5
Gata6
Nes
Utf1
Pou3f1
Lhx1
Sall4
Dkk1
Runx1
Gsc
Zic3
Lef1
Fgf8

Supplementary Fig. 10. H3K27me3 signal at CGIs and gene promoters in 1KO-mESCs reconstituted with WT DNMT1 or Mut^{KQ}. a Heatmap of H3K27me3 CUT&Tag signal across all CGIs genome-wide in cells with WT DNMT1 and Mut^{KQ}, generated by using deepTools computeMatrix/plotHeatmap, centered on CGIs ± 5 kb. Rows represent individual CGIs; color scale indicates normalized H3K27me3 signal, shared between WT and Mut^{KQ}. **b** Heatmap of H3K27me3 CUT&Tag signals at the CGIs exhibiting overlap with the developmental gene promoters (TSS ± 2 kb), in 1KO-mESCs reconstituted with WT DNMT1 or Mut^{KQ}. Each row is one CGI, generated with EnrichedHeatmap::normalizeToMatrix, flanked by 1 kb on either side of the CGI boundaries (dashed lines: CGI start/end). Rows are ranked by signal intensity in cells with WT DNMT1, from the highest to the lowest, with gene names labeled on the left. Color scales, shown on the right, are consistent between the WT DNMT1 and Mut^{KQ} cells. **c** Boxplot summarizing mean H3K27me3 signal at the promoter (TSS ± 2 kb) of each gene in (b), comparing cells with WT DNMT1 and Mut^{KQ}. Each point represents one gene's promoter; grey lines connect paired WT–Mut^{KQ} values for the same gene. H3K27me3 signal was significantly reduced in cells with Mut^{KQ} versus WT DNMT1 (paired Wilcoxon signed-rank test, $p = 2.4 \times 10^{-8}$). **d** Bar plot of mean H3K27me3 signal at the promoter of each individual gene in the panel, comparing the WT DNMT1 (blue) and Mut^{KQ} (red) cells. Genes (x-axis) are sorted based on signal in cells with WT DNMT1, from high to low. Most genes show reduced H3K27me3 in the Mut^{KQ} cells relative to WT controls. Source data are provided with this paper.

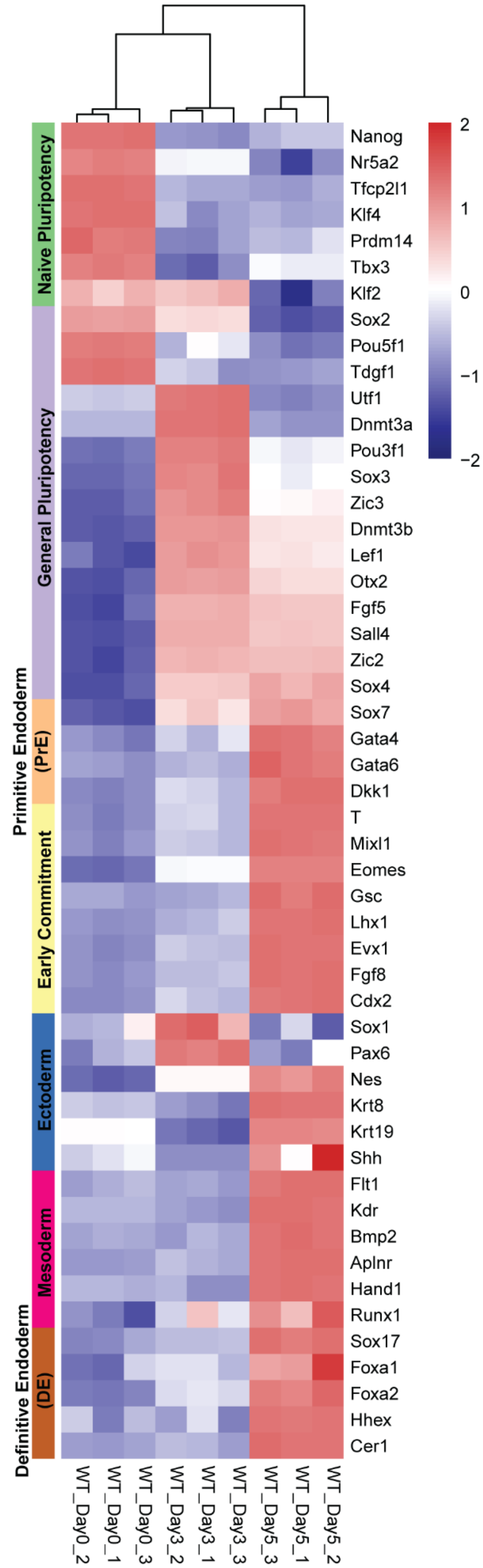
Naive Pluripotency
General Pluripotency
Primitive Endoderm
(PrE)
Early Commitment
Ectoderm
Mesoderm
Definitive Endoderm
(DE)
Nanog
Nr5a2
Tfcp2l1
Klf4
Prdm14
Tbx3
Klf2
Sox2
Pou5f1
Tdgf1
Utf1
Dnmt3a
Pou3f1
Sox3
Zic3
Dnmt3b
Lef1
Otx2
Fgf5
Sall4
Zic2
Sox4
Sox7
Gata4
Gata6
Dkk1
T
Mixl1
Eomes
Gsc
Lhx1
Evx1
Fgf8
Cdx2
Sox1
Pax6
Nes
Krt8
Krt19
Shh
Flt1
Kdr
Bmp2
Aplnr
Hand1
Runx1
Sox17
Foxa1
Foxa2
Hhex
Cer1
WT_Day0_2
WT_Day0_1
WT_Day0_3
WT_Day3_2
WT_Day3_1
WT_Day3_3
WT_Day5_3
WT_Day5_1
WT_Day5_2
2
1
0
−1
−2

Supplementary Fig. 11. Hierarchical clustering and heatmap presentation of z-score normalized expression values based on the log-transformed FPKM data (i.e., $\log_2$(FPKM + 1)) for the indicated lineage specification and pluripotency gene markers across the differentiation time course in the WT DNMT1-expressing cells, establishing an expected baseline kinetics across embryonic layers. Source data are provided with this paper.

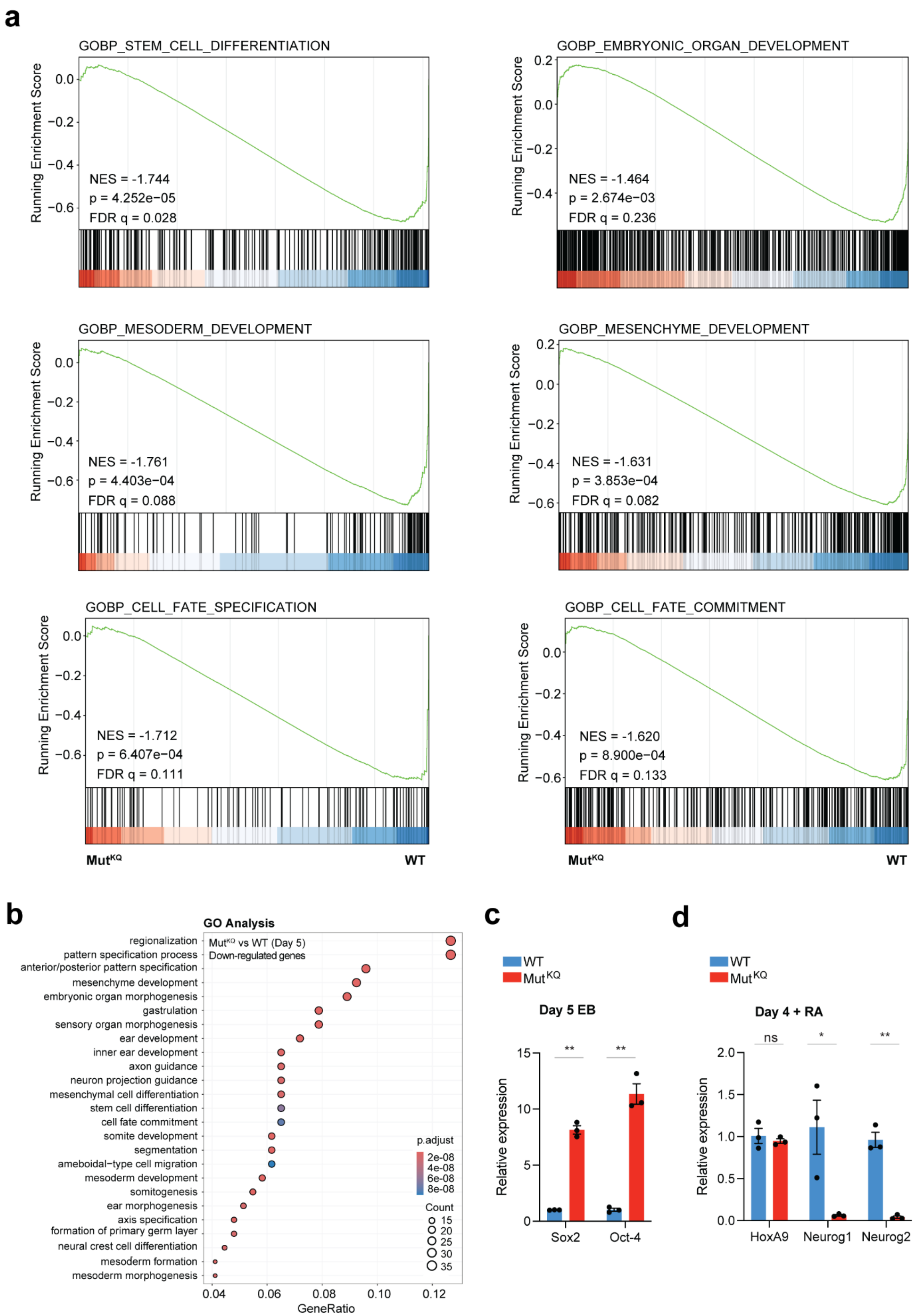
a
GOBP_STEM_CELL_DIFFERENTIATION
NES = -1.744
p = 4.252e−05
FDR q = 0.028
GOBP_EMBRYONIC_ORGAN_DEVELOPMENT
NES = -1.464
p = 2.674e−03
FDR q = 0.236
GOBP_MESODERM_DEVELOPMENT
NES = -1.761
p = 4.403e−04
FDR q = 0.088
GOBP_MESENCHYME_DEVELOPMENT
NES = -1.631
p = 3.853e−04
FDR q = 0.082
GOBP_CELL_FATE_SPECIFICATION
NES = -1.712
p = 6.407e−04
FDR q = 0.111
GOBP_CELL_FATE_COMMITMENT
NES = -1.620
p = 8.900e−04
FDR q = 0.133
Running Enrichment Score
MutKQ
WT
b
GO Analysis
MutKQ vs WT (Day 5)
Down-regulated genes
regionalization
pattern specification process
anterior/posterior pattern specification
mesenchyme development
embryonic organ morphogenesis
gastrulation
sensory organ morphogenesis
ear development
inner ear development
axon guidance
neuron projection guidance
mesenchymal cell differentiation
stem cell differentiation
cell fate commitment
somite development
segmentation
ameboidal−type cell migration
mesoderm development
somitogenesis
ear morphogenesis
axis specification
formation of primary germ layer
neural crest cell differentiation
mesoderm formation
mesoderm morphogenesis
p.adjust
2e-08
4e-08
6e-08
8e-08
Count
15
20
25
30
35
GeneRatio
c
WT
MutKQ
Day 5 EB
Relative expression
Sox2
Oct-4
d
WT
MutKQ
Day 4 + RA
Relative expression
ns
HoxA9
Neurog1
Neurog2

Supplementary Fig. 12. Analysis of genes downregulated in the Mut^{KQ} cells versus WT controls during the embryoid body (EB) differentiation process. a Representative GSEA running enrichment plots for essential multi-lineage and morphogenetic gene sets derived from the MSigDB, including Stem Cell Differentiation, Embryonic Organ Development, Mesoderm Development, Mesenchyme Development, Cell Fate Specification, and Cell Fate Commitment. Corresponding NES, *p*-values, and FDR *q*-values are listed internally. The skewing of the running enrichment score asymmetry toward the right (WT DNMT1 cell-enriched spectrum) represents a systematic and coordinated transcriptional downregulation of primary lineage-commitment networks within the Mut^{KQ} cells. **b** Over-representation analysis (ORA) of significantly downregulated genes (*p*.adjust < 0.05, log_2FoldChange < -0.58) in the Mut^{KQ} versus WT cells at day 5 following EB differentiation, implemented via clusterProfiler::enrichGO mapping against the org.Mm.eg.db mouse genome database (Ontology: Biological Process). The x-axis represents the Gene Ratio, dot size scales with absolute gene count, and point color corresponds to Benjamini-Hochberg (BH) adjusted p-values (p.adjust). **c** RT–qPCR of the indicated pluripotency gene, at day 5 post-differentiation using EBs formed by mESCs with WT DNMT1 or the Mut^{KQ} mutant. Data represent mean ± s.e.m. from three independent experiments. *P* values were determined by two-tailed Student's *t*-test. **d** RT–qPCR of the indicated neuronal lineage marker gene, at day 4 following retinoid acid (RA)-induced differentiation of mESCs with WT DNMT1 or the Mut^{KQ} mutant. Data represent mean ± s.e.m. from three independent experiments. *P* values were determined by two-tailed Student's *t*-test. ns, not significant; $^*p < 0.05$; $^{**}p < 0.01$. Source data are provided with this paper.

Supplementary Table 1. Cryo-EM data collection, refinement and validation statistics of WT DNMT1-^{um}NCP and Mut^{MHKKKQ} DNMT1-^{um}NCP complexes

	DNMT1-^{um}NCP	Mut^{MHKKKQ} DNMT1-^{um}NCP	
			With RFTS
PDB Codes	9Z07	9MYS	
EMDB codes	EMD-73698 (Composite), EMD-73713 (Consensus), EMD-73701 (Local DNMT1), EMD-73708 (Local CXXC-NCP).	EMD-48749 (Composite), EMD-48748 (Consensus), EMD-77709 (Local), EMD-77710 (Local)	EMD-77711
**Data collection and processing**			
Microscope	Titan Krios		
Camera	Gatan K3		
Magnification	81,000x		
Voltage (kV)	300		
Defocus range (µm)	-1.5 ~ -2.1	-0.8 ~ -2.5	
Exposure time (s)	2.0	2.5	
Dose rate(e–/Å2/s)	25.0	26.20	
Number of frames	40	50	
Pixel size (Å)	1.07	1.07	
Micrographs (no.)	16,915	31,434	
Symmetry imposed	C1	C1	
Initial particles (no.)	6,950,000	7,940,000	
Final particles (no.)	417,039	287,638	72,038
Map resolution (Å)	4.86-3.02	5.24-3.60	4.13
FSC threshold	0.143	0.143	0.143
**Refinement**			
Refinement method	Real_space_refine in PHENIX		
Initial model used	PDB 7JZV,4WXX,3PTA		
Model resolution (Å)	3.19	3.93	
FSC threshold	0.5	0.5	
Map sharpening B factor (Å^2)	-30 (Local CXXC-NCP)	-100 (Consensus); -76, -200 (Local)	-80
CC (mask)	0.72	0.76	
Model composition			
Non-hydrogen atoms	17,917	19,131	
Protein residues	1,628	1,672	
Nucleotides	298	298	
Ion (zinc)	4	4	
B factors (Å^2)			
Protein	89.98	134.36	
Nucleotides	170.52	99.83	
Ion (zinc)	224.35	177.85	
R.m.s. deviations			
Bond lengths (Å)	0.004	0.004	
Bond angles (°)	0.609	0.776	
Validation			
MolProbity score	1.73	1.93	
Clashscore	10.13	15.23	
Poor rotamers (%)	1.38	0.89	
Ramachandran plot			
Favored (%)	97.50	96.29	
Allowed (%)	2.50	3.71	
Disallowed (%)	0	0	

Supplementary Data 1. Summary of genomic annotation and statistics using the called DNMT1 CUT&Tag peaks, either wild-type (WT) or the K683A/Q684A mutant (Mut^{KQ}), reconstituted in the Dnmt1-knockout (1KO) mESCs (n = 2 biological replicates). Genomic feature enrichment analysis was performed using HOMER, which applies a one-sided binomial test to determine whether features are significantly enriched relative to the expected background genome distribution.

Supplementary Data 2. Summary of the whole-genome bisulfite sequencing (WGBS) dataset collected from the 1KO-mESCs reconstituted with DNMT1, either WT or the Mut^{KQ} mutant (n = 2 biological replicates), such as total number of read tags, coverages, read mappability, and bisulfite conversion rate. WGBS reads were mapped to reference genome (mm10) by using bismark while the Bisulfite conversion rate (BCR) calculation was based on the telomeric repetitive DNA as native spike-in control (using BCR_Evaluator).

Supplementary Data 3. Genomic annotation and enrichment score calculation using differentially methylated CpG sites (DMCs) exhibiting significant methylation increase in 1KO-mESCs with the Mut^{KQ} versus WT DNMT1 (termed as hyper-DMCs). DMC is called based on a standard of adjusted P value less than 1×10^{-4} and difference in methylation levels greater than 25%. Genomic feature enrichment analysis was performed using HOMER, which applies a one-sided binomial test to determine whether features are significantly enriched relative to the expected background genome distribution.

Supplementary Data 4. Statistical analysis to compare the degree of methylation changes (delta values, based on WGBS of 1KO-mESCs with WT DNMT1 versus the MutKQ mutant) at the two indicated genomic regions with different annotation features. *P* values and adjusted *P* values were calculated using a two-sided Wilcoxon signed-rank test for paired comparisons, with Benjamini-Hochberg adjustment for multiple comparisons (**** $P < 0.0001$).

Supplementary Data 5. Genomic annotation and enrichment score calculation using differentially methylated regions (DMRs) exhibiting significant methylation increase in 1KO-mESCs with the Mut^{KQ} versus WT DNMT1 (termed as hyper-

DMRs). Genomic feature enrichment analysis was performed using HOMER, which applies a one-sided binomial test to determine whether features are significantly enriched relative to the expected background genome distribution.

Supplementary Data 6. Information for the primers used in this study.

Supplementary Data 7. List of nucleosome DNAs used in this study.
