# Supplementary material for: The DNMT1 CXXC domain senses CpG islands for DNA methylation inhibition
Source: Nat Commun. 2026 Sep 18;17:10970. doi: 10.1038/s41467-026-77872-8 (PMC13683344; doi:10.1038/s41467-026-77872-8)
Supplement: Supplementary file 4 [file 41467_2026_77872_MOESM2_ESM.pdf]

Supplementary Data 1. Summary of genomic annotation and statistics using the called DNMT1 CUT&Tag peaks, either wild-type (WT) or the K683A/Q684A mutant (Mut^{KQ}), reconstituted in the Dnmt1-knockout (1KO) mESCs (n = 2 biological replicates). Genomic feature enrichment analysis was performed using HOMER, which applies a one-sided binomial test to determine whether features are significantly enriched relative to the expected background genome distribution.

Supplementary Data 2. Summary of the whole-genome bisulfite sequencing (WGBS) dataset collected from the 1KO-mESCs reconstituted with DNMT1, either WT or the Mut^{KQ} mutant (n = 2 biological replicates), such as total number of read tags, coverages, read mappability, and bisulfite conversion rate. WGBS reads were mapped to reference genome (mm10) by using bismark while the Bisulfite conversion rate (BCR) calculation was based on the telomeric repetitive DNA as native spike-in control (using BCR_Evaluator).

Supplementary Data 3. Genomic annotation and enrichment score calculation using differentially methylated CpG sites (DMCs) exhibiting significant methylation increase in 1KO-mESCs with the Mut^{KQ} versus WT DNMT1 (termed as hyper-DMCs). DMC is called based on a standard of adjusted P value less than 1×10^{-4} and difference in methylation levels greater than 25%. Genomic feature enrichment analysis was performed using HOMER, which applies a one-sided binomial test to determine whether features are significantly enriched relative to the expected background genome distribution.

Supplementary Data 4. Statistical analysis to compare the degree of methylation changes (delta values, based on WGBS of 1KO-mESCs with WT DNMT1 versus the MutKQ mutant) at the two indicated genomic regions with different annotation features. *P* values and adjusted *P* values were calculated using a two-sided Wilcoxon signed-rank test for paired comparisons, with Benjamini-Hochberg adjustment for multiple comparisons (**** $P < 0.0001$).

Supplementary Data 5. Genomic annotation and enrichment score calculation using differentially methylated regions (DMRs) exhibiting significant methylation increase in 1KO-mESCs with the Mut^{KQ} versus WT DNMT1 (termed as hyper-

DMRs). Genomic feature enrichment analysis was performed using HOMER, which applies a one-sided binomial test to determine whether features are significantly enriched relative to the expected background genome distribution.

Supplementary Data 6. Information for the primers used in this study.

Supplementary Data 7. List of nucleosome DNAs used in this study.
